# Coordination Chemistry of a Bis(Tetrazine) Tweezer: A Case of Host-Guest Behavior with Silver Salts

**DOI:** 10.3390/molecules26092705

**Published:** 2021-05-05

**Authors:** Clève D. Mboyi, Ons Amamou, Paul Fleurat-Lessard, Julien Roger, Hélène Cattey, Charles H. Devillers, Michel Meyer, Taoufik Boubaker, Jean-Cyrille Hierso

**Affiliations:** 1 Institut de Chimie Moléculaire de l’Université de Bourgogne (ICMUB—UMR CNRS 6302), Université Bourgogne Franche-Comté (UBFC), 9 avenue Alain Savary, 21078 Dijon CEDEX, France; Cleve-Dionel.Mboyi@u-bourgogne.fr (C.D.M.); amamouons1995@gmail.com (O.A.); Paul.Fleurat-Lessard@u-bourgogne.fr (P.F.-L.); julien.roger@u-bourgogne.fr (J.R.); helene.cattey@u-bourgogne.fr (H.C.); charles.devillers@u-bourgogne.fr (C.H.D.); michel.meyer@u-bourgogne.fr (M.M.); 2 Laboratoire de Chimie Hétérocyclique, Produits Naturels et Réactivité (LR11S39), Faculté des Sciences, Université de Monastir, Avenue de l’Environnement, 5019 Monastir, Tunisia; boubaker_taoufik@yahoo.fr

**Keywords:** bis(tetrazine), coordination, host-guest, ligand, silver, XRD structure

## Abstract

The carbon-carbon cross-coupling of phenyl *s*-tetrazine (Tz) units at their *ortho*-phenyl positions allows the formation of constrained bis(tetrazines) with original tweezer structures. In these compounds, the face-to-face positioning of the central tetrazine cores is reinforced by π-stacking of the electron-poor nitrogen-containing heteroaromatic moieties. The resulting tetra-aromatic structure can be used as a weak coordinating ligand with cationic silver. This coordination generates a set of bis(tetrazine)-silver(I) coordination complexes tolerating a large variety of counter anions of various geometries, namely, PF_6_^−^, BF_4_^−^, SbF_6_^−^, ClO_4_^−^, NTf_2_^−^, and OTf^−^. These compounds were characterized in the solid state by single-crystal X-ray diffraction (XRD) and diffuse reflectance spectroscopy, and in solution by ^1^H-NMR, mass spectrometry, electroanalysis, and UV-visible absorption spectrophotometry. The X-ray crystal structure of complexes {[Ag(**3**)][PF_6_]}_∞_ (**4**) and {[Ag(**3**)][SbF_6_]}_∞_ (**6**), where **3** is 3,3′-[(1,1′-biphenyl)-2,2′-diyl]-6,6′-bis(phenyl)-1,2,4,5-tetrazine, revealed the formation of 1D polymeric chains, characterized by an evolution to a large opening of the original tweezer and a coordination of silver(I) via two chelating nitrogen atom and some C=C π-interactions. Electrochemical and UV spectroscopic properties of the original tweezer and of the corresponding silver complexes are reported and compared. ^1^H-NMR titrations with AgNTf_2_ allowed the determination of the stoichiometry and apparent stability of two solution species, namely [Ag(**3**)]^+^ and [Ag(**3**)_2_]^2+^, that formed in CDCl_3_/CD_3_OD 2:1 *v*/*v* mixtures.

## 1. Introduction

**Figure 1:**
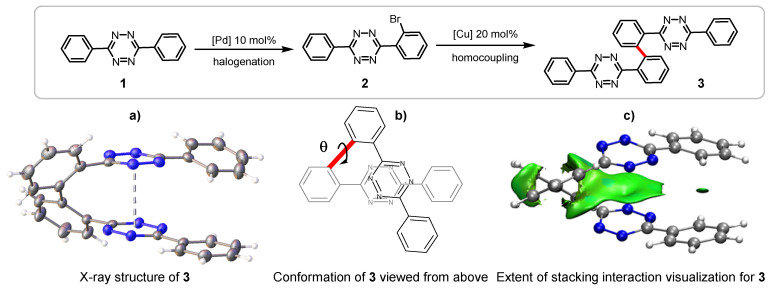
Bridge clamp bis(tetrazine) synthesis, (**a**) XRD structure, (**b**) Definition of the dihedral angle, and (**c**) intramolecular stacking interaction. Atom color code: carbon in grey, nitrogen in blue, and hydrogen in white. The green areas illustrate the extent of the stacking interaction.

The *s*-tetrazine (Tz) unit (Figure 1, **1**) is an object of high interest in photophysics and for biomedical applications, owing to its electron-poor poly(hetero)aromatic nature that is also amenable to click chemistry [1,2,3]. The diversification of synthetic routes to conceive new tetrazine structures has become very useful to expand current applications [3,4,5,6,7,8,9]. We recently reported a copper-catalyzed homocoupling protocol, which allowed the efficient synthesis of constrained bis(tetrazines) with a unique bridge clamp structure (Figure 1, **3**) [3]. These compounds have been studied in the solid state by X-ray diffraction (XRD, Figure 1a) and analyzed by DFT. The results have shown that their bridge clamp structure (Figure 1b) is due to the unexpected existence of stacking interactions between the [C_2_N_4_]·[C_2_N_4_] electron-poor central tetrazine cores (Figure 1c). We have established that London dispersion forces between the Tz moieties clearly stabilize and favor the clamp geometry. In their absence, the tweezer-like interconversion barrier between the most stable rotamers corresponding to a rotation of both tetrazine cores around the *ortho*-C–C bond (stacked, with an angle *θ* = 54°, or *gauche* with *θ* = 123°) would be as low as 2 kcal mol^−1^. Conversely, their presence enhances the transition structure barrier up to 6.5 kcal mol^−1^, making the interconversion opening of the clamp toward the *gauche* rotamer much less favorable.

*s*-Tetrazine derivatives may be successfully used as ligands for transition metals [10,11]. In comparison, the coordination properties of bis(tetrazines) have been rarely studied [12,13,14,15]. Herein, we investigated the binding properties of bis(tetrazine) **3** with various metals, focusing on the Ag^+^ cation, thus assessing the global stability of the tweezer structure and its degree of flexibility around the *ortho*-C–C bond. We found that **3** is a weak ligand of several transition metals in non-coordinating low polarity solvents. Interactions with silver(I) salts generate bis(tetrazine)-silver(I) coordination polymers, which tolerate a large variety of counter anions including PF_6_^−^, BF_4_^−^, SbF_6_^−^, ClO_4_^−^, NTf_2_^–^, and OTf^−^. Accordingly, the XRD structures of complexes {[Ag(**3**)][PF_6_]}_∞_ (**4**) and {[Ag(**3**)][SbF_6_]}_∞_ (**6**) revealed the formation of 1D polymeric chains characterized by a large opening of the original tweezer structure (with *θ* = 144.0(2)° for **4** and 145.20(3)° for **6**, vs. *θ* = 54° in **3**). Electrochemical behavior and UV spectroscopic characterization of the tweezer and its silver complexes are reported, and further studies in solution focused on the ^1^H-NMR of the bis(tetrazine) **3** in comparison with the related mono(tetrazine) **1** and the formation of the bis(tetrazine)-silver(I) coordination polymers **4** and **6**. Our study showed a typical tweezer-Ag^+^ host-guest interaction behavior in solution, which could be further exploited for easy anion exchange.

## 2. Results and Discussion

Bis(tetrazine) **3** and its functionalized derivatives display in the solid state a centroid-to-centroid separation of the central rings ranging between 3.3 and 3.5 Å (Figure 1) [3]. Given this structure and the presence of eight nitrogen atoms as potential coordinating donors, we were intrigued about the potential coordination properties of this new bridge clamp structure with transition metals. Would it be possible that a sandwich-like association reminding classical metallocenes and hemi-metallocenes could form? Moreover, does the framework retain sufficient conformational freedom to accommodate a large range of coordination modes with various metals? With the view of addressing these issues, we conducted comparative coordination chemistry studies of the bis(tetrazine) **3** in solution and in the solid state.

### 2.1. Bis(Tetrazine) **3** for Metal Complexation

**Scheme 1:**
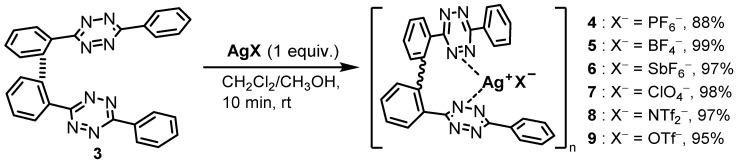
Synthesis of cationic silver(I) complexes **4**–**9** obtained from the stoichiometric reaction of AgX salts (X = PF_6_^−^, BF_4_^−^, SbF_6_^−^, ClO_4_^−^, NTf_2_^−^, and OTf^−^) with the bis(tetrazine) **3**.

The silver(I) cation is a suitable candidate for metal complexation because it can adopt several coordination numbers and geometries. Moreover, as a soft cation Ag^+^ is known to interact with tetrazine ligands [16,17]. The reaction of the bis(tetrazine) **3** with one equivalent of AgPF_6_ in a CH_2_Cl_2_/MeOH mixture (2:1 *v/v*) proceeds rapidly, affording in 88% yield an air-stable, soluble red-orange complex, denoted [Ag(**3**)]PF_6_, (**4** in Scheme 1). When the silver salt was used in excess compared to **3**, an orange solid was formed that turned out to be insoluble in CH_2_Cl_2,_ suggesting the formation of polymeric species.

The complexes **5**–**9** (Scheme 1) were isolated quantitatively upon reacting **3** with a variety of AgX salts (where X = BF_4_^−^, SbF_6_^−^, ClO_4_^−^, NTf_2_^−^, and OTf^−^, respectively) under similar conditions. In all cases, the use of an excess of Ag(I) salt led to the formation of poorly soluble aggregates that hampered detailed characterization in solution. The binding of the bis(tetrazine) **3** to other transition metal cations, such as Cu^+^ and Pd^2+^, was also investigated. [Cu(CH_3_CN)_4_]BF_4_, [Pd(CH_3_CN)_2_Cl_2_], and [Pd(CH_3_CN)_4_](BF_4_)_2_ were used as metal sources and clearly appeared to readily react with **3**. The complex denoted [Cu(**3**)]BF_4_ was analyzed in solution by NMR spectroscopy (details in SI), but the compounds resulting from the reaction with palladium (tentatively denoted [Pd(**3**)]^+^ and [Pd(**3**)Cl]^+^ according to mass-spectrometric analyses) were poorly soluble and could not be easily characterized by NMR spectroscopy in solution. However, HR-mass spectrometry could be conducted, and ESI-MS spectra exhibited peaks at *m*/*z* = 529, 607, and 572 a.m.u. with an isotopic distribution pattern consistent with [Cu(**3**)]^+^, [Pd(**3**)]^+^, and [Pd(**3**)Cl]^+^, respectively (details in SI). Conversely, our attempts to isolate and fully characterize those complexes formed by reacting **3** with the zerovalent precursors [Pd_2_(dba)_3_] and Cr(CO)_6_ failed, obviating the eventuality of stabilizing metallocene-like complexes by the controlled opening of tweezer **3**.

### 2.2. Solid-State Characterization of Silver(I) Complexes

**Figure 2:**
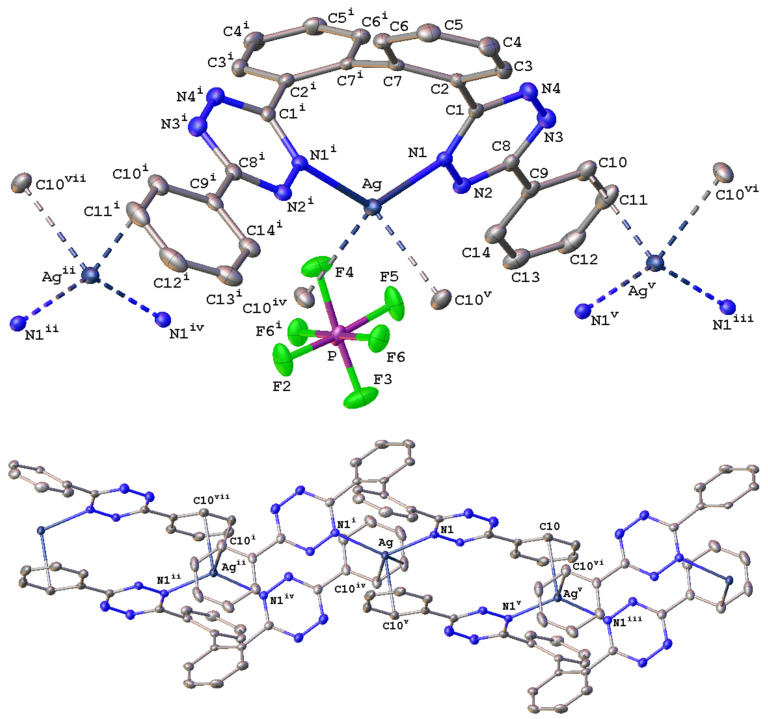
Top: Thermal ellipsoid representation of the polymer fragment of {[Ag(**3**)][PF_6_]}_∞_ (**4**) at the 50% probability level (for clarity, the chloroform and H atoms are omitted). Selected bond distances in (Å) and angle values in (°): Ct1(C1-N1-N2-C8-N3-N4)···Ct1^i^ 6.6846(9), Ct1···Ct1^v^ (C1^v^-N1^v^-N2^v^-C8^v^-N3^v^-N4^v^) 4.4091(9), Ag–N1 and Ag–N1^i^ 2.3163(15), Ag···C10^v^ and Ag···C10^iv^ 2.7014(18); N1–Ag–N1^i^ 127.36(7), N1–Ag–C10^v^ 99.75(5), N1–Ag–C10^iv^ 120.05(5), C10^iv^–Ag–C10^v^ = 81.86(9). Symmetry codes: (i) ½ − *x*, 1 − *y*, *z*; (ii) −*x*, 1 − *y*, 1 − *z*; (iii) ½ + *x*, *y*, 1 − *z*; (iv) −½ + *x*, *y*, 1 −*z*; (v) 1 − *x*, 1 − *y*, 1 − *z*; (vi) ^3^/_2_ − *x*, 1 − *y*, *z*; (vii) *x* − 1, *y*, *z*. Bottom: portion of the packing diagram of {[Ag(**3**)][PF_6_]}_∞_ (**4**) depicting the tetrazine ring centroids arrangement; PF_6_^–^ anions are omitted for clarity.

**Figure 3:**
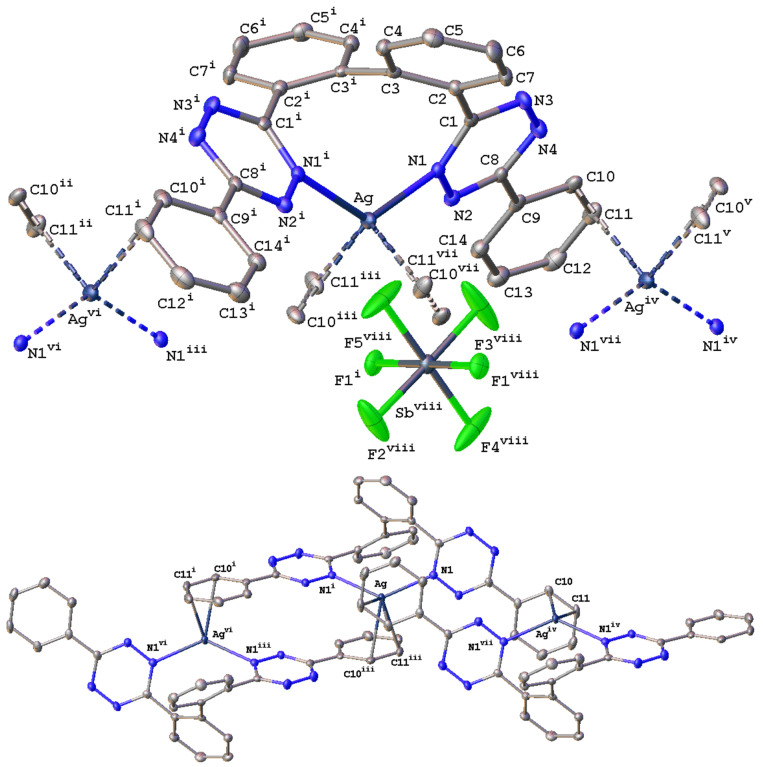
Top: Thermal ellipsoid representation of the polymer fragment of {[Ag(**3**)][SbF_6_]}_∞_ (**6**) at the 50% probability level (for clarity, the chloroform and H atoms are omitted). Selected bond distances in (Å) and angle values in (°): Ct1(C1-N1-N2-C8-N3-N4)···Ct1^i^ 6.6895(16), Ct1···Ct1^vii^ 4.4208(17), Ag–N1 and Ag–N1^i^ 2.320(3), Ag···C10^iii^ and Ag···C10^vii^ 2.686(3), Ag···C1^iii^ and Ag···C1^vii^ 2.698(4); N1–Ag–N1^i^ 127.39(14), N1–Ag–C10^vii^ 99.20(10), N1–Ag–C11^vii^ 114.74(10), N1–Ag–C10^iii^ 40.26(7), N1–Ag–C11^iii^ 38.13(7). Symmetry codes: (i) *x*, ^3^/_2_ − *y*, 1 − z; (ii) −*x*, 1 + *y*, *z*; (iii) 1 − *x*, ½ + *y*, *z*; (iv) 1 − *x*, *y* − ½, *z*; (v) *x*, ½ − *y*, 1 − *z*; (vi) 1 − *x*, 2 − *y*, 1 − *z*; (vii) 1 − *x*, 1 − *y*, 1 − *z*; (viii) *x*, ^3^/_2_ − *y*, *z* − ½. Bottom: portion of the packing diagram of {[Ag(**3**)][SbF_6_]}_∞_ (**6**) depicting the tetrazine ring centroids arrangement; SbF_6_^–^ anions are omitted for clarity.

Single crystals of complexes **4** or **6** were obtained by slow evaporation of their solution in dichloromethane and in a chloroform/pentane mixture, respectively. The crystallographic structure of those complexes (Figure 2 and Figure 3) was solved in the orthorhombic *Pnna* and *Pbcm* space groups, for **4** and **6**, respectively. In these crystal structures, the bis(tetrazine) **3** adopts an open shape where the two tetrazine rings are interconnected through the coordination of a silver(I) cation by the doublets of N1 nitrogen atoms. This arrangement formed a rare nine-membered metallacycle. In the solid state, the bis(tetrazine) **3** displayed a torsion angle between the two Tz moieties equal to 51.1(3)°. Upon silver binding, the corresponding C2-C7-C7^i^-C2^i^ torsion angle opens up to 144.0(2)° for **4**, and the analogous C2-C3-C3^i^-C2^i^ torsion angle is consistently found equal to 145.2(3)° for **6**. Accordingly, the distance between the two C_2_N_4_ centroids (Ct) in the Tz changed from 3.320(1) Å in **3** to 6.6846(9) Å in **4** and 6.6895(16) Å in **6** after silver coordination. In the complexes, the Tz cores in the monomer unit are roughly perpendicular to each other, with angles equal to 76.73° for **4** and 78.05° for **6**, and each of them is parallel to the tetrazine ring of a neighboring monomer (see Figure 2 and Figure 3). Complexes **4** and **6** exhibit polymeric organization, wherein [Ag(**3**)]PF_6_ or [Ag(**3**)]SbF_6_ constitutes the repeating unit. The Ag atom is linked to two nitrogen atoms N1 located nearby the central *o*-linked phenyl groups, with Ag–N1 distances that are, respectively, equal to 2.3165(15) Å for **4** and 2.320(3) Å for **6**.

**Figure 4:**
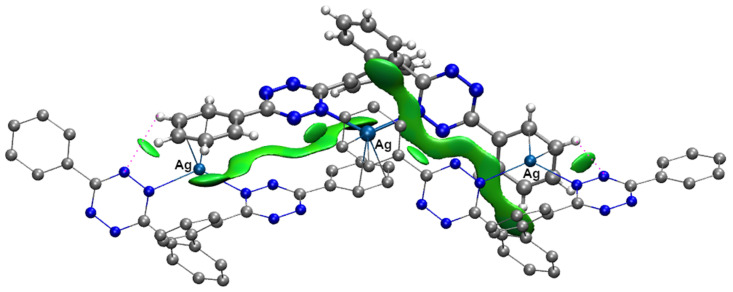
Visualization of the non-covalent interactions in the {[Ag(**3**)][SbF_6_]}_∞_ (**6**) coordination polymer. The green zones correspond to dispersion interactions between two [Ag(**3**)] units. The pink dashed lines indicate weak CH···N interactions.

In the polymer chain of complex **4**, silver atoms interact through two similar cation-π interactions with the two nearest neighboring phenyl groups obtained by symmetry operations: (iv): −½ + *x*, *y*, 1 − *z* and (v) 1 − *x*, 1 − *y*, 1 − *z*. The Ag^+^ cation is located just above C10, with a mutual distance of *d* = 2.7013(19) Å, in an η^1^-coordination. The angles around the silver cation are those of a distorted tetrahedron: N1–Ag–N1^i^ = 127.36(7)°, N1–Ag–C10^v^ = 99.75(5)°, N1–Ag–C10^iv^ = 120.05(5)° and C10^iv^–Ag–C10^v^ = 81.86(9)°. As shown in Figure 4, supplementary π–π type interactions are present between two parallel tetrazine moieties from neighboring monomers that further stabilize the crystalline assembly. The interplanar and the centroid-to-centroid distances between parallel tetrazine moieties are equal to 3.159(9) and 4.4091(9) Å, respectively. The difference found in these two distances indicates that the tetrazine rings experience a strong mutual slippage, with an angle between the normal to the planes and the centroid-centroid vector of 44.24° corresponding to a slippage distance of ca. 3 Å. In the literature, the interplanar distance between the arene planes is commonly found around 3.3 and 3.8 Å for such interactions [18].

Similarly, in the polymer chain of complex **6**, silver atoms interact with the two phenyl rings obtained by symmetry operations: (iii) 1 − *x*, ½ + *y*, *z* and (vii) 1 − *x*, 1 − *y*, 1 − *z*. However, the Ag^+^ cation herein coordinates two carbon atoms (C10 and C11) of the same phenyl ring in an η^2^-coordination [19,20]. The two bond lengths are close, with *d* = 2.686(3) Å for Ag···C10^iii^ and Ag···C10^vii^, and *d* = 2.698(4) Å for Ag···C11^iii^ and Ag···C11^vii^. Silver atoms in complex **6** present the same distorted tetrahedral arrangement as in complex **4**, with analogous angles. In this complex, the interplanar and the centroid-to-centroid distances between parallel tetrazines are equal to 3.206(17) and 4.4208(17) Å, respectively. The tetrazine rings show a mutual slippage angle equal to 43.52° and a related slippage distance of *ca* 3 Å.

### 2.3. Characterization of the Bis(Tetrazine) **3** and of its Silver Complexes ***4**–**9*** in Solution

#### 2.3.1. ^1^H-NMR Spectroscopy

**Figure 5:**
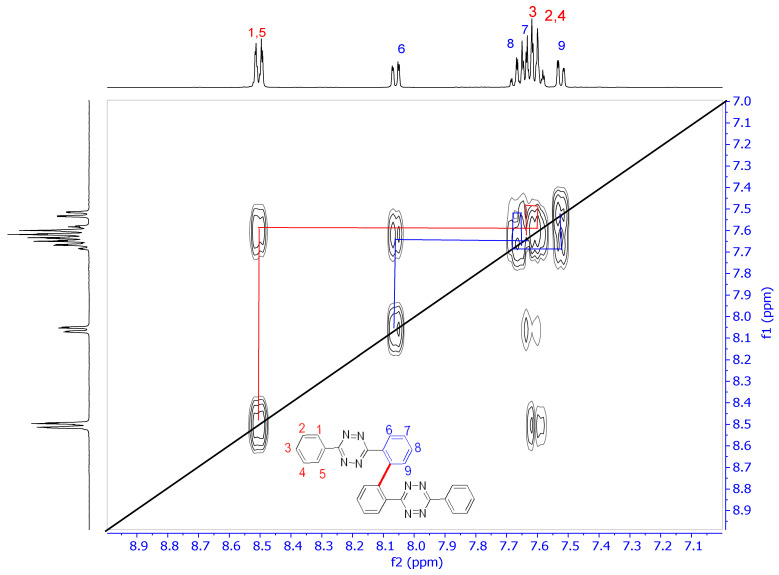
COSY 1H-NMR (400 MHz, 298 K) spectrum of bis(tetrazine) 3 in CD_2_Cl_2_.

The NMR spectra of the bis(tetrazine) **3** and of representative silver complexes are reported in Supporting Information (SI). For unambiguous attribution, bidimensional correlation spectra (COSY, NOESY, HMQC, and HMBC) were recorded in CD_2_Cl_2_ at 298 K. The COSY chart of the bis(tetrazine) **3** depicted in Figure 5 enabled to observe four distinguishable groups of signals located at 8.50 (4H), 8.06 (2H), 7.68–7.58 (10H), and 7.52 ppm (2H) attributed to H_1,5_, H_6_, H_2,3,4,7,8_, and H_9_, respectively. The proximity of the electron-withdrawing tetrazine core induces resonances at lower fields for H_1,5_ compared to H_2,3,4_, and for H_6_ compared to H_7,8_. Conversely, the electron-donating *o*-Ph that is proximate to H_9_ induces a higher field resonance for these nuclei compared to H_7,8_.

^1^H-NMR spectra of **3** were also recorded in a series of solvents of various polarities, including toluene-*d*_8_, CDCl_3_, THF-*d*_8_, (CD_3_)_2_CO, CD_3_CN, and (CD_3_)_2_SO. The same spectral profile was observed in CDCl_3_ and CD_2_Cl_2_, but noticeable splitting and shifts were observed with the other solvents (Figure S1). More polar solvents such as CD_3_CN or DMSO-*d*_6_—but also toluene—showed prominent shifts for most of the resonances, suggesting solvent-induced structural changes in **3**. These could be related either to the change of the equilibrium value of the twist-angle *θ*, since intramolecular *π*–*π* interactions are less favored in more polar media, or some solvent–*π* intercalation in toluene. Accordingly, the molecular tweezer was predicted to possibly open up, i.e., widening of the *θ* angle, in such dissociating solvents.

**Figure 6:**
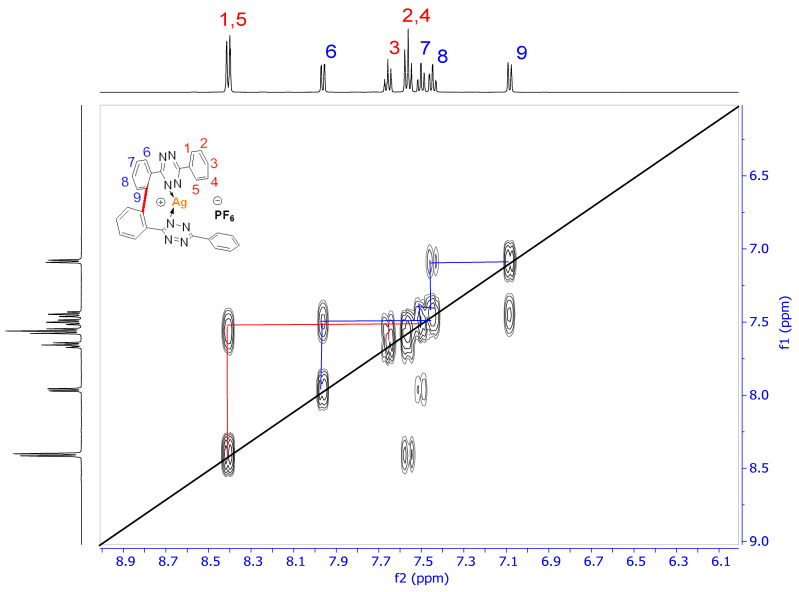
COSY ^1^H-NMR (400 MHz, 298 K) spectrum of complex **4** in CD_2_Cl_2_.

While the ^1^H-NMR spectrum of the uncoordinated compound **3** in CDCl_3_ exhibits seven overlapping signals (H_1,5_, H_2,4_, H_3_, H_6_, H_7_, H_8_, and H_9_) at room temperature in agreement with its symmetry, this set of resonances is split in CDCl_3_ for the corresponding bis(tetrazine) silver(I) coordination complexes **4**–**9** (Figure 6). This symmetry disruption is a good indication for the Ag^+^ uptake by the bis(tetrazine) ligand **3** in solution. The coordination mode in solution could be derived from the single-crystal structure of **4** obtained by XRD (see above section). The complete signal attribution is reported in Table 1, including the *J* spin-spin coupling constants (SSCCs).

**Figure 7:**
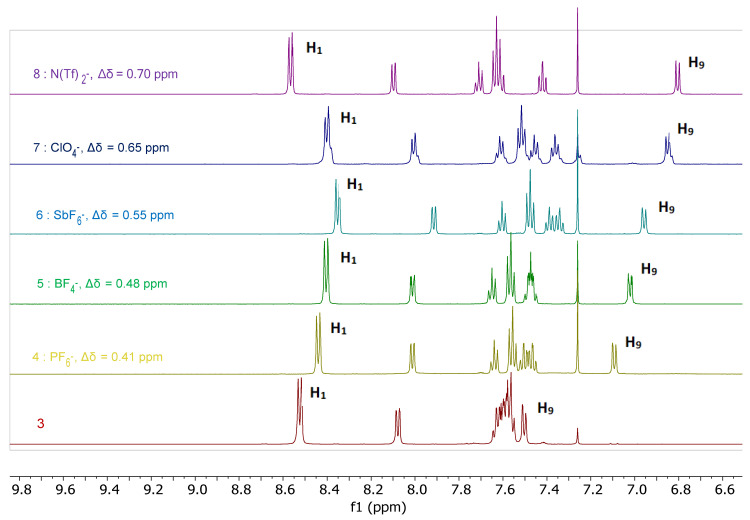
Comparative plot of the ^1^H-NMR (500 MHz, 298 K) spectra of the bis(tetrazine) **3** (bottom) and of the silver(I) complexes **4**–**8** recorded in CDCl_3_ (7.25 ppm).

As shown in Figure 7 for the complexes **4**–**8**, the H_9_ proton resonance is clearly the most affected one by the coordination, as it moves to stronger fields, with chemical shift changes of Δ*δ* = 0.41 (**4**, PF_6_^–^), 0.48 (**5**, BF_4_^–^), 0.55 (**6**, SbF_6_^–^), 0.65 (**7**, ClO_4_^–^), and 0.70 (**8**, NTf_2_^–^) ppm. In addition to the strong effect of silver coordination to the *o*-C–H protons, the nature of the counter anion also has a significant impact on the shift in a given solvent that is consistent with a strong ion-pairing in solution.

#### 2.3.2. Mass Spectrometry

The (+)-ESI-MS spectra of Ag(I)–bis(tetrazine) complexes **4**–**8** in CH_3_CN exhibited peaks at *m/z* = 573 a.m.u., with the appropriate silver isotopic distribution pattern for [Ag(**3**)]^+^. The peak at *m*/*z* = 1041 a.m.u. suggested the formation in the gas phase of the recombined cluster ion [Ag(**3**)_2_]^+^. Another cluster ion recombination corresponding to [Ag_2_(**3**)_2_(X)]^+^ was observed at *m*/*z* = 1291 (X = PF_6_^–^), 1381 (X = SbF_6_^–^), 1233 (BF_4_^–^) a.m.u., respectively (Figure S2, AgPF_6_ case). However, Ag^+^ being a very labile cation, it is difficult to conclude whether or not these ML_2_ and M_2_L_2_ species exist in solution, or if they might be formed in the MS gas phase by a rapid displacement of the equilibria during the drying of the spray. No evidence for any higher nuclearity species (oligomers) could be gained by ESI-MS.

#### 2.3.3. UV-Vis Absorption Spectroscopy

**Figure 8:**
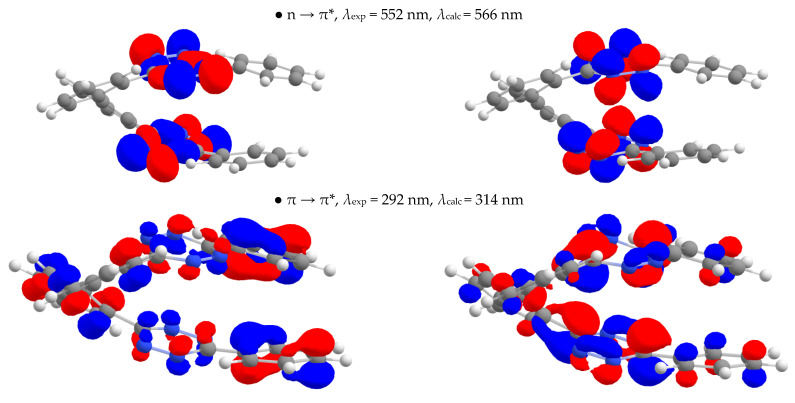
Natural transition orbitals for the main UV-visible absorption bands of **3** in CH_2_Cl_2_. Atom color code: carbon in grey, nitrogen in blue, hydrogen in white. Red surfaces correspond to positive values of the orbitals, while blue correspond to negative values.

The UV-Vis absorption spectrum in CH_2_Cl_2_ at 298 K of the monomeric 3,6-diphenyl-1,2,4,5-tetrazine **1** displays two absorption bands centered at 299 (*ε* = 33,542 M^−1^ cm^−1^) and 551 nm (*ε* = 465 M^−1^ cm^−1^) (Figure S3) [7]. The spectrum of the bis(tetrazine) **3** displays a similar profile in solution. For example, in CH_2_Cl_2_, two absorption bands at 293 (*ε* = 54260 M^−1^ cm^−1^) and 552 nm (*ε* = 820 M^−1^ cm^−1^) were observed (Figure S3 and Table S1). TD-DFT calculations unambiguously confirmed that the most intense UV band is due to an electronic *π*–π* transition, while the less intense one at 552 nm can be assigned to an *n*–π* transition with a HOMO → LUMO character (Figure 8) [21]. A similar spectral signature was found for **3** in CH_3_CN with *λ*_max_ = 289 nm (*ε* = 50,000 M^−1^ cm^−1^) and *λ*_max_ = 548 nm (*ε* = 830 M^–1^ cm^–1^).

The UV-Vis diffuse reflectance spectra of microcrystalline compounds diluted in dry BaSO_4_ were acquired with a “praying mantis” (Figure S4). The spectral shape of a solid sample of **3** (Figure S4) resembles that found in CH_2_Cl_2_ solution, albeit both band maxima *λ*_max_ = 301 and 568 nm displayed a bathochromic shift with respect to solution data, while the UV *π*–π* band is flanked at low energy by a broad shoulder (*λ*_sh_ ca. 390 nm). The latter is assigned to an *n*–π* transition, with a HOMO → LUMO+2 character. The amplitude of the bathochromic shift of both UV and vis bands is the largest in methanol, with that is defined as Δ*λ* = *λ*_solid_ – *λ*_solution_ = 14 and 26 nm, respectively (Table S1).

The UV-Vis spectra of complexes **4**–**8** were collected likewise in solution and in the solid state. In solution, the complexes **4**–**8** absorb in the same wavelength region as the bis(tetrazine) **3**. A weak bathochromic shift is observed for the band around 292 nm, and a hypsochromic shift for the band around 552 nm, relative to **3**, although the displacement does not exceed 4 nm (Figure S5, Table S2). In the solid state, both electronic transitions occur at lower energy with respect to the parent compound **3**, which translates into 10 and 6 nm shifts of the UV and vis bands, respectively. Moreover, the 390 nm shoulder observed in the diffuse reflectance spectrum of **3** undergoes likewise a red shift upon silver binding (Figure S6, Table S3).

#### 2.3.4. Electrochemical Properties

**Figure 9:**
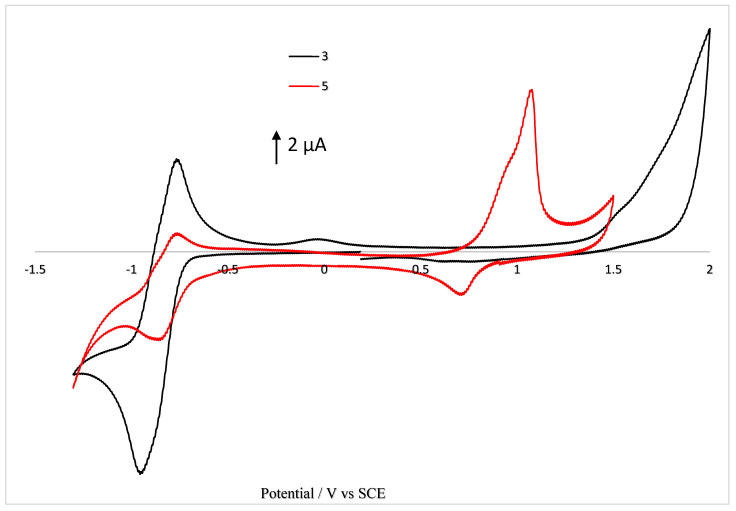
Cyclic voltammograms of **3** (black line) and complex **5** (red line) in CH_2_Cl_2_ containing 0.1 M TEABF_4_. Concentrations: 10^−3^ M; WE: platinum *Ø* = 2 mm, *ν* = 100 mV s^−1^.

Cyclic voltammetry (CV) was employed to study the electrochemical behavior of 3,6-diphenyl-1,2,4,5-tetrazine **1**, the bis(tetrazine) **3** and the complex {[Ag(**3**)][BF_4_]}_∞_ (**5**). The CV experiments were carried out in CH_2_Cl_2_ containing 0.1 M tetra(*n*-ethyl)ammonium tetrafluoroborate (TEABF_4_), see Figure 9, Figure S7, and Table S4, as supporting electrolyte. The voltammogram of **1** displays a single reversible reduction wave (*E*_1/2_ = −0.85 V/SCE) corresponding to the formation of the tetrazine radical anion [22,23], whereas bis(tetrazine) **3** undergoes two successive one-electron reductions at *E*_1/2_ = −0.83 and −0.93 V/SCE. According to theoretical approaches [24], molecules undergoing two successive reversible one-electron transfers centered on chemically equivalent, and fully independent redox sites (such as, for example, bis(ferrocenyl)porphyrins)[25] give rise to a 35.6 mV shift between each successive formal half-wave potentials. The resulting CV curve for each one-electron transfer should display the same 60 mV peak-to-peak separation [24,26]. Herein, for **3,** the potential gap between the successive one-electron reduction reactions is equal to 100 mV. This feature indicates that the redox sites, albeit chemically equivalent, are clearly not electronically independent from each other. This is attributed to a significant electronic communication that occurs between the two monomeric tetrazine moieties. The exact pathway for this electronic communication still remains unclear because disruption of aromaticity is expected to be rather significant when both heteroaromatic rings depart from a stacked, parallel arrangement, as observed in the solid state upon complex formation. The Ag(I) complex **5** is irreversibly reduced at *E*_pc_ = 0.70 V/SCE. At this potential, silver(I) is reduced into Ag(0), which plates out at the electrode surface. The second reduction peak at *E*_pc_ ca. −0.84 V/SCE is close to the value found for **3**, which confirms the dissociation of the Ag(I) complex. On the backward scan, a clear anodic stripping peak is observed at *E*_pa_ = 1.08 V/SCE for the dissolution of silver metal that was deposited on the electrode surface during the forward scan (Figure 7).

### 2.4. Host-Guest Behavior of Tweezer **3** with Silver Salts

The coordination of the cation Ag^+^ to tweezer **3** was confirmed both in the solid state and in solution. Considering the strong ion-pairing effect revealed by NMR spectroscopy for complexes **4**–**8** and the detection by mass spectrometry of dinuclear silver species, we investigated by ^1^H-NMR the speciation of Ag^+^ in the presence of tweezer **3**; this in order to establish the stoichiometry of the formed complexes and their stability. We selected a binary solvent mixture (CDCl_3_/CD_3_OD 2:1 *v/v*) for solubility reasons, as it enabled us to dissolve the silver salt precursor in a fairly large concentration range (2.5–34.9 mM), but also the tweezer **3** (in 25.6 mM), and the resulting coordination complexes.

**Figure 10:**
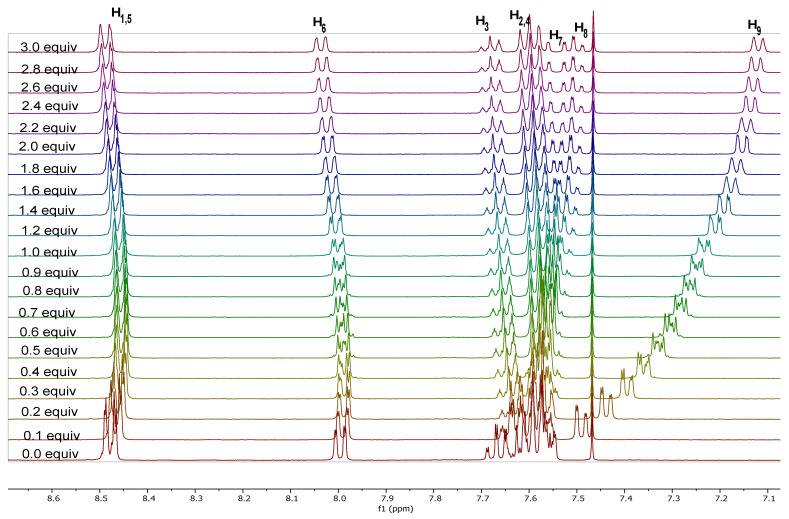
^1^H-NMR titration (400 MHz) of the bis(tetrazine) **3** ([3]_0_ = 2.56 × 10^–2^ M, *V*_0_ = 0.5 mL) with an AgNTf_2_ solution ([AgNTf_2_] = 6.44 × 10^−2^ M) in CDCl_3_/CD_3_OD 2:1 *v/v* at 298 K.

Upon successive additions of an AgX salt solution to a solution of **3**, all ^1^H-NMR resonances undergo progressive shifts, suggesting a fast exchange between the free and complexed Tz at the typical NMR time-scale (Figure 10, Table S5, AgNTf_2_ case). As previously mentioned, H_9_ protons, which are located in *α*-position with respect to the central C–C bond connecting the two phenyl-tetrazine moieties, experienced the largest up-field shift during the titration until reaching a limiting chemical shift value after addition of 3.0 equivalents of Ag^+^. Accordingly, we selected that particular signal to monitor the metal uptake process. Notably, solution equilibrium studies were restricted to the sole AgNTf_2_/**3** system for which no precipitation was observed up to the addition of 3.0 equivalents of Ag^+^. Indeed, the occurrence of precipitates upon addition of 0.6 to 1.0 equivalent of the various silver salts AgPF_6_, AgBF_4_, AgSbF_6_, AgClO_4_, and Ag(OTf) hampered a quantitative investigation. The formation of precipitates that turned out to be insoluble in all common solvents suggests that the soluble molecular complexes formed at the early stage of the titration progressively evolved into oligomeric or polymeric species. This is a common feature in supramolecular chemistry for labile host-guest assemblies of weak stability [27,28,29,30,31,32].

**Figure 11:**
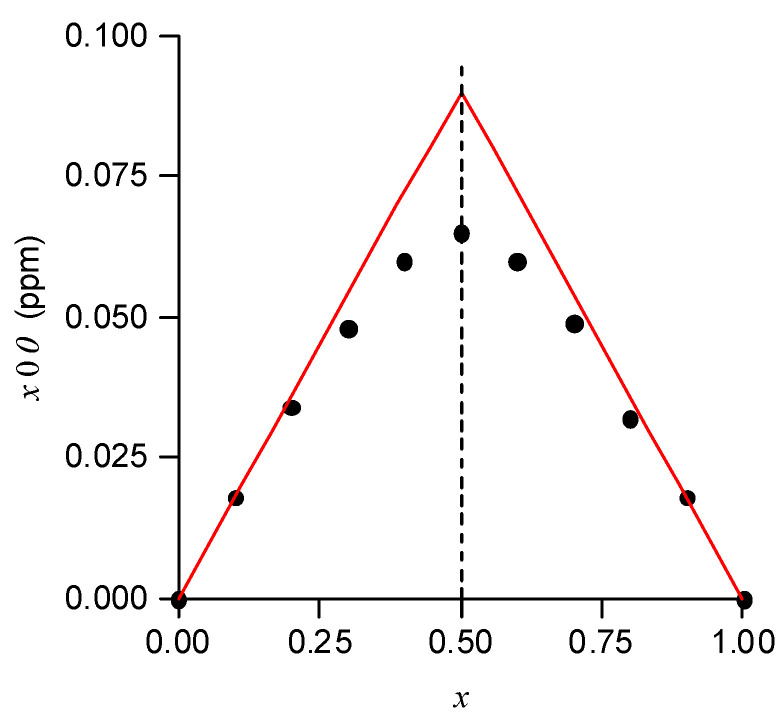
Job plot corresponding to a ^1^H-NMR titration (400 MHz) of **3** with AgNTf_2_ ([Ag]_tot_ + [3]_tot_ = 8.6 × 10^−3^ M) in CDCl_3_/CD_3_OD 2:1 *v/v* at 298 K. Δ*δ* is the coordination induced chemical shift variation of protons H_9_ and *x* the molar fraction of **3** (*x* = [3]/ [Ag]_tot_ + [3]_tot_). Red lines correspond to the asymptotes that intersect at *x* = 0.5, suggesting the formation of [Ag(**3**)]^+^ in solution.

To obtain a first insight into the stoichiometry of the formed species, an additional titration following the continuous variation method was performed [33,34,35,36,37,38]. According to the Job plot shown in Figure 11 [27,28,29,30,31,32], the maximum of the variation of the chemical shift of H_9_ protons is reached for *x* = [3]_tot_/([Ag]_tot_ + [3]_tot_) = 0.5. This suggests a 1:1 Ag/**3** stoichiometry for the prevailing complex in solution. However, the formation of additional species (for example, the 1:2 or 2:1 complexes) possibly present in lower amounts is often difficult to detect by this method, mainly because the position of the maximum and the overall shape of the graph are only slightly affected in those cases [39,40,41].

**Figure 12:**
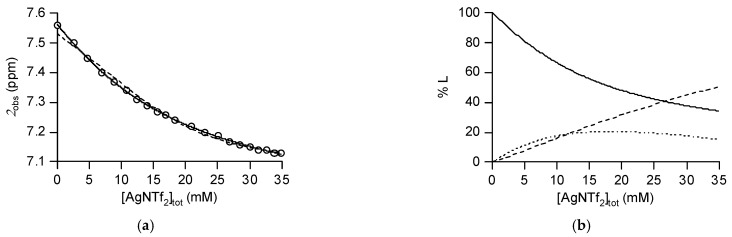
(**a**) ^1^H-NMR chemical shift variation of protons H_9_ of the bis(tetrazine) **3** ([3]_0_ = 2.56 × 10^−2^ M, *V*_0_ = 0.5 mL) as a function of the total silver salt concentration in CDCl_3_/CD_3_OD 2:1 *v/v* at 298 K. The dashed and solid lines correspond to the best-fit curve according to Equation (2) for the single ([Ag(**3**)]^+^) and two-species ([Ag(**3**)]^+^ and [Ag(**3**)_2_]^+^) models, respectively. (**b**) Distribution diagram for the AgNTf_2_/bis(tetrazine) **3** system corresponding to the titration conditions: [Ag]_tot_ = (0−3.49) × 10^−2^ M, [3]_tot_ = (2.56−1.16) × 10^−2^ M, *V* = 0.5–1.1 mL. Solid line: **3**, dashed line: [Ag(**3**)]^+^, and dotted line: [Ag(**3**)_2_]^+^.

Therefore, the chemical shifts of protons H_9_ (*δ*_obs_), which were determined upon incremental addition of 20 aliquots of an AgNTf_2_ solution (up to 3 equivalents) to a 25.6 mM solution of **3** in CDCl_3_/CD_3_OD 2:1 *v/v* (Figure 10), were adjusted by nonlinear least-squares with the HypNMR 2008 program (Figure 12a) [42] for the set of complexation equilibria we propose below (Equation (1)).

(1) $$mM+lL\;⇋\;M_{m}L_{l}\;\;\;\;\;\;\;\;\;\;\;\beta_{ml}=\frac{\left[M_{m}L_{l}\right]}{{\left[M\right]}^{m}{\left[L\right]}^{l}}$$

Since the binding/dissociation kinetics is much faster than the NMR observation time, the experimental chemical shift can be expressed according to Equation (2), where *α_ml_*, *δ_ml_*, and *β_ml_* stand for the molar fraction, the intrinsic chemical shift, and the global stability constant of the M*_m_*L*_l_* species, respectively, while *m* and *l* correspond to the stoichiometric coefficients (by convention, *β*_10_ = *β*_01_ = 1). The HypNMR software iteratively refines the equilibrium constants *β_ml_* for a given chemical model and calculates the intrinsic chemical shift *δ_ml_* for each species incorporating one or more molecules of ligand by solving at each iteration and for each analyzed sample the mass-balance equations.

(2) $$\delta_{\mathrm{obs}}=\sum \alpha_{ml}\delta_{ml}=\sum \frac{l\beta_{ml}{\left[M\right]}^{m}{\left[L\right]}^{l}}{\sum l\beta_{ml}{\left[M\right]}^{m}{\left[L\right]}^{l}}\;\delta_{ml}$$

A fairly satisfactory fit (dashed line in Figure 12a) with a root mean square deviation (RMSD) of 0.012 ppm was obtained by adjusting the data with a single equilibrium involving the 1:1 complex (*m* = *l* = 1) as suggested by the Job plot. The value of the refined stability constant is log *β*_11_ = 2.4 ± 0.1. The chemical shift calculated for **3** (*δ*_01_ = 7.530 ± 0.006 ppm) deviates by 0.03 ppm from the experimental value measured for a pure sample (*δ*_01_ = 7.560 ppm), while that refined for [Ag(**3**)]^+^ is *δ*_11_ = 7.064 ± 0.006 ppm. Accordingly, the 1:1 complex was formed only at 70% at the end of the titration, which corresponded to the addition of 3 equivalents of silver salt.

A second equilibrium was introduced in the chemical model that improve the goodness of fit. The refinement diverged when the binuclear [Ag_2_(**3**)]^2+^ complex was considered. However, a significant improvement (RMSD = 0.003 ppm) could be achieved by including the [Ag(**3**)_2_]^+^ complex in the model (solid line in Figure 12a). The latter species fully compensates for the lack of fit (up to 0.03 ppm) seen for all data points corresponding to metal-over-ligand molar concentration ratios below 1 equivalent with the one-equilibrium model. The optimization procedure converged for the following parameters: log *β*_11_ = 1.72 ± 0.01, log *β*_12_ = 3.30 ± 0.01, *δ*_01_ = 7.561 ± 0.003 ppm, *δ*_11_ = 6.875 ± 0.003 ppm, and *δ*_12_ = 6.981 ± 0.003 ppm. A Job plot was simulated for this chemical model and the same concentrations as those used experimentally. The maximum of the concave curve was found at *x* = 0.55 instead of the theoretical value of 0.5 expected for the sole formation of a 1:1 complex. As already mentioned, the slight shape dissymmetry introduced by the [Ag(**3**)_2_]^+^ species makes its detection by the continuous variation method difficult [40,41]. This two-equilibria model is also supported by the intrinsic chemical shift refined for bis(tetrazine) **3**, which perfectly matches with the experimental value. Moreover, the fact that the optimized *δ*_12_ value (6.98 ppm) for [Ag(**3**)_2_]^+^ is ranging in-between those obtained for the free tweezer **3** (7.56 ppm) and the corresponding 1:1 silver complex (6.88 ppm) provides further support. Accordingly, an intermediate conformation of both tweezer molecules in [Ag(**3**)_2_]^+^, located somewhere between the closed form of **3** stabilized by [C_2_N_4_]···[C_2_N_4_] intramolecular *π*–*π* interactions [3], and the more open structure found in [Ag(**3**)]^+^, might be postulated.

The low value computed for the first (log *β*_11_ = 1.72 ± 0.01) and second (log *β*_12_ = 3.30 ± 0.01) global equilibrium constants clearly indicates that the bis(tetrazine) **3** forms extremely weak complexes with the soft Ag^+^ cation in the selected binary solvent mixture (CDCl_3_/CD_3_OD 2:1 *v/v*). The low affinity of **3** is best illustrated by the distribution diagram corresponding to the titration conditions (Figure 12b). After addition of 1 equivalent of metal, only 50% of **3** has reacted with Ag^+^ to form 29% of [Ag(**3**)]^+^ and nearly 21% of [Ag(**3**)_2_]^+^. For 3 equivalents of silver, the fraction of free-bis(tetrazine) drops to 34%, while the proportion of [Ag(**3**)]^+^ and [Ag(**3**)_2_]^+^ accounts for 50% and 15%, respectively. The stepwise stability constant of [Ag(**3**)_2_]^+^ (*K*_12_ = *β*_12_/*β*_11_) associated to equilibrium (3) equals 1.58 ± 0.04 logarithmic units.

(3) $${\left[\mathrm{Ag}\left(3\right)\right]}^{+}+3\;⇋\;{\left[\mathrm{Ag}{\left(3\right)}_{2}\right]}^{+}\;\;\;\;\;\;\;\;K_{12}=\frac{\left[\mathrm{Ag}{\left(3\right)}_{2}\right]}{\left[\mathrm{Ag}\left(3\right)\right]\left[3\right]}$$

Although such low values of equilibrium constants have to be interpreted with some caution, the difference Δ_12_ = log *K*_11_ − log *K*_12_ = 0.14 ± 0.04 is lower than the statistical factor (log 4 ≈ 0.60) expected for a stepwise ligand uptake by a bis-coordinated silver center [43], as it was found in the crystal structure of {[Ag(**3**)][SbF_6_]·CHCl_3_}_∞_. Therefore, the cooperative formation of [Ag(**3**)_2_]^+^ in the early stages of the titration, when the ligand is still in excess over Ag^+^, rules out strong steric interactions between the molecules of bis(tetrazine), as their occurrence would contribute to an increase in Δ_12_. The analysis of non-covalent interactions in [Ag(**3**)_2_]^+^ also indicates that the dispersion interaction between the two ligands may compensate for the steric repulsion (Figure S8). It is, however, likely that the counter anion contributes to stabilizing the species in solution by an ion-pairing mechanism (electrostatic outer-sphere interactions) in weakly polar media, as suggested by the different titration profiles (chemical shift variations) for the H_9,9’_ proton pair that could be observed for the other considered silver salts.

## 3. Materials and Methods

### 3.1. General Synthetic Conditions

The bis(tetrazine) **3** was prepared following our previously reported procedure [3]. All reagents and precursors (AgPF_6_, AgBF_4_, AgSbF_6_, AgClO_4_, AgNTf_2_, and Ag(OTf)) were purchased from commercial suppliers and used without purifications. All reactions were performed under an atmosphere of dry argon in Schlenk tubes, glass vials, or in a microwave reaction vessel. Microwave heating was carried out using a CEM Discover microwave reactor equipped with an infrared temperature sensor. The microwave-assisted reactions were run in closed vessels under magnetic stirring. All solvents were dried and deoxygenated prior to use.

### 3.2. Physical Methods

All NMR spectra (^1^H, ^13^C, ^19^F, and ^31^P) were recorded on Bruker Avance III spectrometers operating at 400, 500, or 600 MHz in solution with the deuterated solvent as the lock and the residual solvent or TMS as the internal reference. Chemical shifts are reported in ppm relative to deuterated solvent, and coupling constants *J* are given in Hz. High-resolution Mass Spectra (HRMS) were obtained on a Thermo LTQ-Orbitrap XL with an ESI source. Flash chromatography was performed on silica gel (230-400 mesh).

### 3.3. Titration Procedures

^1^H-NMR titrations were performed using deuterated solvents. All ligand and metal-salt solutions used for titrations were prepared in volumetric flasks by dissolving weighted amounts (±0.1 mg) of each reagent in the mixed solvent CDCl_3_/CD_3_OH 2:1 *v/v*. Additions were made with 100 μL Hamilton micro-syringes.

### 3.4. Determination of the Stoichiometry of the Complexes

The stoichiometry of the complexes formed between the bis(tetrazine) and the metal salt was determined by applying the continuous variation method, also known as the Job method [27], to a ^1^H-NMR titration. Two equimolar stock solutions of tetrazine ([**3**]_tot_ = 8.6 mM) and of the metal salt ([Ag]_tot_ = 8.6 mM) in the binary solvent mixture were mixed in different proportions varying from 0 to 100% in 10% steps, in order to keep the total concentration corresponding to the sum of the two-species (tetrazine + metal salt) constant at 8.6 mM. The Job plot is a graphical representation of the *x*Δδ product as a function of *x* = [3]_tot_/([Ag]_tot_ + [3]_tot_), where Δ*δ* = *δ*_free ligand_ − *δ*_obs_.

### 3.5. Determination of the Association Constants

To determine the association constants between the tetrazine **3** and Ag^+^, a second ^1^H-NMR titration was carried out by incremental addition of aliquots of a silver salt stock solution to a solution of **3** contained in a 5-mm NMR tube. The initial volume was 0.5 mL. After each metal addition, the sample was mixed by vigorous shaking, and the ^1^H-NMR spectrum was recorded on a 400 MHz spectrometer at a fixed temperature of 298 K. Each spectrum was carefully calibrated using the CD_3_OD signal as reference (*δ* = 3.31 ppm). The variations of the chemical shift of protons H_9,9’_ as a function of the total ligand and metal concentrations were processed with the nonlinear least-squares program HypNMR 2008 (Protonic Software) [42]. Since a unitary weighing scheme (*ω_i_* = 1) was selected, the returned RMSD of the fit can be directly compared to the measurement precision of chemical shifts (typically, *σ* ≈ 0.002 ppm). Hence, in the absence of systematic errors and for a correct chemical model (i.e., randomly distributed residuals), the expectation value of the RMSD should equal *σ*.

### 3.6. UV-Visible Absorption and Diffuse Reflectance Spectrophotometry

UV-Vis absorption spectra in solution were recorded at room temperature with a Cary 50 (Varian) spectrophotometer using a quartz cell (Hellma) of 1 cm optical path length. Concentrations were about 10^–5^ M (between 230 and 430 nm) or 10^–3^ M (between 430 and 630 nm). Diffuse reflectance spectra of pure microcrystalline or amorphous compounds diluted in dry barium sulfate (avocado, precipitated, >99%) were acquired between 200 and 800 nm on a CARY 5000 (Agilent) UV-Vis-NIR spectrophotometer fitted with a Praying Mantis^TM^ accessory (Harrick). The baseline was recorded with BaSO_4_. Corrected reflectance data were converted to *f*(*R*) values using the Kubelka–Munk function expressed as *f*(*R*) = (1 – *R*)^2^/2*R*, where *R* stands for the reflectance.

### 3.7. Cyclic Voltammetry Measurements

All manipulations were performed using Schlenk techniques in an oxygen-free atmosphere of dry argon at room temperature (*θ* = 20 ± 3 °C). The supporting electrolyte (TEABF_4_) was degassed under vacuum before use and then dissolved in CH_2_Cl_2_ to a concentration of 0.1 M. Voltammetric analyses were carried out in a standard three-electrode cell with an Autolab PGSTAT 302 N potentiostat, connected to an interfaced computer and controlled by the Electrochemistry Nova software (v. 1.11). The reference electrode was a KCl saturated calomel electrode (SCE) separated from the analyzed solution by a sintered-glass salt bridge filled with the background electrolyte solution. The auxiliary electrode was a platinum foil separated from the analyzed solution by a sintered-glass salt bridge filled with the background electrolyte solution. For all voltammetric measurements, the working electrode was a platinum (GC) electrode (Ø = 2 mm) that was polished with a diamond suspension. In these conditions (0.1 M TEABF_4_ in CH_2_Cl_2_), the formal half-wave potential for the ferrocenium/ferrocene couple was +0.46 V vs. SCE.

### 3.8. Computational Details

Quantum mechanics calculations were performed with the Gaussian16 software package [44]. Energy and forces were computed by density functional theory with the hybrid B3PW91 exchange-correlation functional. In addition, dispersion effects have been considered using the D3 dispersion correction suggested by Grimme et al. with Becke-Johnson damping [45]. This computational level will be denoted by B3PW91-D3. The solvent effects were modeled by means of the continuum model as implemented in Gaussian. Geometries were optimized and characterized with the aug-cc-pVTZ basis set [46]. The non-covalent interactions were visualized with the Independent Gradient Model [47,48] that can be seen as an extension of the NCI method of Yang et al. [49]. The UV-visible transitions were computed using the B3PW91-D3 functional with the 6-31+G(d,p) basis sets. The orbital transitions of selected excited states were characterized by using the natural transition orbital (NTO) method with an isovalue of 0.04 au [50].

## 4. Conclusions

New supramolecular polymeric architectures were achieved by self-assembly of silver(I) cations with the bis(tetrazine) **3**. The intrinsic tweezer-like flexibility of **3** has been confirmed in self-assembly processes with transition metal ions: Ag^I^, Cu^I^, and Pd^II^. The resulting discrete coordination polymers were identified by the combination of physicochemical analyses in solution and XRD identification in the solid state. The supramolecular polymeric structure of the crystalline complexes {[Ag(**3**)][PF_6_]}_∞_ and {[Ag(**3**)][SbF_6_]}_∞_ were fully described, and although we could not further confirm by X-ray diffraction the structures of the other metal complexes, the combination of analytical characterization methods clearly indicated similar structures in solution and in the solid state for all the obtained host-guest silver compounds. The physical measurements (NMR, XRD, UV, and electrochemistry) evidenced weak interactions of the ligand with the metal cation, as well as within the polymeric backbone. These interactions were confirmed by the variations in chemical shift values, absorption maxima, electrochemical reduction potentials, as well as low values of association constants obtained by host-guest titrations. These trends were confirmed by theoretical calculations, which revealed weak non-covalent interactions in the new coordination polymers crystallized from the bis(tetrazine) **3**.

## Figures and Tables

**Table 1:** ^1^H-NMR (400 MHz, 298 K) spectra of complex **4** in CD_2_Cl_2._

Attribution	Chemical Shift (ppm in CD_2_Cl_2_)	SSCCs (Hz)
H_1,5_ (dd, 4H)	8.41	^3^*J*_1,2_ = 7.4 and ^4^*J*_1,3_ = 1.4
H_6_ (dd, 2H)	7.97	^3^*J*_6,7_ = 7.6 and ^4^*J*_6,8_ = 1.4
H_3_ (tt, 2H)	7.66	^3^*J*_3,2(4)_ = 7.4 and ^4^*J*_3,1(5)_ = 1.3
H_2,4_ (p-t, 4H)	7.56	^3^*J*_2,1_ = ^3^*J*_2,3_ = 7.4
H_7_ (ddd, 2H)	7.50	^3^*J*_7,6_ = ^3^*J*_7,8_ = 7.6 and ^4^*J*_7,9_ = 1.3
H_8_ (ddd, 2H)	7.45	^3^*J*_8,7_ = ^3^*J*_8,9_ = 7.6 and ^4^*J*_8,6_ = 1.5
H_9_ (dd, 2H)	7.08	^3^*J*_9,8_ = 7.6 and ^4^*J*_9,7_ = 1.3

## Data Availability

Computational data can be obtained by contacting P.F.-L.

## Supplementary Materials

Crystallographic data (CIF). CCDC 2068275 (**4**) and 2068274 (**6**) contain the supplementary crystallographic data for this paper. These data can be obtained free of charge from The Cambridge Crystallographic Data Centre.
