# Supplementary material for: Coordination Chemistry of a Bis(Tetrazine) Tweezer: A Case of Host-Guest Behavior with Silver Salts
Source: Molecules. 2021 May 5;26(9):2705. doi: 10.3390/molecules26092705 (PMC8124956; doi:10.3390/molecules26092705)
Supplement: Supplementary file 1 [file molecules-26-02705-s001.zip › molecules-1195645-supplementary.pdf]

Coordination Chemistry of A Bis(Tetrazine) Tweezer: A Case of Host-Guest Behavior With Silver Salts

C. D. Mboyi, O. Amamou, P. Fleurat-Lessard, J. Roger, H. Cattey, C. H. Devillers, M. Meyer, T. Boubaker and J.-C. Hierso

---

CONTENT

Solvent effect monitored by ^{1}H NMR… **S2**

Mass Spectrum of silver complex 4 from tweezer 3… **S2**

UV–vis absorption spectroscopy… **S3-5**

Electroanalysis… **S6**

Association constant determination by ^{1}H NMR titration… **S6-7**

Visualization of dispersion interactions… **S7**

XRD data and structure… **S7-18**

Copper-catalyzed tweezer 3 synthesis… **S19**

Coordination polymers synthesis... **S19-24**

Copy of ^{1}H and ^{13}C NMR spectra… **S25-37**

Mass spectrum of copper and palladium complexes… **S38-39**

Solvent effect monitored by ^{1}H NMR

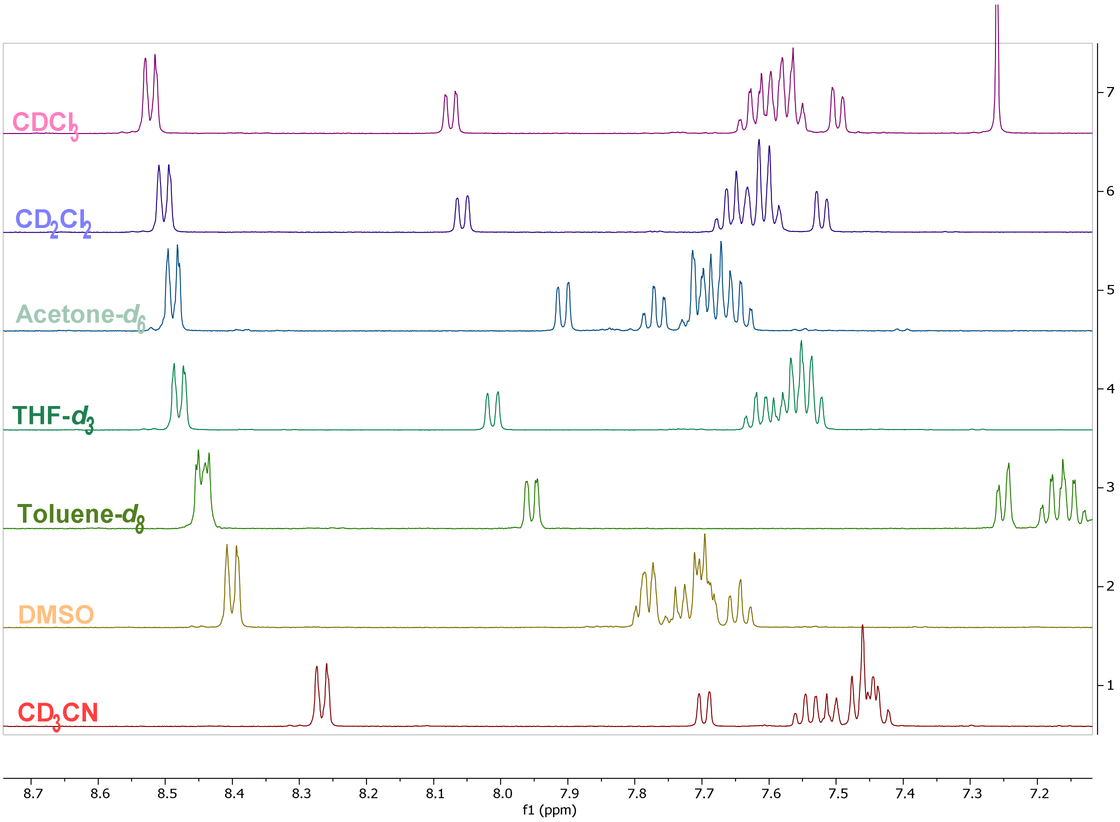

Figure S1. Evolution of the ^{1}H NMR (500 MHz) spectrum of **3** depending on the polarity of the solvent.

Mass Spectrum of silver complex 4 from tweezer 3

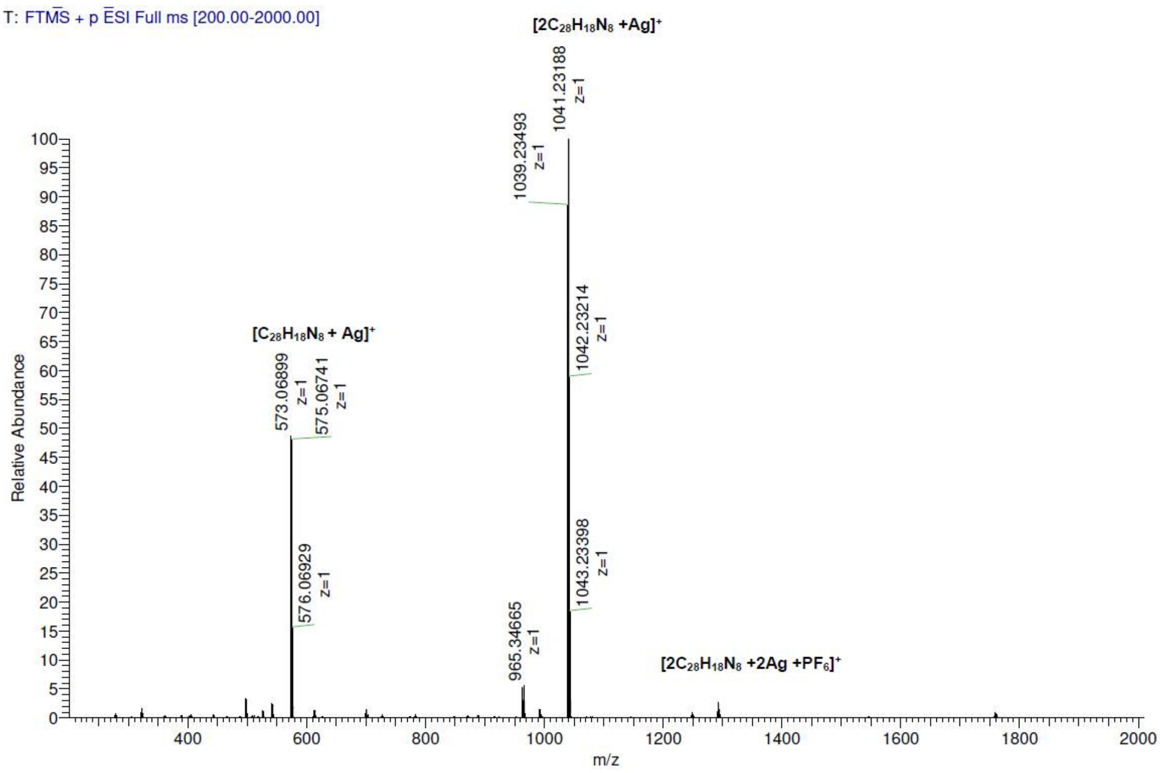

Figure S2. ESI-MS spectrum of Ag(**3**)[PF$_6$] (**4**) and related clusters [Ag(**3**)$_2$]$^+$ and [Ag$_2$(**3**)$_2$]$^+$.

UV–vis absorption spectroscopy

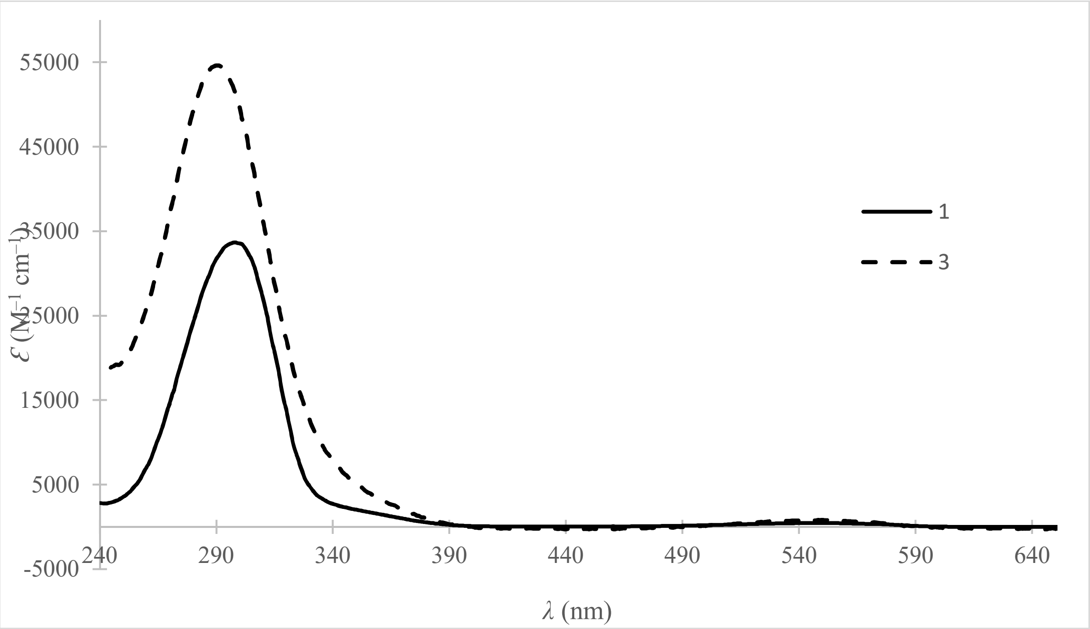

Figure S3. UV-Vis spectra of 3,6-diphenyl-1,2,4,5-tetrazine **1** (solid black line) and bridge-clamp bis(tetrazine) **3** (red dashed line) recorded in CH_2Cl_2 solution with a concentration *ca.* 10^{-5} M at room temperature.

Table S1. UV-vis study of crystalline **3** in solution and solid state.

Solvent or solid	λ_1 (nm)	λ_2 (nm)	ε_1 (M^{-1} cm^{-1})	λ_3 (nm)	ε_2 (M^{-1} cm^{-1})
**Toluene**	291	-	43682	558	1359
**$CHCl_3$**	292	-	54077	552	551
**CH_2Cl_2**	292	-	54258	552	819
**THF**	292	-	57000	550	1921
**$(CH_3)_2CO$**	290	-	>30000	545	781
**$(CH_3)_2SO$**	290	-	42474	553	1206
**CH_3CN**	289	-	49972	548	829
**Methanol**	287	-	19784	542	276
**3 in solid-state**	301	387	-	568	-

[a] UV-Vis spectra of crystalline **3** in solution recorded at room temperature in various solvents with a ≈ 10^{-5} M; reflectance spectra was obtained with microcrystalline of **3** diluted in dry $BaSO_4$.

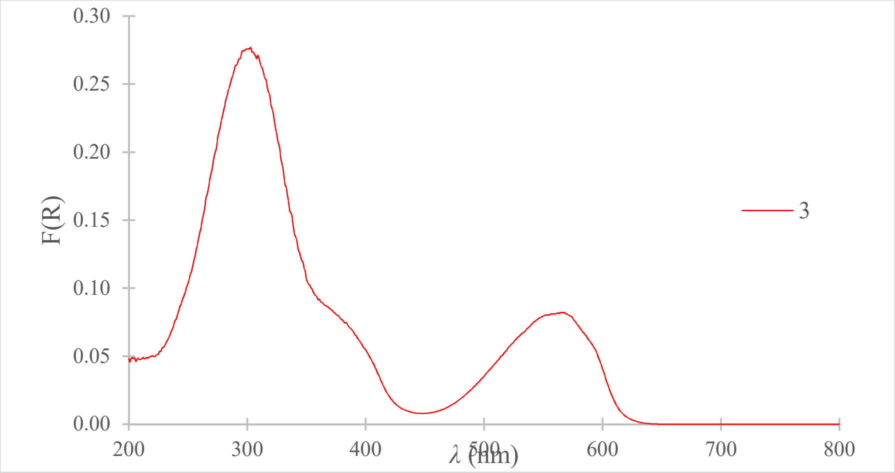

Figure S4. Diffuse reflectance spectra of microcrystalline **3** ligand diluted in dry barium sulfate ($BaSO_4$).

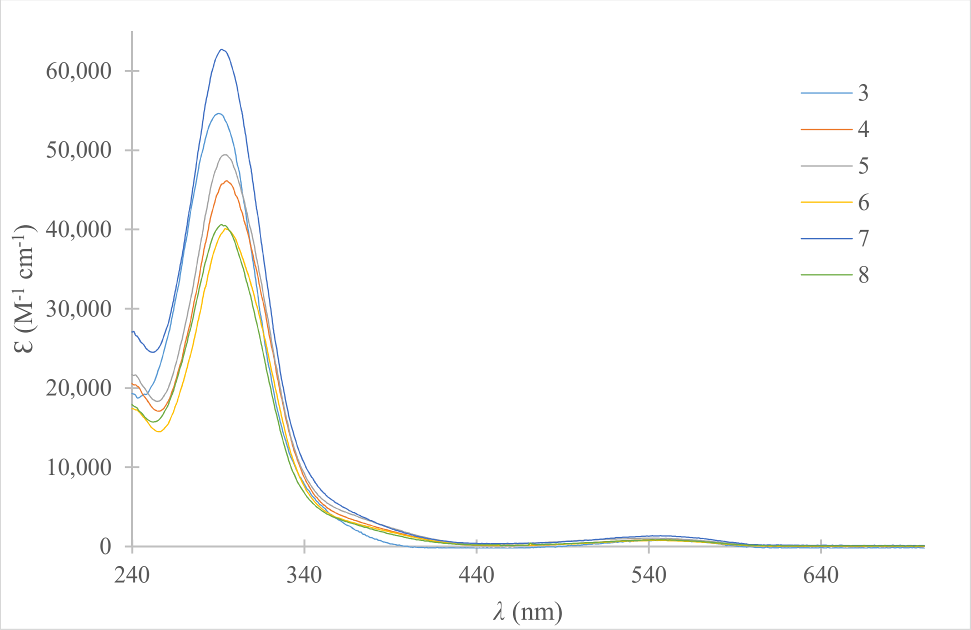

Figure S5. Molar absorption coefficient of compounds **3**–**8** in dichloromethane at about 8.0–9.0.10^{-5} M.

Table S2. Photophysical properties for compounds **3** ligand and complexes **4–8** in dichloromethane: Absorption wavelength (λ, nm), molar absorption coefficient ($\mathcal{E}$, M^{-1} cm^{-1}).[a]

**Compound**	**λ_1 (nm)**	$\mathcal{E}_1$ (M^{-1}.cm^{-1})	**λ_2 (nm)**	$\mathcal{E}_1$ (M^{-1}.cm^{-1})
**3**	291	54609	554	752
**4**	293	46000	553	862
**5**	295	49392	551	1048
**6**	294	39775	550	760
**7**	294	62515	555	1299
**8**	292	40612	550	838

[a] λ_{max} at the maximum of absorbance and the molar extinction coefficients are determined from solutions prepared at a concentration ranging from 10^{-5} to $5x10^{-5}$ M in CH_2Cl_2 at room temperature with 1 cm optical path in quartz cell.

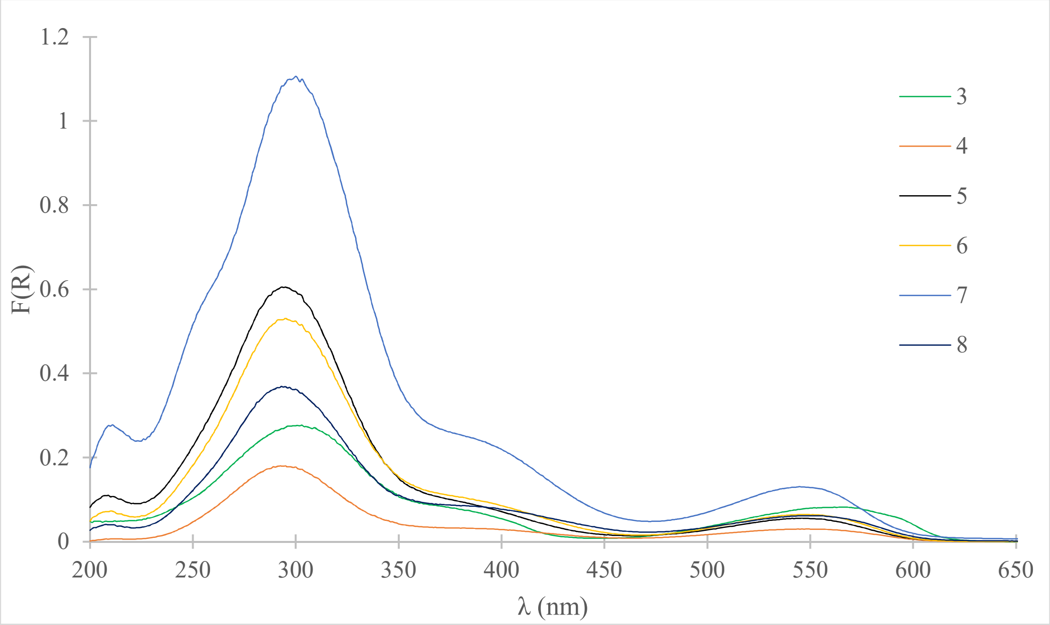

Figure S6: Diffuse reflectance spectra of **3 l**igand and complexes **4–8** diluted in dry barium sulfate ($BaSO_4$).

Table S3. Photophysical properties for **3** ligand and complexes **4–8** in solid state: Absorption wavelength (λ, nm) molar absorption coefficient ($\mathcal{E}$, $M^{-1}.cm^{-1}$).[a]

Compound	λ_1 (nm)	λ_2 (nm)	$\Delta\lambda^b$	λ_3 (nm)
**3**	301	375	–	560
**4**	295	393	18	550
**5**	294	386	11	547
**6**	296	390	15	550
**7**	300	390	15	547
**8**	294	394	19	552

[a] Diffuse reflectance spectra were acquired between 200 and 800 nm on a CARY 5000 (Agilent). UV–vis–NIR spectrophotometer fitted with a Praying MantisTM accessory (Harrick), the baseline being recorded on $BaSO_4$. Corrected reflectance data were converted to f(R) values using the Kubelka-Munk function expressed as $f(R) = (1 - R)^2/(2R)$, where R stands for the reflectance. [b] $\Delta\lambda = \lambda_{2(complex)} - \lambda_{2(free\ ligand\ 3)}$.

Electroanalysis

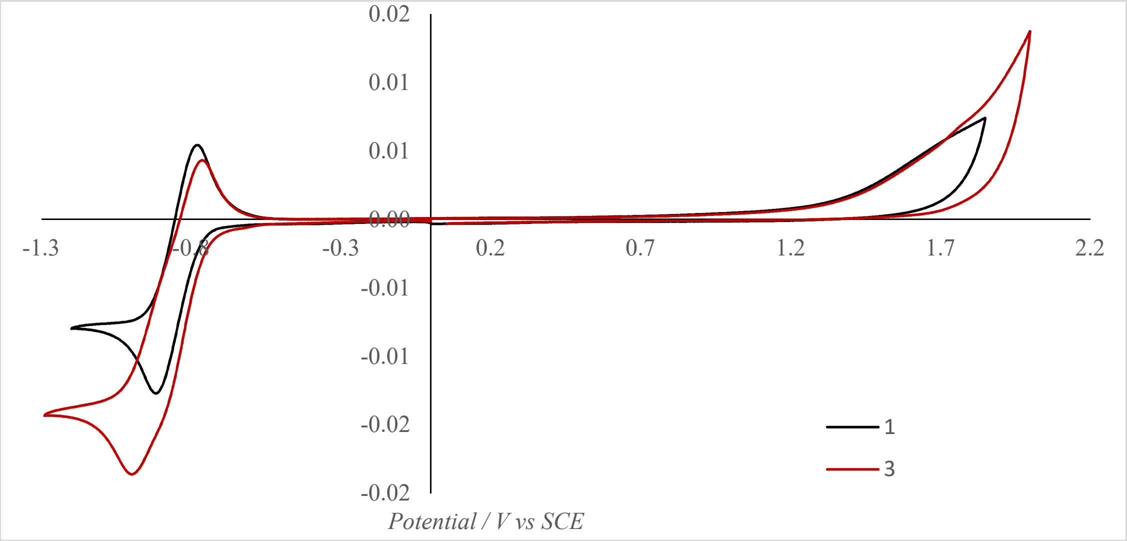

Figure S7. Cyclic voltammograms of 3,6-diphenyl-1,2,4,5-tetrazine **1** (black line) and **3** (red line) in CH_2Cl_2 0.1 M $TEABF_4$. Concentration: 10^{-3} M; WE: platinum Ø = 2 mm, v = 100 mV s^{-1}.

Table S4. Redox events in **1** and **3**.

Compound	$E_{2red}^{1/2}$/ V	$E_{1red}^{1/2}$/ V
**1**	-	-0.85
**3**	-0.93	-0.83

Association constant determination by ^{1}H NMR titration

Table S5: ^{1}H NMR (400 MHz, 298 K) titration of bis(tetrazine) **3** in $CDCl_3/CD_3OD$ (25.6x10^{-3} M 2:1 *v*/*v*) with $AgNTf_2$ in $CDCl_3/CD_3OD$ (1.28x10^{-2} mmol, 25.6 × 10^{-3} M 2:1 *v*/*v*)

Host (*H*) : **3**	Guest (*G*): $AgN(Tf)_2$
M_H [g.mol^{-1}] : 466.17	M_G [g.mol^{-1}] : 388.01
m_H [mg] : 6.0	m_G [mg] : 50.0
Solvent : $CDCl_3$+CD_3OD 2 : 1	Solvent : $CDCl_3$+CD_3OD 2 : 1
V_0 [mL] : 0.5	V_0 [mL] : 2.0
[**3**]$_0$ [mM] : 25.6	$[AgN(Tf)_2]_0$ [mM] : 64.4
T[°C] : 25, δ_0 [ppm] = 7.50	

An aliquot of 20 µL corresponds to 0.1 equivalent of $AgN(Tf)_2$ relative to the ligand **3**

V[mL]	[**3**] (M)	[AgN(Tf)$_2$] (M)	δH$_9$	$\Delta\delta$H$_9$
0.5	0.0256	0.0000	7.56	0
0.52	0.0246	0.0025	7.5	0.06
0.54	0.0237	0.0047	7.45	0.11
0.56	0.0229	0.0069	7.4	0.16
0.58	0.0221	0.0088	7.37	0.19
0.6	0.0213	0.0107	7.34	0.22
0.62	0.0206	0.0124	7.31	0.25
0.64	0.0200	0.0140	7.29	0.27
0.66	0.0194	0.0155	7.27	0.29
0.68	0.0188	0.0169	7.26	0.3
0.7	0.0183	0.0183	7.24	0.32
0.74	0.0173	0.0208	7.22	0.34
0.78	0.0164	0.0230	7.2	0.36
0.82	0.0156	0.0250	7.19	0.37
0.86	0.0149	0.0268	7.17	0.39
0.9	0.0142	0.0284	7.16	0.4
0.94	0.0136	0.0300	7.15	0.41
0.98	0.0131	0.0313	7.14	0.42
1.02	0.0125	0.0326	7.14	0.42
1.06	0.0121	0.0338	7.13	0.43
1.1	0.0116	0.0349	7.13	0.43

Visualization of dispersion interactions

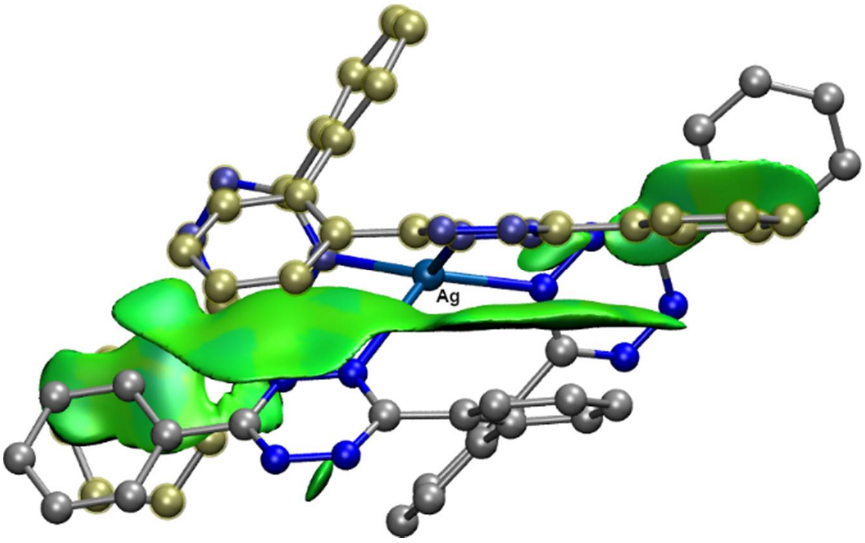

Figure S8. Visualization of dispersion interactions between the two bis-tetrazine ligands in [Ag(**3**)$_2$]$^+$.

XRD data and structure

Complex 4

Report created with ReportPlus

Crystal Data and Experimental

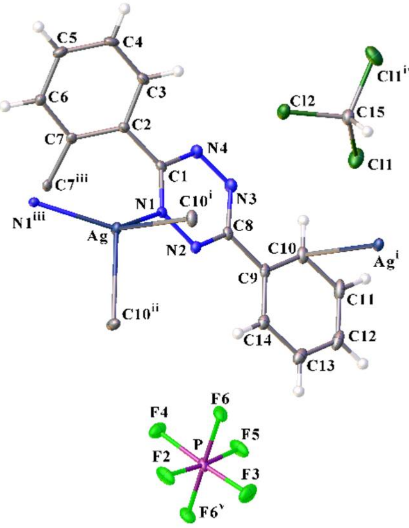

Figure S9: (i) 1-x,1-y,1-z, (ii) -1/2+x,+y,1-z, (iii) 1/2-x,1-y,+z, (iv) +x,1/2-y,1/2-z, (v) +x,3/2-y,1/2-z

Experimental. Single clear light red prism-shaped crystals of **Complex 4** were crystallized from dichloromethane by slow evaporation. A suitable crystal 0.27×0.23×0.22 mm^3 was selected and mounted on a mylar loop with oil on an Bruker D8 Venture triumph Mo diffractometer. The crystal was kept at a steady T = 100.0 K during data collection. The structure was solved with the **ShelXT** (Sheldrick, 2015) structure solution program using the Intrinsic Phasing solution method and by using **Olex2** (Dolomanov et al., 2009) as the graphical interface. The model was refined with version 2018/3 of **ShelXL** (Sheldrick, 2015) using Least Squares minimization.

Crystal Data. $C_{29}H_{19}AgCl_3F_6N_8P$, M_r = 838.71, orthorhombic, *Pnna* (No. 52), a = 14.6561(10) Å, b = 22.1843(16) Å, c = 9.3571(7) Å, $\alpha = \beta = \gamma$ = 90°, V = 3042.3(4) Å^3, T = 100.0 K, Z = 4, Z' = 0.5, μ(MoK$_\alpha$) = 1.054, 35124 reflections measured, 3500 unique (R_{int} = 0.0228) which were used in all calculations. The final wR_2 was 0.0588 (all data) and R_1 was 0.0249 (I > 2(I)).

**Compound**	**Complex 4**
**CCDC**	2068275
Formula	$C_{29}H_{19}AgCl_3N_8$, PF_6
$D_{calc.}$/ g cm^{-3}	1.831
μ/mm^{-1}	1.054
Formula Weight	838.71
Colour	clear light red
Shape	prism
Size/mm^3	0.27×0.23×0.22
T/K	100.0
Crystal System	orthorhombic
Space Group	*Pnna*
a/Å	14.6561(10)
b/Å	22.1843(16)
c/Å	9.3571(7)
α/°	90
β/°	90
γ/°	90
V/Å^3	3042.3(4)
Z	4
Z'	0.5
Wavelength/Å	0.710760
Radiation type	MoK$_\alpha$
Θ_{min}/°	2.583
Θ_{max}/°	27.510
Measured Refl.	35124
Independent Refl.	3500
Reflections with I > 2(I)	3187
R_{int}	0.0228
Parameters	241
Restraints	0
Largest Peak	0.920
Deepest Hole	-0.439
GooF	1.094
wR_2 (all data)	0.0588
wR_2	0.0570
R_1 (all data)	0.0286
R_1	0.0249

A clear light red prism-shaped crystal with dimensions 0.27×0.23×0.22 mm^3 was mounted on a mylar loop with oil. Data were collected using a Bruker D8 Venture triumph Mo diffractometer equipped with an Oxford Cryosystems low-temperature device operating at T = 100.0 K. Data were measured using ϕ and ω scans' using MoK$_\alpha$ radiation. The total number of runs and images was based on the strategy calculation from the program APEX3 (Bruker, 2015) The maximum resolution that was achieved was Θ= 27.510° (0.77 Å).

The diffraction pattern was indexed. The total number of runs and images was based on the strategy calculation from the program APEX3 (Bruker, 2015) and the unit cell was refined using **SAINT** (Bruker, V8.40A, after 2013) on 9593 reflections, 27% of the observed reflections.

Data reduction, scaling and absorption corrections were performed using **SAINT** (Bruker, V8.40A, after 2013). The final completeness is 99.80 % out to 27.510° in Θ. A multi-scan absorption correction was performed using **SADABS**-2016/2 (Bruker, 2016/2) was used for absorption correction. wR_2(int) was 0.0477 before and 0.0402 after correction. The Ratio of minimum to maximum transmission is 0.9162. The absorption coefficient μ of this material is 1.054 mm^{-1} at this wavelength (λ = 0.711Å) and the minimum and maximum transmissions are 0.683 and 0.746.

The structure was solved and the space group *Pnna* (# 52) determined by the **ShelXT** (Sheldrick, 2015) structure solution program using Intrinsic Phasing and refined by Least Squares using version 2018/3 of **ShelXL** (Sheldrick, 2015). All non-hydrogen atoms were refined anisotropically. Hydrogen atom positions were calculated geometrically and refined using the riding model.

Table S6: Fractional Atomic Coordinates (×10^4) and Equivalent Isotropic Displacement Parameters (Å^2×10^3) for **Complex 4**. U_{eq} is defined as 1/3 of the trace of the orthogonalised U_{ij}.

Atom	x	y	z	U_{eq}
Ag	2500	5000	5742.0(2)	17.46(6)
Cl2	3737.1(5)	2500	2500	26.66(15)
P	4350.7(4)	7500	2500	17.52(14)
Cl1	5449.3(4)	3138.7(3)	2197.0(7)	38.02(14)
F6	4361.3(8)	6775.4(5)	2464.8(14)	28.5(3)
N2	4533.6(10)	5453.1(7)	6171.5(16)	15.8(3)
N1	3904.3(10)	5123.4(6)	6839.7(15)	14.0(3)
N3	5652.1(11)	5065.6(7)	7760.2(17)	22.0(3)
N4	5025.9(10)	4750.0(7)	8435.5(17)	20.8(3)
C7	2604.9(12)	4672.3(8)	9152.9(17)	14.7(3)
C9	6096.8(12)	5802.7(8)	6006.1(19)	18.0(4)
C8	5384.0(12)	5433.1(8)	6689.8(19)	16.7(3)
C14	5884.5(13)	6220.4(8)	4941(2)	21.7(4)
C10	7009.4(13)	5729.0(9)	6439(2)	23.4(4)
C2	3465.8(12)	4441.8(8)	8760.5(18)	15.1(3)
C1	4149.7(12)	4805.0(8)	8000.2(18)	15.2(3)
C6	1975.9(13)	4272.5(8)	9754.6(19)	18.5(4)
C3	3687.5(13)	3839.5(8)	9034.9(19)	20.4(4)
C13	6580.4(15)	6553.6(9)	4304(2)	27.8(4)
C11	7698.8(14)	6060.0(11)	5773(2)	30.6(5)
C4	3049.7(15)	3455.6(8)	9627(2)	24.2(4)
C5	2191.1(15)	3669.3(8)	9973(2)	23.3(4)
C12	7478.2(15)	6468.4(10)	4705(2)	32.5(5)
F2	3344.7(15)	7467.5(12)	1948(3)	35.1(6)
F4	3945(2)	7458.8(12)	4155(3)	34.4(6)
F5	5362.9(16)	7508.8(12)	3231(3)	36.6(6)
F3	4760(2)	7506.9(13)	1001(3)	41.4(7)
C15	4867(3)	2459.2(18)	1915(4)	24.0(8)

Table S7: Anisotropic Displacement Parameters (×10^4) **Complex 4.** The anisotropic displacement factor exponent takes the form: $-2\pi^2[h^2a^{*2} \times U_{11} + \ldots + 2hka^* \times b^* \times U_{12}]$

Atom	U_{11}	U_{22}	U_{33}	U_{23}	U_{13}	U_{12}
Ag	12.18(9)	24.43(10)	15.77(10)	0	0	-0.69(7)
Cl2	20.6(3)	35.6(4)	23.8(3)	1.5(3)	0	0
P	13.1(3)	15.8(3)	23.7(3)	-7.5(2)	0	0
Cl1	36.0(3)	33.9(3)	44.1(3)	-14.0(2)	11.5(2)	-12.5(2)
F6	26.8(6)	15.7(5)	43.0(7)	-8.0(5)	4.7(5)	-0.6(4)
N2	15.5(7)	17.1(7)	14.8(7)	-2.4(6)	0.5(6)	-1.0(6)
N1	14.8(7)	14.0(7)	13.2(7)	-2.2(5)	-0.2(5)	0.9(5)
N3	16.8(8)	27.4(8)	21.9(8)	-0.4(7)	-2.6(6)	0.8(6)
N4	16.1(7)	27.3(8)	19.1(8)	0.9(6)	-2.2(6)	2.2(6)
C7	15.7(8)	19.3(9)	9.1(7)	1.3(6)	-1.6(6)	1.8(7)
C9	15.8(8)	20.3(8)	17.9(8)	-9.2(7)	2.8(7)	-1.5(7)
C8	15.2(8)	18.5(8)	16.2(8)	-5.7(7)	0.3(7)	1.8(7)
C14	21.1(9)	22.8(9)	21.2(9)	-6.7(7)	3.5(7)	-2.9(7)
C10	18.4(9)	28.3(10)	23.4(10)	-10.8(8)	1.4(7)	-0.9(8)
C2	17.1(8)	16.5(8)	11.8(8)	0.4(6)	-1.7(6)	2.7(7)
C1	16.5(8)	15.6(7)	13.4(8)	-3.2(6)	-0.8(6)	3.8(6)
C6	20.2(9)	21.3(9)	14.1(8)	2.3(7)	1.9(7)	1.2(7)
C3	23.4(9)	19.3(9)	18.5(9)	0.3(7)	-0.6(7)	8.0(7)
C13	32.7(11)	27.1(10)	23.6(10)	-7.6(8)	8.4(8)	-8.3(9)
C11	16.4(9)	40.7(12)	34.6(12)	-17.9(10)	5.7(8)	-5.9(8)
C4	37.1(11)	14.4(8)	21.1(9)	4.9(7)	0.5(8)	6.4(8)
C5	30.7(10)	20.7(9)	18.5(9)	5.2(7)	3.2(8)	-3.7(8)
C12	27.7(11)	36.3(11)	33.5(11)	-14.1(9)	14.1(9)	-12.5(9)
F2	20.0(11)	24.9(11)	60.5(18)	-7.4(14)	-18.8(11)	0.6(10)
F4	52.9(17)	26.7(12)	23.7(13)	-5.1(10)	11.8(12)	-7.2(12)
F5	18.9(11)	25.0(12)	65.9(18)	-1.5(13)	-18.5(12)	0.5(10)
F3	60(2)	38.2(14)	25.4(14)	-8.9(11)	17.9(14)	-9.1(15)
C15	20.6(18)	27.3(19)	24.1(17)	-5.8(16)	1.6(15)	-1.4(16)

Table S8: Bond Lengths in Å for **Complex 4.**

Atom	Atom	Length/Å
Ag	N1[1]	2.3163(15)
Ag	N1	2.3164(15)
Ag	C10[2]	2.7014(18)
Ag	C10[3]	2.7014(18)
Cl2	C15	1.747(4)
Cl2	C15[4]	1.747(4)
P	F6[5]	1.6081(11)
P	F6	1.6080(11)
P	F2	1.564(2)
P	F2[5]	1.564(2)
P	F4	1.662(2)
P	F4[5]	1.662(2)
P	F5	1.634(2)
P	F5[5]	1.634(2)
P	F3[5]	1.525(3)
P	F3	1.525(3)
Cl1	C15[4]	1.782(4)
Cl1	C15	1.752(4)
N2	N1	1.333(2)
N2	C8	1.338(2)
N1	C1	1.345(2)
N3	N4	1.316(2)
N3	C8	1.350(2)
N4	C1	1.353(2)

Atom	Atom	Length/Å
C7	C7[1]	1.486(3)
C7	C2	1.410(2)
C7	C6	1.398(2)
C9	C8	1.474(3)
C9	C14	1.396(3)
C9	C10	1.407(3)
C14	C13	1.394(3)
C10	C11	1.396(3)
C2	C1	1.470(2)
C2	C3	1.399(2)
C6	C5	1.390(3)
C3	C4	1.381(3)
C13	C12	1.382(3)
C11	C12	1.387(3)
C4	C5	1.383(3)
F2	F2[5]	1.043(5)
F2	F4[5]	1.366(4)
F4	F3[5]	1.205(4)
F5	F5[5]	1.368(6)
F5	F3[5]	1.140(4)
C15	C15[4]	1.110(8)

----

[1]1/2-x,1-y,+z; [2]-1/2+x,+y,1-z; [3]1-x,1-y,1-z; [4]+x,1/2-y,1/2-z; [5]+x,3/2-y,1/2-z

Table S9: Bond Angles in ° for **Complex 4.**

Atom	Atom	Atom	Angle/°
N1^{1}	Ag	N1	127.36(7)
N1	Ag	C10^{2}	120.05(5)
N1^{1}	Ag	C10^{2}	99.75(5)
N1^{1}	Ag	C10^{3}	120.05(5)
N1	Ag	C10^{3}	99.75(5)
C10^{3}	Ag	C10^{2}	81.86(9)
C15^{4}	Cl2	C15	37.0(3)
F6	P	F6^{5}	178.89(10)
F6^{5}	P	F4	92.26(10)
F6	P	F4	88.14(10)
F6	P	F4^{5}	92.25(10)
F6^{5}	P	F4^{5}	88.14(10)
F6	P	F5	90.67(10)
F6^{5}	P	F5^{5}	90.67(10)
F6	P	F5^{5}	88.32(10)
F6^{5}	P	F5	88.32(10)
F2^{5}	P	F6^{5}	87.50(11)
F2	P	F6^{5}	93.55(11)
F2^{5}	P	F6	93.55(11)
F2	P	F6	87.50(11)
F2^{5}	P	F2	39.0(2)
F2	P	F4^{5}	50.00(14)
F2^{5}	P	F4	50.00(14)
F2	P	F4	88.16(16)
F2^{5}	P	F4^{5}	88.17(16)
F2	P	F5	174.22(16)
F2^{5}	P	F5^{5}	174.22(16)
F2^{5}	P	F5	135.83(15)
F2	P	F5^{5}	135.83(15)
F4^{5}	P	F4	138.1(2)
F5^{5}	P	F4	135.60(16)
F5	P	F4^{5}	135.60(16)
F5	P	F4	86.30(16)
F5^{5}	P	F4^{5}	86.30(16)
F5	P	F5^{5}	49.5(2)
F3^{5}	P	F6^{5}	89.28(12)
F3^{5}	P	F6	90.29(12)
F3	P	F6^{5}	90.28(12)
F3	P	F6	89.28(12)
F3	P	F2	93.85(17)
F3^{5}	P	F2^{5}	93.85(17)
F3^{5}	P	F2	132.35(17)
F3	P	F2^{5}	132.35(17)
F3^{5}	P	F4	44.19(16)
F3	P	F4	176.65(18)
F3	P	F4^{5}	44.19(16)
F3^{5}	P	F4^{5}	176.65(18)
F3	P	F5	91.6(2)
F3^{5}	P	F5^{5}	91.6(2)
F3	P	F5^{5}	42.13(16)
F3^{5}	P	F5	42.13(16)
F3	P	F3^{5}	133.7(3)
C15	Cl1	C15^{4}	36.6(3)
N1	N2	C8	117.15(15)
N2	N1	Ag	118.14(11)
N2	N1	C1	118.82(15)
C1	N1	Ag	122.26(12)
N4	N3	C8	118.33(16)
N3	N4	C1	118.01(15)
C2	C7	C7^{1}	122.69(19)
C6	C7	C7^{1}	118.97(18)
C6	C7	C2	117.70(16)
C14	C9	C8	121.40(17)
C14	C9	C10	119.64(17)
C10	C9	C8	118.95(17)
N2	C8	N3	124.06(17)
N2	C8	C9	118.98(16)
N3	C8	C9	116.84(16)
C13	C14	C9	119.64(19)
C9	C10	Ag^{3}	96.03(11)
C11	C10	Ag^{3}	77.57(11)
C11	C10	C9	119.9(2)
C7	C2	C1	122.51(15)
C3	C2	C7	120.43(17)
C3	C2	C1	117.01(16)
N1	C1	N4	122.99(16)
N1	C1	C2	119.78(15)
N4	C1	C2	116.89(15)
C5	C6	C7	121.37(17)
C4	C3	C2	120.37(17)
C12	C13	C14	120.5(2)
C12	C11	C10	119.8(2)
C3	C4	C5	119.90(17)
C4	C5	C6	120.13(18)
C13	C12	C11	120.5(2)
F2^{5}	F2	P	70.52(10)
F2^{5}	F2	F4^{5}	137.04(18)
F4^{5}	F2	P	68.71(15)
F2^{5}	F4	P	61.28(14)
F3^{5}	F4	P	61.87(17)
F3^{5}	F4	F2^{5}	122.6(2)
F5^{5}	F5	P	65.24(11)
F3^{5}	F5	P	63.83(17)
F3^{5}	F5	F5^{5}	128.98(18)
F4^{5}	F3	P	73.94(19)
F5^{5}	F3	P	74.05(19)
F5^{5}	F3	F4^{5}	147.9(3)
Cl2	C15	Cl1	111.7(2)
Cl2	C15	Cl1^{4}	110.3(2)
Cl1	C15	Cl1^{4}	109.7(2)
C15^{4}	C15	Cl2	71.48(13)
C15^{4}	C15	Cl1	73.2(3)
C15^{4}	C15	Cl1^{4}	70.2(4)

----

^{1}1/2-x,1-y,+z; ^{2}-1/2+x,+y,1-z; ^{3}1-x,1-y,1-z; ^{4}+x,1/2-y,1/2-z; ^{5}+x,3/2-y,1/2-z

Table S10: Hydrogen Fractional Atomic Coordinates (×10^4) and Equivalent Isotropic Displacement Parameters (Å^2×10^3) for **Complex 4**. U_{eq} is defined as 1/3 of the trace of the orthogonalised U_{ij}.

Atom	x	y	z	U_{eq}
H14	5269.07	6277.18	4652.27	26
H10	7156.14	5454.43	7183.66	28
H6	1389.15	4416.06	10019.77	22
H3	4280.26	3693.78	8812.44	24
H13	6436.22	6841.72	3587.17	33
H11	8316.98	6005.6	6049.78	37
H4	3200.34	3045.29	9797.04	29
H5	1747.56	3403.29	10360.95	28
H12	7948.05	6691.24	4247.09	39
H15	4907.61	2397.96	949.87	29

Table S11: Atomic Occupancies for all atoms that are not fully occupied in **Complex 4**.

Atom	Occupancy
F2	0.5
F4	0.5
F5	0.5
F3	0.5
C15	0.5
H15	0.5

Complex 6

Report created with ReportPlus

Crystal Data and Experimental

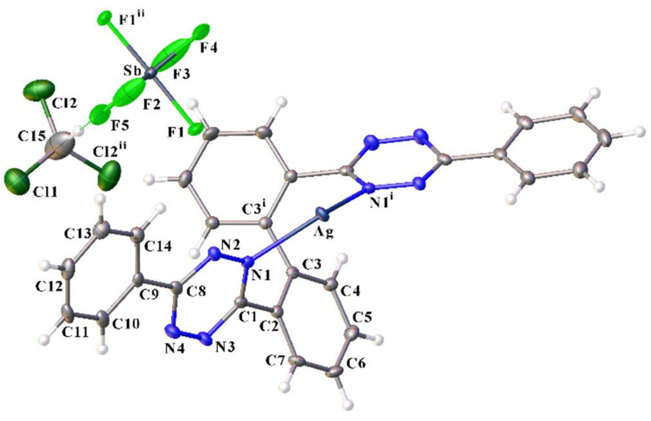

Figure S10: (i) x, 3/2-y, 1-z ; (ii) x, y, 3/2-z

Experimental. Single prism-shaped crystals of **Complex 6** were recrystallised from a mixture of trichloromethane and pentane by slow evaporation. A suitable crystal 0.23×0.22×0.20 mm³ was selected and mounted on a mylar loop with oil on an Bruker D8 Venture triumph Mo diffractometer. The crystal was kept at a steady T = 100 K during data collection. The structure was solved with the **ShelXT** (Sheldrick, 2015) structure solution program using the intrinsic phasing methods solution method and by using **Olex2** (Dolomanov et al., 2009) as the graphical interface. The model was refined with version 2018/3 of **ShelXL** (Sheldrick, 2015) using Least Squares minimisation.

Crystal Data. $C_{29}H_{19}AgCl_3F_6N_8Sb$, M_r = 929.49, orthorhombic, *Pbcm* (No. 57), a = 9.2516(5) Å, b = 14.7128(9) Å, c = 23.0829(13) Å, $\alpha = \beta = \gamma = 90^\circ$, V = 3142.0(3) Å³, T = 100 K, Z = 4, Z' = 0.5, μ(MoK_α) = 1.811, 43275 reflections measured, 3699 unique (R_{int} = 0.0462) which were used in all calculations. The final wR_2 was 0.0759 (all data) and R_1 was 0.0337 (I > 2(I)).

**Compound**	**Complex 6**
**CCDC**	**2068274**
Formula	$C_{29}H_{19}AgCl_3N_8$, SbF_6
$D_{calc.}$/ g cm^{-3}	1.965
μ/mm^{-1}	1.811
Formula Weight	929.49
Colour	red
Shape	prism
Size/mm³	0.23×0.22×0.20
T/K	100
Crystal System	orthorhombic
Space Group	*Pbcm*
a/Å	9.2516(5)
b/Å	14.7128(9)
c/Å	23.0829(13)
$\alpha/^\circ$	90
$\beta/^\circ$	90
$\gamma/^\circ$	90
V/Å³	3142.0(3)
Z	4
Z'	0.5
Wavelength/Å	0.71073
Radiation type	MoK_α
$\Theta_{min}/^\circ$	3.143
$\Theta_{max}/^\circ$	27.549
Measured Refl.	43275
Independent Refl.	3699
Reflections with I > 2(I)	3151
R_{int}	0.0462
Parameters	228
Restraints	0
Largest Peak	0.862
Deepest Hole	-1.437
GooF	1.074
wR_2 (all data)	0.0759
wR_2	0.0721
R_1 (all data)	0.0450
R_1	0.0337

A prism-shaped crystal with dimensions 0.23×0.22×0.20 mm^3 was mounted on a mylar loop with oil. Data were collected using a Bruker D8 Venture triumph Mo diffractometer equipped with an Oxford Cryosystems low-temperature device operating at T = 100 K. Data were measured using ϕ and ω scans' using MoK$_{\alpha}$ radiation. The total number of runs and images was based on the strategy calculation from the program APEX3 (Bruker, 2015) The maximum resolution that was achieved was Θ = 27.549° (0.77 Å). The diffraction pattern was indexed. The total number of runs and images was based on the strategy calculation from the program APEX3 (Bruker, 2015) and the unit cell was refined using **SAINT** (Bruker, V8.38A, after 2013) on 9831 reflections, 23% of the observed reflections.

Data reduction, scaling and absorption corrections were performed using **SAINT** (Bruker, V8.40A, after 2013). The final completeness is 99.80 % out to 27.549° in Θ. A multi-scan absorption correction was performed using **SADABS**-2016/2 (Bruker, 2016/2) was used for absorption correction. wR_2(int) was 0.0581 before and 0.0535 after correction. The Ratio of minimum to maximum transmission is 0.9317. The absorption coefficient μ of this material is 1.811 mm^{-1} at this wavelength (λ = 0.711Å) and the minimum and maximum transmissions are 0.695 and 0.746.

The structure was solved and the space group *Pbcm* (# 57) determined by the **ShelXT** (Sheldrick, 2015) structure solution program using intrinsic phasing methods and refined by Least Squares using version 2018/3 of **ShelXL** (Sheldrick, 2015). All non-hydrogen atoms were refined anisotropically. Hydrogen atom positions were calculated geometrically and refined using the riding model. Hydrogen atom positions were calculated geometrically and refined using the riding model.

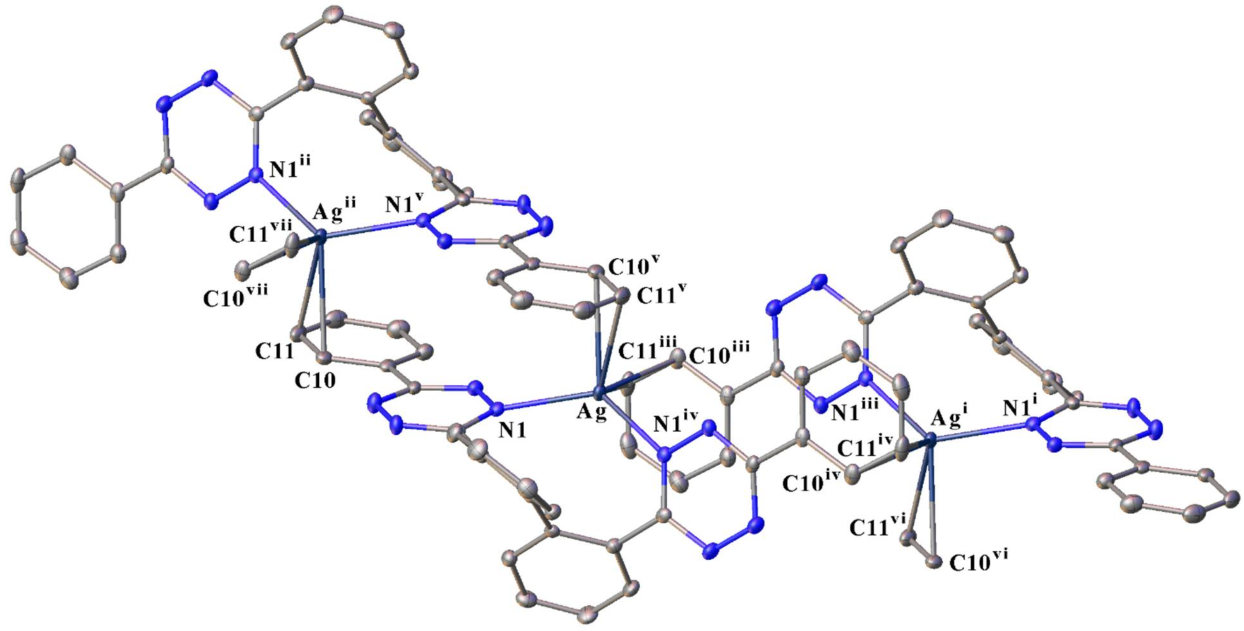

Figure S11: (i) 1-x, 2-y, 1-z ; (ii) 1-x, -1/2+y, +z ; (iii) 1-x, 1/2+y, z, (iv) x, 3/2-y, 1-z ; (v) 1-x, 1-y, 1-z ; (vi) x, 1+y, z ; (vii) x, 1/2-y, 1-z

Table S12: Fractional Atomic Coordinates (×10^4^) and Equivalent Isotropic Displacement Parameters ($Å^2×10^3$) for **Complex 6.** U_{eq} is defined as 1/3 of the trace of the orthogonalised U_{ij}.

Atom	x	y	z	U_{eq}
Ag	4235.6(4)	7500	5000	14.61(9)
C1	1965(3)	5855(2)	4813.5(14)	12.0(6)
N1	3124(3)	6099.2(18)	5121.3(11)	12.4(6)
C2	1190(3)	6538(2)	4463.4(14)	13.2(7)
N2	3798(3)	5478.6(18)	5442.1(12)	13.9(6)
C3	808(3)	7395(2)	4685.7(13)	11.4(6)
N3	1534(3)	4978.1(19)	4756.6(13)	16.9(6)
C4	206(4)	8018(2)	4301.3(14)	14.3(7)
N4	2212(3)	4358.3(19)	5061.4(13)	17.9(6)
C5	-33(4)	7803(3)	3725.1(15)	18.9(7)
C6	291(4)	6944(3)	3519.8(15)	20.5(8)
C7	900(4)	6310(2)	3889.7(15)	17.7(7)
C8	3288(4)	4625(2)	5421.0(14)	13.2(7)
C9	3984(4)	3919(2)	5774.1(14)	13.8(7)
C10	3570(4)	3007(2)	5690.1(16)	17.4(7)
C11	4273(4)	2317(2)	5997.6(17)	21.3(8)
C12	5348(4)	2542(3)	6388.4(17)	23.8(8)
C13	5715(4)	3438(3)	6486.6(16)	22.3(8)
C14	5044(4)	4129(2)	6176.9(14)	17.3(7)
Sb	7380.9(4)	5653.0(2)	7500	14.17(9)
F1	7389(2)	5634.5(14)	6685.0(8)	22.3(4)
F2	8652(5)	4674(3)	7500	58.3(14)
F3	6067(6)	6599(4)	7500	80.7(19)
F4	8899(5)	6465(3)	7500	67.5(16)
F5	5865(5)	4828(4)	7500	67.6(16)
Cl1	2631(2)	3725.9(13)	7500	48.0(4)
Cl2	2057.8(15)	5403.0(9)	8127.2(6)	50.7(3)
C15	2553(13)	4865(5)	7500	72(3)

Table S13: Anisotropic Displacement Parameters (×10^4^) **Complex 6**. The anisotropic displacement factor exponent takes the form: $-2\pi^2[h^2a^{*2} \times U_{11} + ... +2hka^* \times b^* \times U_{12}]$

Atom	U_{11}	U_{22}	U_{33}	U_{23}	U_{13}	U_{12}
Ag	14.59(17)	8.21(16)	21.03(18)	-0.16(14)	0	0
C1	10.4(15)	14.1(16)	11.6(15)	-2.8(12)	2.9(12)	-0.5(12)
N1	13.5(13)	12.1(13)	11.4(13)	-1.6(11)	1.4(11)	0.6(11)
C2	13.2(15)	13.0(16)	13.3(15)	-0.1(13)	-0.1(13)	-1.7(13)
N2	13.8(13)	11.5(14)	16.3(14)	0.6(11)	1.6(11)	1.1(11)
C3	7.8(14)	13.5(16)	12.8(16)	-0.8(13)	0.3(12)	-1.1(12)
N3	18.4(15)	11.2(14)	21.1(15)	-1.0(12)	-1.8(12)	-3.5(12)
C4	14.7(16)	14.0(16)	14.1(15)	-1.5(13)	0.2(13)	1.2(13)
N4	19.5(15)	12.2(14)	22.1(15)	-1.3(12)	-3.4(12)	-1.7(12)
C5	18.5(17)	22.8(18)	15.3(16)	4.0(14)	-3.9(14)	-0.6(15)
C6	25.2(19)	23.6(19)	12.6(16)	-4.9(14)	-4.4(14)	-0.5(16)
C7	19.7(18)	15.1(16)	18.3(17)	-6.3(13)	-2.5(14)	-0.5(14)
C8	12.9(15)	10.8(15)	15.9(16)	-2.1(13)	4.7(13)	-0.4(13)
C9	15.7(16)	12.4(15)	13.4(15)	1.3(13)	7.1(13)	1.4(13)
C10	16.1(17)	14.3(17)	21.8(18)	-0.1(14)	8.6(14)	-0.4(14)
C11	25.6(19)	9.8(17)	28.4(19)	2.5(14)	11.1(16)	0.5(14)
C12	27(2)	18.6(17)	25.9(19)	8.6(16)	7.8(16)	7.1(16)
C13	22.0(18)	24.3(19)	20.6(18)	2.3(15)	0.8(15)	6.1(16)
C14	17.7(17)	15.0(17)	19.2(17)	-0.1(14)	2.7(15)	2.2(14)
Sb	18.99(17)	13.67(16)	9.86(14)	0	0	-0.59(13)
F1	33.5(12)	22.9(11)	10.6(9)	-0.9(8)	-2.6(9)	-4.1(10)
F2	87(3)	61(3)	26.7(19)	0	0	59(3)
F3	124(4)	91(4)	27(2)	0	0	93(4)

Atom	U_{11}	U_{22}	U_{33}	U_{23}	U_{13}	U_{12}
F4	93(3)	96(4)	13.2(16)	0	0	-80(3)
F5	69(3)	109(4)	24.8(19)	0	0	-74(3)
Cl1	41.4(9)	40.6(9)	61.9(11)	0	0	-0.1(8)
Cl2	61.7(8)	48.8(7)	41.6(7)	-12.1(6)	-15.1(6)	20.6(6)
C15	132(9)	36(4)	47(5)	0	0	31(5)

Table S14: Bond Lengths in Å for **Complex 6**

Atom	Atom	Length/Å
Ag	N1	2.320(3)
Ag	N1[1]	2.320(3)
Ag	C10[2]	2.686(3)
Ag	C10[3]	2.686(3)
Ag	C11[2]	2.698(4)
Ag	C11[3]	2.698(4)
C1	N1	1.336(4)
C1	C2	1.475(5)
C1	N3	1.357(4)
N1	N2	1.331(4)
C2	C3	1.406(4)
C2	C7	1.392(5)
N2	C8	1.342(4)
C3	C3[1]	1.484(6)
C3	C4	1.393(5)
N3	N4	1.312(4)
C4	C5	1.385(5)
N4	C8	1.354(4)
C5	C6	1.382(5)
C6	C7	1.384(5)
C8	C9	1.469(5)
C9	C10	1.410(5)
C9	C14	1.386(5)
C10	C11	1.399(5)
C11	C12	1.383(6)
C12	C13	1.380(5)
C13	C14	1.390(5)
Sb	F1[4]	1.8815(19)
Sb	F1	1.8815(19)
Sb	F2	1.860(4)
Sb	F3	1.848(4)
Sb	F4	1.843(4)
Sb	F5	1.854(4)
Cl1	C15	1.678(8)
Cl2	C15	1.712(4)

----
[1]+x,3/2-y,1-z; [2]1-x,1/2+y,+z; [3]1-x,1-y,1-z; [4]+x,+y,3/2-z

Table S15: Bond Angles in ° for **Complex 6**.

Atom	Atom	Atom	Angle/°
N1	Ag	N1[1]	127.39(14)
N1[1]	Ag	C10[2]	99.20(10)
N1[1]	Ag	C10[3]	120.66(10)
N1	Ag	C10[2]	120.66(10)
N1	Ag	C10[3]	99.20(10)
N1	Ag	C11[2]	91.99(10)
N1[1]	Ag	C11[3]	91.99(10)
N1	Ag	C11[3]	114.74(10)
N1[1]	Ag	C11[2]	114.74(10)
C10[2]	Ag	C10[3]	81.80(16)
C10[2]	Ag	C11[3]	95.27(12)
C10[3]	Ag	C11[2]	95.27(12)
C10[3]	Ag	C11[3]	30.13(11)
C10[2]	Ag	C11[2]	30.13(11)
C11[2]	Ag	C11[3]	118.49(17)
N1	C1	C2	119.9(3)
N1	C1	N3	122.9(3)
N3	C1	C2	116.9(3)
C1	N1	Ag	122.0(2)
N2	N1	Ag	118.0(2)
N2	N1	C1	119.2(3)
C3	C2	C1	122.2(3)
C7	C2	C1	116.8(3)
C7	C2	C3	120.9(3)
N1	N2	C8	117.2(3)
C2	C3	C3[1]	122.9(3)
C4	C3	C2	117.3(3)
C4	C3	C3[1]	119.1(3)
N4	N3	C1	118.0(3)
C5	C4	C3	121.7(3)
N3	N4	C8	118.6(3)
C6	C5	C4	120.2(3)
C5	C6	C7	119.5(3)
C6	C7	C2	120.2(3)
N2	C8	N4	123.5(3)
N2	C8	C9	119.2(3)
N4	C8	C9	117.2(3)
C10	C9	C8	118.6(3)
C14	C9	C8	121.6(3)
C14	C9	C10	119.8(3)
C9	C10	Ag[3]	98.1(2)
C11	C10	Ag[3]	75.4(2)
C11	C10	C9	119.7(3)
C10	C11	Ag[3]	74.5(2)
C12	C11	Ag[3]	99.5(2)
C12	C11	C10	119.4(3)
C13	C12	C11	120.9(3)
C12	C13	C14	120.3(4)
C9	C14	C13	119.9(3)
F1[4]	Sb	F1	178.28(13)
F2	Sb	F1	89.21(7)
F2	Sb	F1[4]	89.21(7)

Atom	Atom	Atom	Angle/°
F3	Sb	F1[4]	90.78(7)
F3	Sb	F1	90.78(7)
F3	Sb	F2	178.1(3)
F3	Sb	F5	89.7(3)
F4	Sb	F1	90.36(7)
F4	Sb	F1[4]	90.36(7)
F4	Sb	F2	91.2(3)
F4	Sb	F3	90.8(3)
F4	Sb	F5	179.5(3)
F5	Sb	F1	89.63(7)
F5	Sb	F1[4]	89.63(7)
F5	Sb	F2	88.3(3)
Cl1	C15	Cl2	118.2(3)
Cl1	C15	Cl2[4]	118.2(3)
Cl2	C15	Cl2[4]	115.5(5)

––––

[1]+x,3/2-y,1-z; [2]1-x,1/2+y,+z; [3]1-x,1-y,1-z; [4]+x,+y,3/2-z

Table S16: Hydrogen Fractional Atomic Coordinates (×10^4) and Equivalent Isotropic Displacement Parameters (Å^2×10^3) for **Complex 6.** U_{eq} is defined as 1/3 of the trace of the orthogonalised U_{ij}.

**Atom**	**x**	**y**	**z**	U_{eq}
H4	-47.93	8606.36	4437.81	17
H5	-420.77	8247.3	3469.95	23
H6	97.24	6790.39	3127.74	25
H7	1119.71	5718.1	3751.56	21
H10	2816.82	2861.06	5426.08	21
H11	4014.35	1698.79	5938.38	26
H12	5839.34	2073.44	6591.98	29
H13	6430.63	3582.43	6767.13	27
H14	5311.17	4744.29	6241.08	21
H15	3609.95	5006.51	7500	86

Copper-catalyzed tweezer 3 synthesis (*Angew. Chem. Int. Ed.*, 2020, 59, 1149)

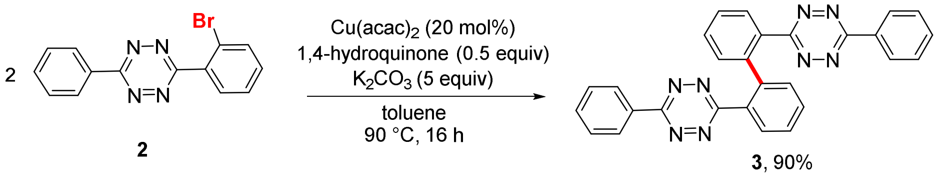

3-(2-bromophenyl)-6-phenyl-1,2,4,5-tetrazine **2** (0.225 g, 0.716 mmol, 1.0 equiv), $Cu(acac)_2$ (37.48 mg, 0.143 mmol, 20 mol%), 1.4-hydroquinone (39.0 mg, 0.358 mmol, 50 mol%) and K_2CO_3 (0.494 g, 3.58 mmol, 5 equiv) were introduced into a dried Schlenk tube. After 3 standard cycles of evacuating and back-filling with argon, toluene (3 ml, 0.24 M) was added. The reaction mixture was stirred and heated at 90 °C for 16 h. After allowing the reaction to cool to room temperature, the resulting mixture was diluted water (50 ml) and extracted with dichloromethane (3 x 30 ml). The combined organic layer was dried over anhydrous $MgSO_4$, filtrated and the solvent was evaporated under reduced pressure. The crude product was purified by silica gel column chromatography (eluent: heptane-dichloromethane by gradient 8:2…5:5 (v/v)) to afford **3** (pink solid) in 90% (0.150 g).

3,3'-[(1,1'-biphenyl)-2,2'-diyl]-6,6'-bis[phenyl]-1,2,4,5-tetrazine (3)

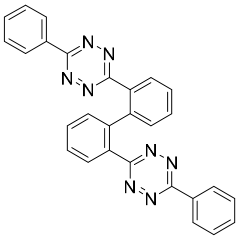

Rf = 0.32 (Dichloromethane-Heptane = 8:2 (v/v)).

^{1}H NMR (400 MHz, CD_2Cl_2): δ (ppm) = 8.54–8.47 (m, 4H), 8.06 (dd, J = 7.4, 1.6 Hz, 2H), 7.70–7.56 (m, 10H), 7.52 (dd, J = 7.2, 1.6 Hz, 2H).

^{13}C NMR (101 MHz, CD_2Cl_2): δ (ppm) = 167.05, 163.37, 141.89, 133.53, 132.71, 132.57, 132.53, 132.29, 131.31, 130.06, 129.09, 128.85.

Coordination polymers synthesis

[Ag(3)][PF_6] complex (4)

To a dried Schlenk tube equipped with a stir bar were added **3** (12 mg, 0.026 mmol) and $AgPF_6$ (6.6 mg, 0.026 mmol). After 3 standard cycles of evacuating and back-filling with argon, dichloromethane (0.013 M) was added and the reaction mixture was stirred for 30 minutes at room temperature until it was a uniform red-oragne color. Hexane (5 mL) was added, and **4** (16.5 mg, 88 %) was collected as a red solid via filtration. Single crystals of **4** were grown from a chloroform/hexane solution by slow evaporation.

^{1}H NMR (500 MHz, CD_2Cl_2) δ (ppm) = 8.30 (dd, *J* = 8.2, 1.4 Hz, 4H), 7.87 (dd, *J* = 7.7, 1.4 Hz, 2H), 7.70-7.63 (m, 2H), 7.52 (t, *J* = 7.8 Hz, 4H), 7.35 (td, *J* = 7.6, 1.3 Hz, 2H), 7.28 (td, *J* = 7.6, 1.4 Hz, 2H), 6.78 (dd, *J* = 7.8, 1.2 Hz, 2H).

^{13}C NMR (126 MHz, CD_2Cl_2) δ (ppm) = 165.8, 162.2, 138.6, 132.6, 131.3, 131.2, 131.0, 130.7, 130.6, 129.9, 128.6, 127.8, 127.4, 116.4.

165.1, 161.58, 138.0, 131.9, 130.6, 130.5, 130.4, 130.1, 129.5, 127.9, 127.3, 127.0, 115.9.

^{31}P NMR (202 MHz, CD_2Cl_2) δ (ppm) = –144.53 (sept, *J* = 711.0 Hz).

^{19}F NMR (470 MHz, CD_2Cl_2) δ (ppm) = –72.86 (d, *J* = 709.0 Hz).

HRMS + p ESI (m/z) [M^+] Calcd for $C_{28}H_{18}AgN_8$: 573.0699. Found: m/z = 573.0689, Δ = –1.745 ppm.

Calcd for $C_{56}H_{36}AgN_{16}$: 1039,2354. Found: m/z = 1039.2349, Δ = –0.481 ppm.

Calcd for $C_{56}H_{36}Ag_2N_{16}PF_6$: 1291.1047. Found: m/z = 1291.1009, Δ = –2.889 ppm.

[Ag(3)][PF$_6$]$_\infty$ complex (4a)

To a dried Schlenk tube equipped with a stir bar were added **3** (12 mg, 0.026 mmol) and $AgPF_6$ (13.1 mg, 0.052 mmol). After 3 standard cycles of evacuating and back-filling with argon, dichloromethane (0.026 M) was added and the reaction mixture was stirred for 2 hours at room temperature until it was a uniform orange color. Hexane (5 mL) was added, and **4a** (17.5 mg, 94 %) was collected as a red solid via filtration.

^{1}H NMR (500 MHz, Acetone-*d$_6$*) δ (ppm) = 8.50 (dd, *J* = 7.2, 1.8 Hz, 4H), 8.08 (dd, *J* = 7.1, 1.9 Hz, 2H), 7.81–7.76 (m, 2H), 7.72 (dd, *J* = 8.4, 6.9 Hz, 4H), 7.70-7.62 (m, 4H), 7.39 (dd, *J* = 7.2, 1.7 Hz, 2H).

^{13}C NMR (126 MHz, Acetone-*d$_6$*) δ (ppm) = 166.7, 162.7, 140.3, 132.9, 132.3, 132.2, 131.8, 131.7, 131.0, 129.4, 128.5, 127.9.

^{31}P NMR (202 MHz, Acetone-*d$_6$*) δ (ppm) = –144.30 (sept, *J* = 707.0 Hz).

^{19}F NMR (470 MHz, Acetone-*d$_6$*) δ (ppm) = –72.69 (d, *J* = 705.0 Hz).

[Ag(3)][BF$_4$] complex (5)

To a dried Schlenk tube equipped with a stir bar were added **3** (24 mg, 0.052 mmol) and $AgBF_4$ (10.1 mg, 0.052 mmol). After 3 standard cycles of evacuating and back-filling with argon, dichloromethane (0.013 M) was added and the reaction mixture was stirred for 15 minutes at room temperature until it was a uniform

red color. Hexane (10 mL) was added, and **5** (34 mg, 99 %) was collected as a red solid via filtration.

^{1}H NMR (500 MHz, CD_2Cl_2) δ (ppm) = 8.39–8.28 (m, 4H), 7.90 (dd, J = 7.6, 1.6 Hz, 2H), 7.69–7.61 (m, 2H), 7.54 (t, J = 7.9 Hz, 4H), 7.38 (dtd, J = 20.3, 7.5, 1.5 Hz, 4H), 6.98 (dd, J = 7.5, 1.5 Hz, 2H).

^{13}C NMR (126 MHz, CD_2Cl_2) δ (ppm) = 166.8, 163.1, 139.2, 133.7, 132.3, 132.1, 131.9, 131.6, 130.6, 129.5, 128.8, 128.3.

^{19}F NMR (470 MHz, CD_2Cl_2) δ (ppm) = –53.43.

HRMS + p ESI (m/z) [M^+] Calcd for $C_{28}H_{18}AgN_8$: 573.0699. Found: m/z = 573.0693, Δ = –1.152 ppm.

Calcd for $C_{56}H_{36}AgN_{16}$: 1039.2354. Found: m/z = 1039.2353, Δ = –0.115 ppm.

Calcd for $C_{56}H_{36}Ag_2N_{16}BF_4$: 1233.1434. Found: m/z = 1233.1426, Δ = –0.673 ppm.

[Ag(3)][SbF_6] complex (6)

To a dried Schlenk tube equipped with a stir bar were added **3** (12 mg, 0.026 mmol) and $AgSbF_6$ (8.9 mg, 0.026 mmol). After 3 standard cycles of evacuating and back-filling with argon, dichloromethane (0.013 M) was added and the reaction mixture was stirred for 15 minutes at room temperature until it was a uniform red color. Hexane (5 mL) was added, and **6** (20.5 mg, 97 %) was collected as a red solid via filtration. Single crystals of **6** were grown from chloroform/hexane solution by slow evaporation.

^{1}H NMR (500 MHz, CD_2Cl_2) δ (ppm) = 8.40–8.30 (m, 4H), 7.97–7.88 (m, 2H), 7.73–7.65 (m, 2H), 7.55 (t, J = 7.9 Hz, 4H), 7.46–7.38 (m, 2H), 7.30 (td, J = 7.7, 1.4 Hz, 2H), 6.76 (dd, J = 7.8, 1.2 Hz, 2H).

^{13}C NMR (126 MHz, CD_2Cl_2) δ (ppm) = 166.9, 163.4, 138.4, 134.1, 132.6, 132.3, 132.2, 132.1, 130.1, 129.7, 129.1, 128.4.

HRMS + p ESI (m/z) [M^+] Calcd for $C_{28}H_{18}AgN_8$: 573.0699. Found: m/z = 573.0689, Δ = –1.745 ppm.

Calcd for $C_{56}H_{36}AgN_{16}$: 1039.2354. Found: m/z = 1039.2349, Δ = –0.481 ppm.

Calcd for $C_{56}H_{36}Ag_2N_{16}SbF_6$: 1291.1047. Found: m/z = 1291.1009, Δ = –2.889 ppm.

[Ag(3)][ClO_4] complex (7)

To a dried Schlenk tube equipped with a stir bar were added **3** (24 mg, 0.052 mmol) and AgO_4Cl (10.6 mg, 0.052 mmol). After 3 standard cycles of evacuating and back-filling with argon, the mixture of dichloromethane/methanol 2:1 ratio (0.013 M) was added and the reaction mixture was stirred for 1 hours

at room temperature until it was a uniform red color. Hexane (10 mL) was added, and **7** (34.5 mg, 98 %) was collected as a red solid via filtration.

^{1}H NMR (600 MHz, $CDCl_3$+CD_3OD) δ (ppm) = 8.49–8.32 (m, 4H), 8.02 (d, *J* = 7.7 Hz, 2H), 7.65–7.55 (m, 2H), 7.51 (dt, *J* = 15.4, 7.6 Hz, 6H), 7.40 (td, *J* = 7.6, 1.4 Hz, 2H), 6.94 (d, *J* = 7.7 Hz, 2H).

^{13}C NMR (126 MHz, $CDCl_3$+CD_3OD) δ (ppm) = 166.1, 162.4, 137.6, 132.7, 131.5, 131.3, 131.2, 131.1, 129.6, 128.5, 128.1, 127.6.

HRMS + p ESI (m/z) [M^+] Calcd for $C_{28}H_{18}AgN_8$: 573.0699. Found: m/z = 573.0695, Δ = –0.733 ppm. Calcd for $C_{56}H_{36}AgN_{16}$: 1039.2354. Found: m/z = 1039.2347, Δ = –0.702 ppm.

[Ag(3)][NTf$_2$] complex (8)

To a dried Schlenk tube equipped with a stir bar were added **3** (12 mg, 0.026 mmol) and $AgN(Tf)_2$ (10.1 mg, 0.026 mmol). After 3 standard cycles of evacuating and back-filling with argon, dichloromethane (0.013 M) was added and the reaction mixture was stirred for 15 minutes at room temperature until it was a uniform red color. Hexane (5 mL) was added, and **8** (21 mg, 97 %) was collected as a red solid via filtration.

^{1}H NMR (500 MHz, CD_2Cl_2) δ (ppm) = 8.60–8.44 (m, 4H), 8.07 (dd, *J* = 7.8, 1.3 Hz, 2H), 7.76–7.66 (m, 2H), 7.62 (dd, *J* = 8.5, 7.1 Hz, 4H), 7.57 (td, *J* = 7.6, 1.2 Hz, 2H), 7.38 (td, *J* = 7.7, 1.3 Hz, 2H), 6.79 (dd, *J* = 7.8, 1.2 Hz, 2H).

^{13}C NMR (126 MHz, CD_2Cl_2) δ (ppm) = 167.4, 163.7, 138.5, 134.1, 132.9, 132.5, 132.4, 132.3, 130.5, 129.8, 129.3, 128.7, 119,7 (q, *J* = 322.56 Hz).

^{19}F NMR (470 MHz, CD_2Cl_2) δ (ppm) = –74.14.

HRMS + p ESI (m/z) [M^+] Calcd for $C_{28}H_{18}AgN_8$: 573.0699. Found: m/z = 573.0700, Δ = –0.020 ppm. Calcd for $C_{56}H_{36}AgN_{16}$: 1039.2354. Found: m/z = 1039.2356, Δ = –0.212 ppm.

[Ag(3)][OTf] complex (9)

To a dried Schlenk tube equipped with a stir bar were added **3** (20.0 mg, 0.043mmol) and AgOTf (11.04 mg, 0.043 mmol). After 3 standard cycles of evacuating and back-filling with argon, the dichloromethane

(0.013 M) was added and the reaction mixture was stirred for 15 minutes at room temperature until it was a uniform red color. Hexane (5 mL) was added, and **9** (0.041 mg, 95 %) was collected as a red solid via filtration.

^{1}H NMR (500 MHz, $CDCl_3$+CD_3OD) δ (ppm) = 8.47–8.41 (m, 4H), 8.00 (dd, J = 7.8, 1.3 Hz, 2H), 7.67–7.62 (m, 2H), 7.59–7.50 (m, 6H), 7.44 (td, J = 7.6, 1.4 Hz, 2H), 7.04 (dd, J = 7.8, 1.2 Hz, 2H).

^{13}C NMR (126 MHz, $CDCl_3$+CD_3OD) δ (ppm) = 167.0, 163.3, 139.7, 133.8, 132.4, 132.3, 132.2, 132.1, 131.9, 130.9, 129.7, 129.1, 128.6.

^{19}F NMR (470 MHz, $CDCl_3$+CD_3OD) δ (ppm) = –78.70.

HRMS + p ESI (m/z) [M^+] Calcd for $C_{28}H_{18}AgN_8$: 573.0699. Found: m/z = 573.0661, Δ = –6.753 ppm. Calcd for $C_{56}H_{36}AgN_{16}$: 1039.2354. Found: m/z = 1039.2323, Δ = –2.944 ppm.

[Cu(3)][BF_4] complex (10)

To a dried Schlenk tube equipped with a stir bar were added **3** (12 mg, 0.026 mmol) and [$Cu(CH_3CN)_4BF_4$] (8.2 mg, 0.026 mmol). After 3 standard cycles of evacuating and back-filling with argon, dichloromethane (0.013 M) was added. The reaction mixture was stirred for 10 min at room temperature. The dark solution was filtered through Celite and the filtrate was evaporated under reduced pressure yield **10** dark solid (22 mg, 78%).

^{1}H NMR (500 MHz, CD_2Cl_2) δ (ppm) = 7.98–7.89 (m, 4H), 7.54 (q, J = 10.2, 8.8 Hz, 4H), 7.36 (t, J = 7.6 Hz, 4H), 7.10 (s, 4H), 6.71 (d, J = 6.5 Hz, 2H).

^{13}C NMR (126 MHz, CD_2Cl_2) δ (ppm) = 134.0, 132.2, 132.1, 130.9, 129.5, 129.1, 128.4.

^{11}B NMR (160 MHz, CD_2Cl_2) δ (ppm) = –0.83.

HRMS + p ESI (m/z) [M^+] Calcd for $C_{28}H_{18}CuN_8$: 529.0945. Found: m/z = 529.0948, Δ = 0.586 ppm. Calcd for $C_{56}H_{36}CuN_{16}$: 995.2599. Found: m/z = 995.2613, Δ = 1.427 ppm.

[Pd(3)][$(BF_4)_2$] complex (11)

To a dried Schlenk tube equipped with a stir bar were added **3** (12 mg, 0.026 mmol) and [$Pd(CH_3CN)_4(BF_4)_2$] (11.5 mg, 0.026 mmol). After 3 standard cycles of evacuating and back-filling with argon, dichloromethane (0.013 M) was added. The reaction mixture was stirred for 10 min at room

temperature. The dark brown solution was evaporated and the precipitate was washed with hexane (5 mL) to yield **11** (0.018 mg, 95 %) as a dark brown solid.

HRMS + p ESI (m/z) [M^+] Calcd for $C_{28}H_{18}N_8Pd$: 572.0683. Found: m/z = 572.0664, Δ = -3.421 ppm. Calcd for $C_{56}H_{35}N_{16}Pd$: 1037.2259. Found: m/z = 1037.2271, Δ = 1.095 ppm.

[Pd(3)Cl] complex (12)

To a dried Schlenk tube equipped with a stir bar were added **3** (12 mg, 0.026 mmol) and [$Pd(CH_3CN)_2.Cl_2$] (6.7 mg, 0.026 mmol). After three standard cycles of evacuating and back-filling with argon, dichloromethane (0.013 M) was added. The reaction mixture was stirred for 10 min at room temperature. The dark brown solution was evaporated and the precipitate was washed with hexane (5 mL) to yield **12** (0.014 mg, 89 %) as a red solid.

HRMS + p ESI (m/z) [M^+] Calcd for $C_{28}H_{18}ClN_8Pd$: 607.0372. Found: m/z = 607.0388, Δ = 2.620 ppm.

Copy of ^{1}H and ^{13}C NMR spectra

3,3'-[(1,1'-biphenyl)-2,2'-diyl]-6,6'-bis[phenyl]-1,2,4,5-tetrazine (3)

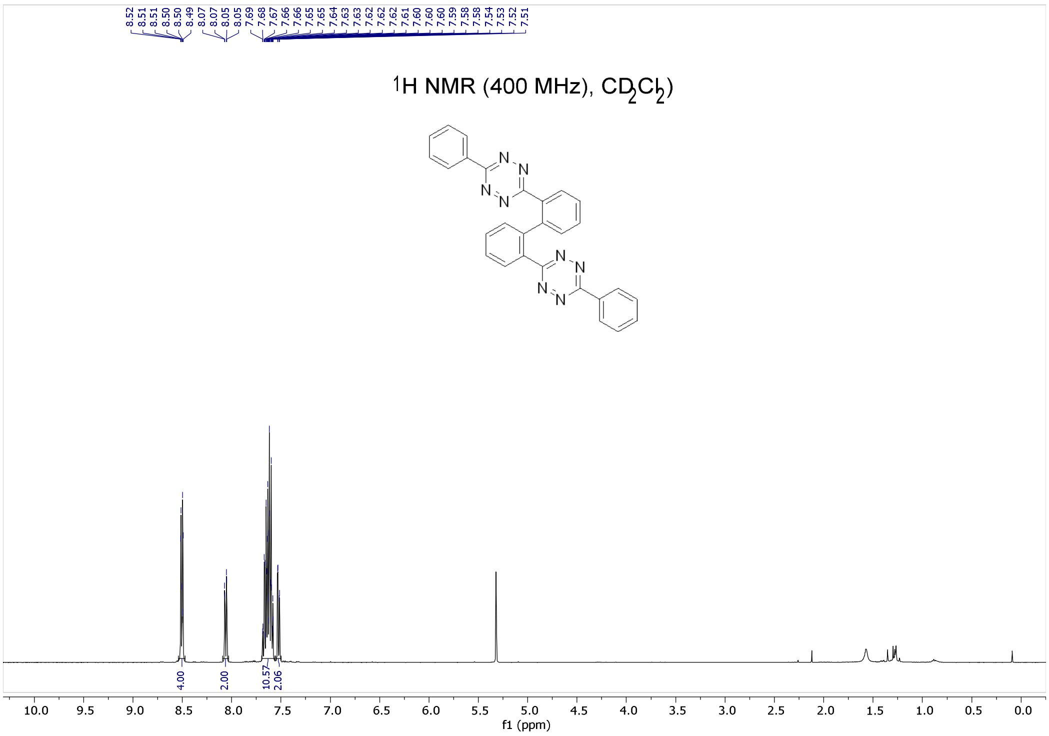

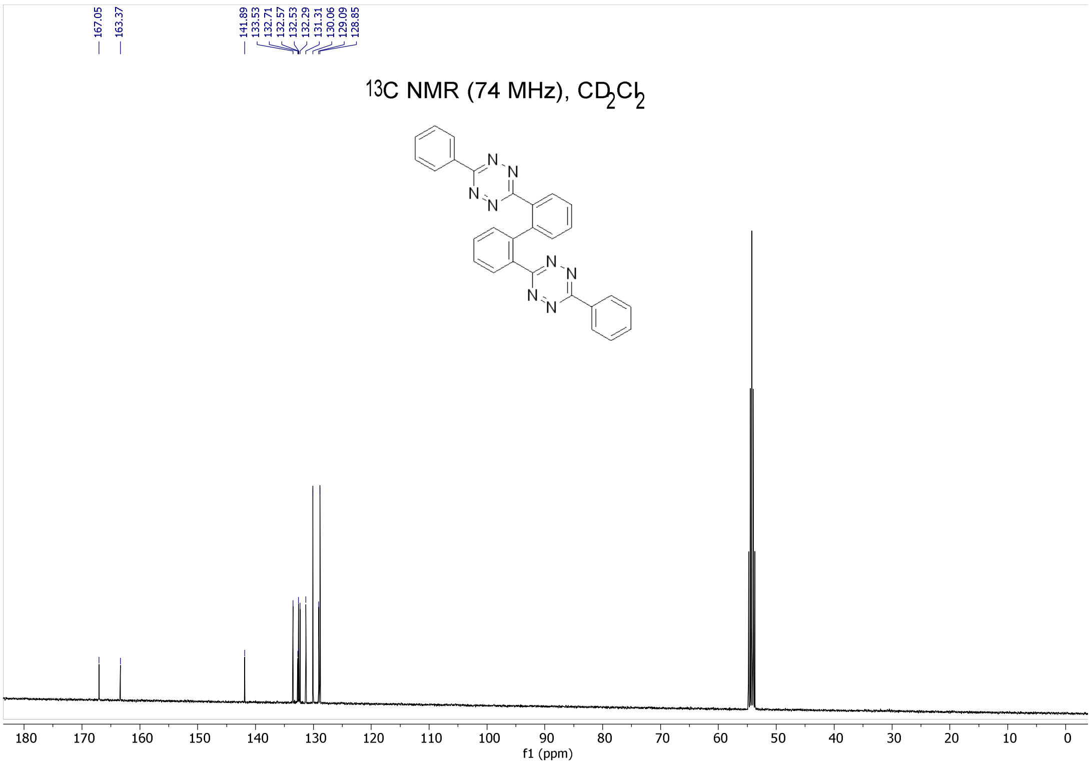

[Ag(3)][PF_6] complex (4)

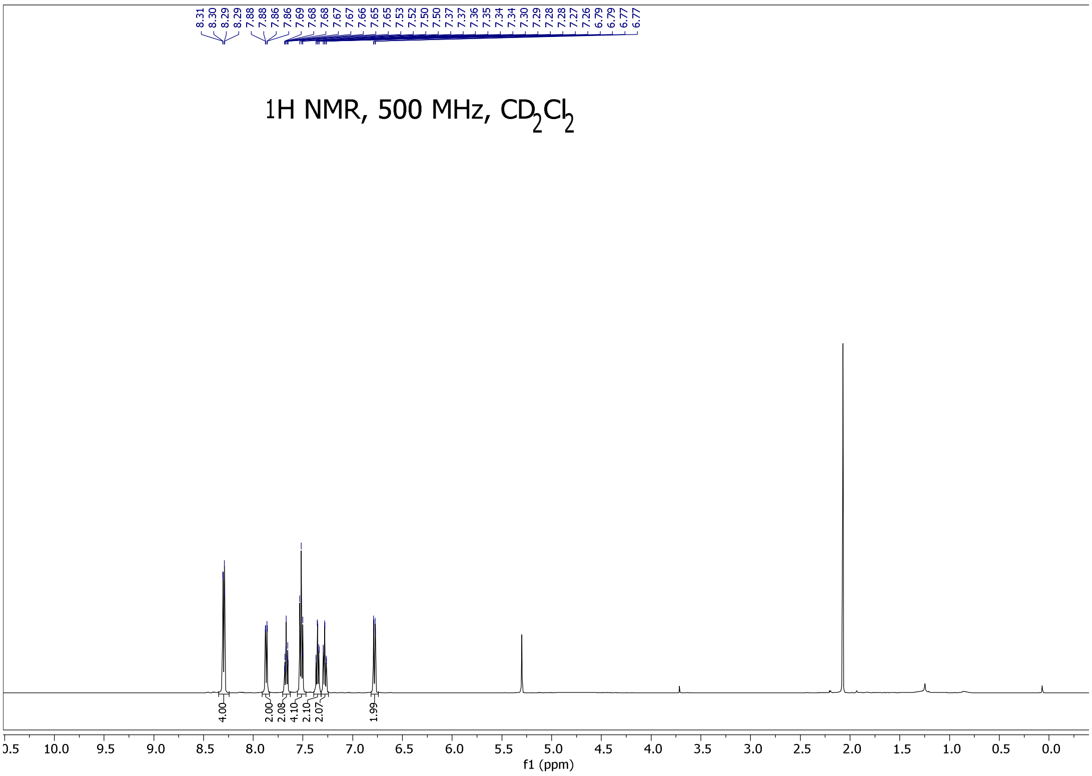

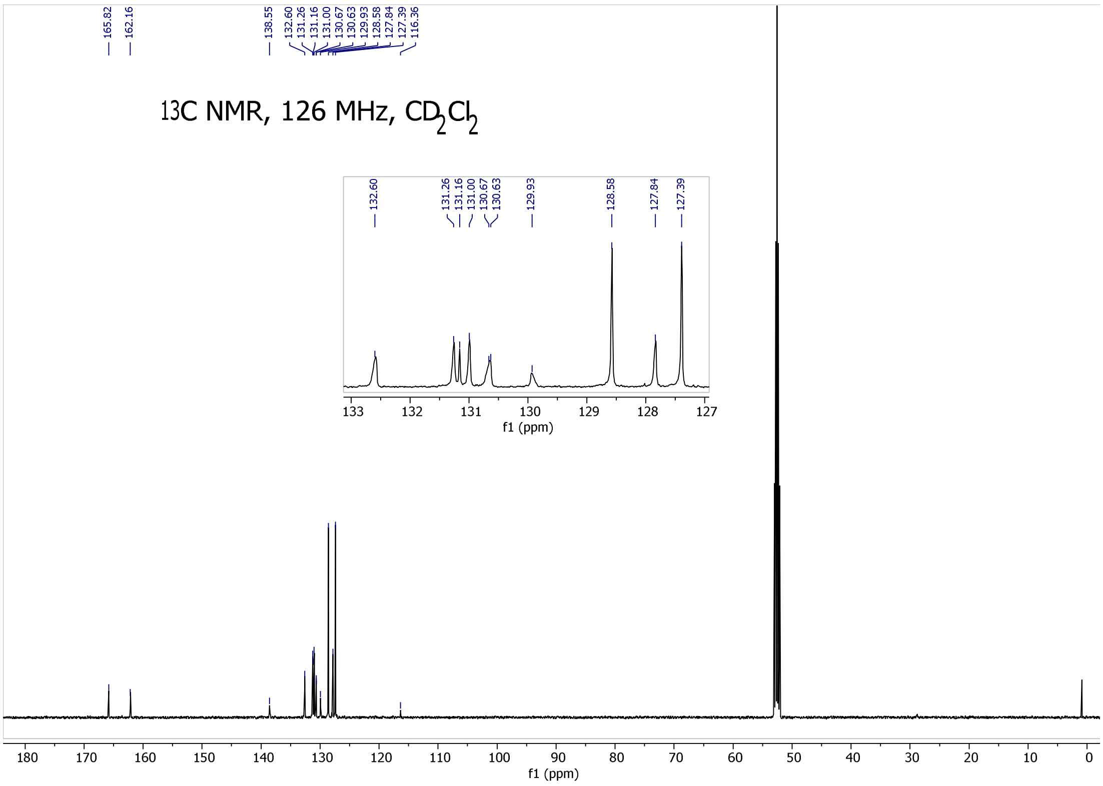

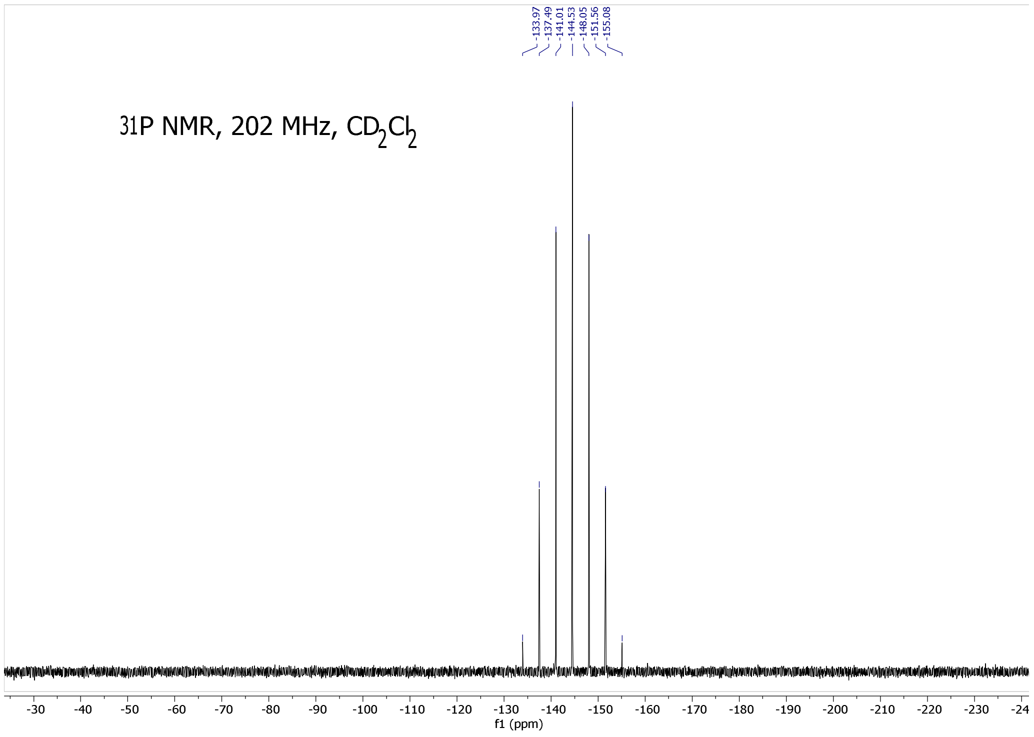
31P NMR, 202 MHz, CD_2Cl_2
-133.97
-137.49
-141.01
-144.53
-148.05
-151.56
-155.08
f1 (ppm)

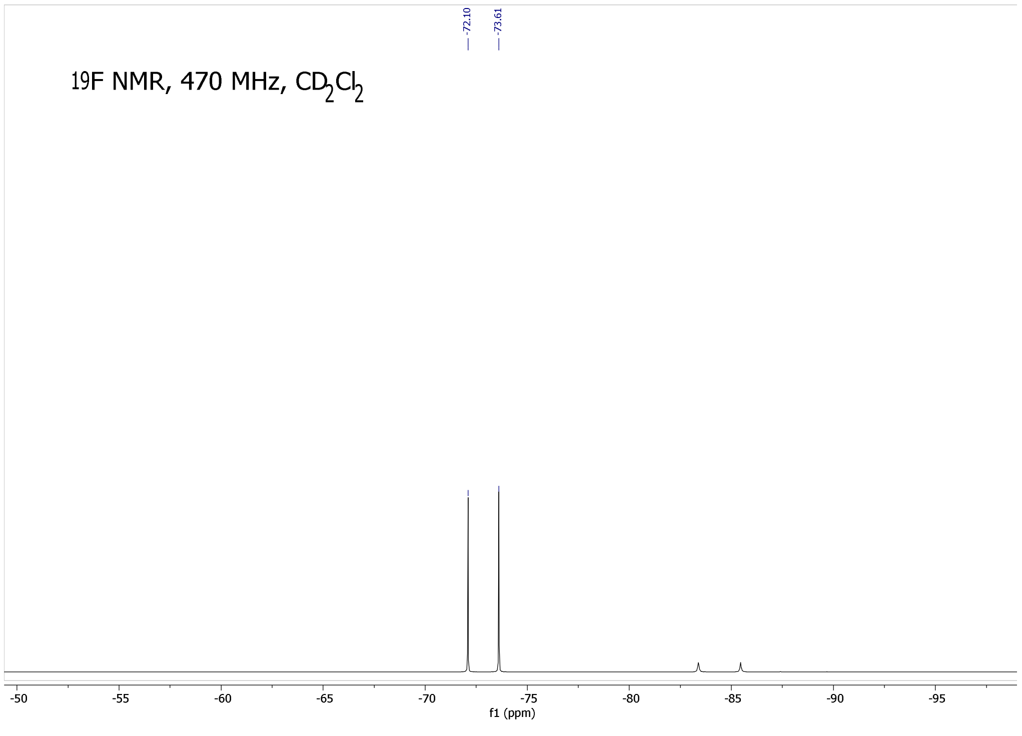
19F NMR, 470 MHz, CD_2Cl_2
-72.10
-73.61
f1 (ppm)

[Ag(3)][PF$_6$]$_\infty$ complex (4a)

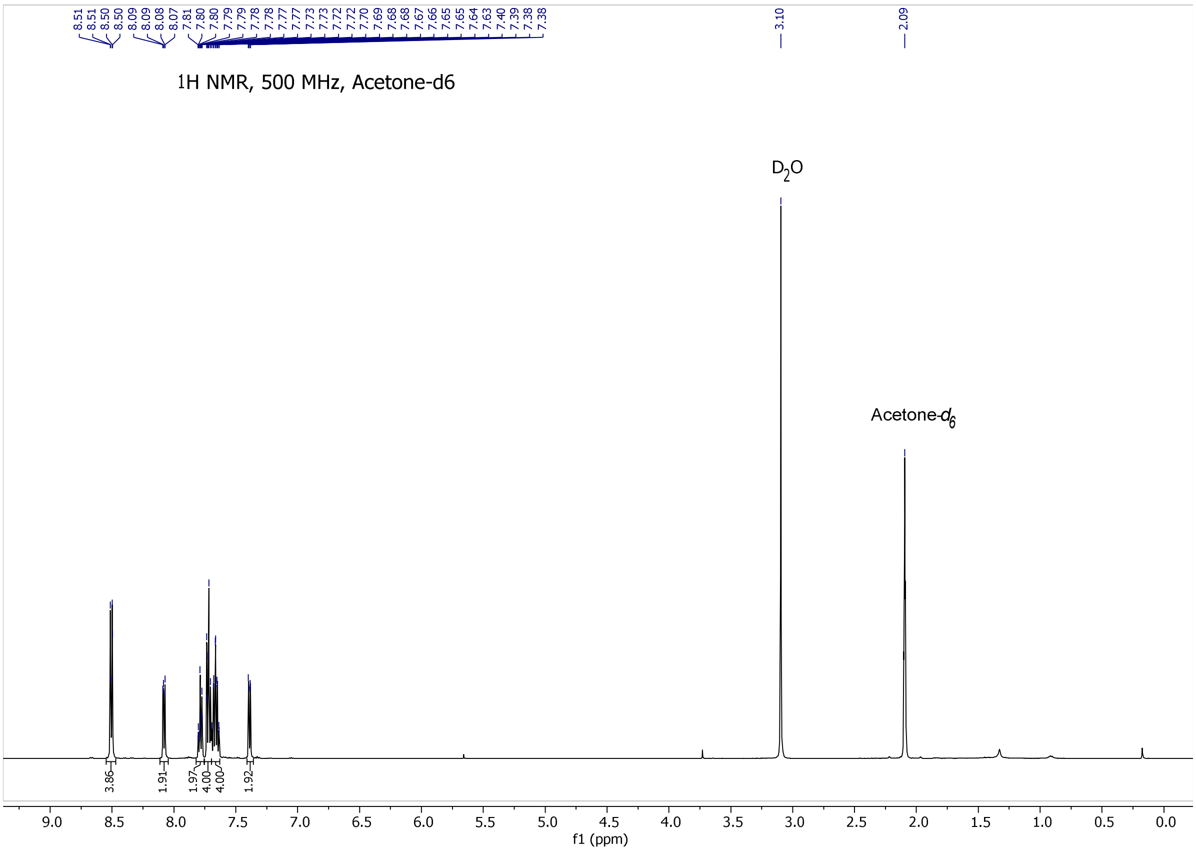

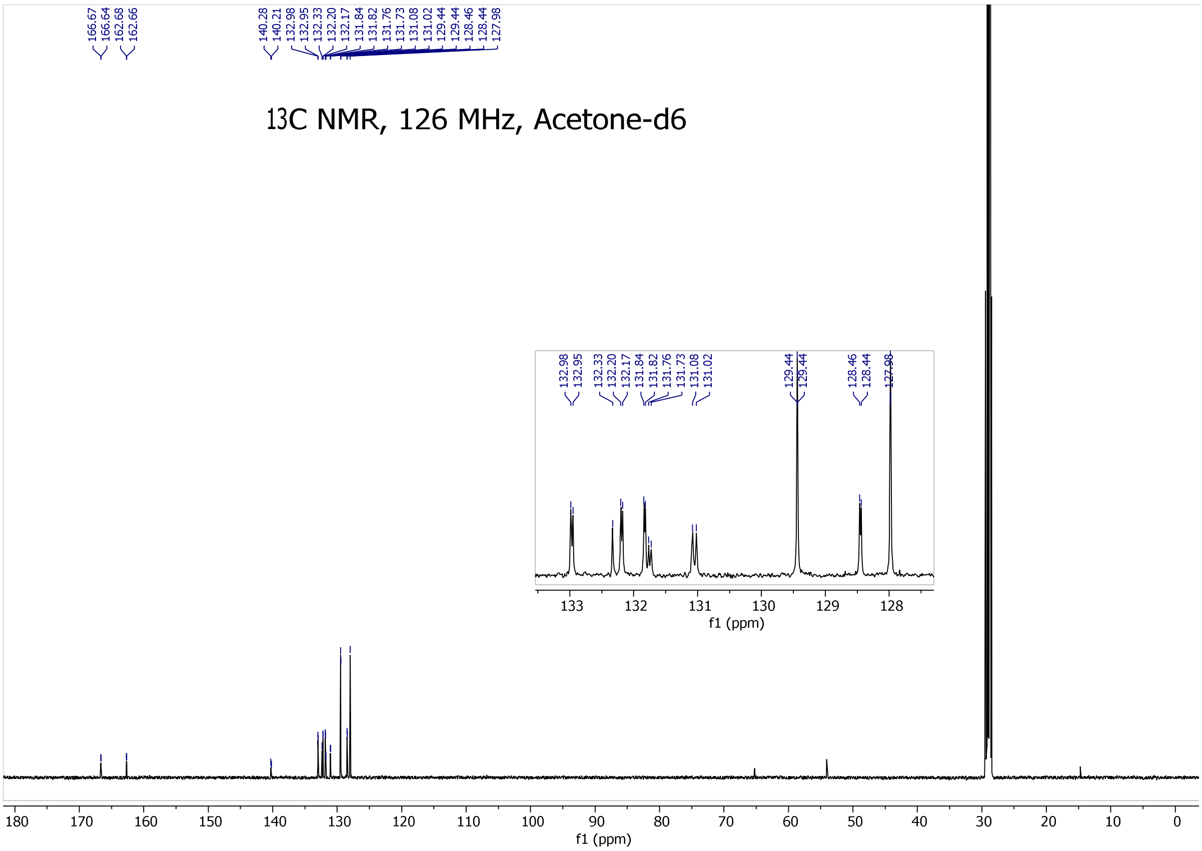

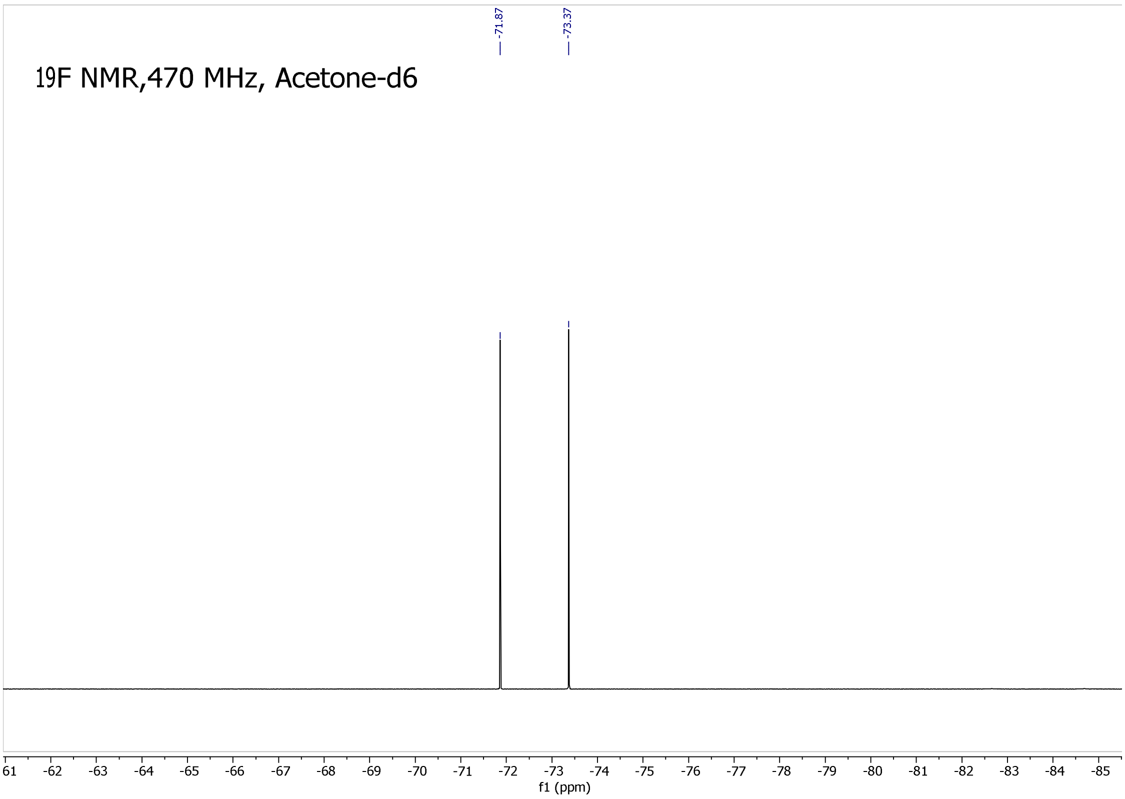

[Ag(3)][BF4] complex (5)

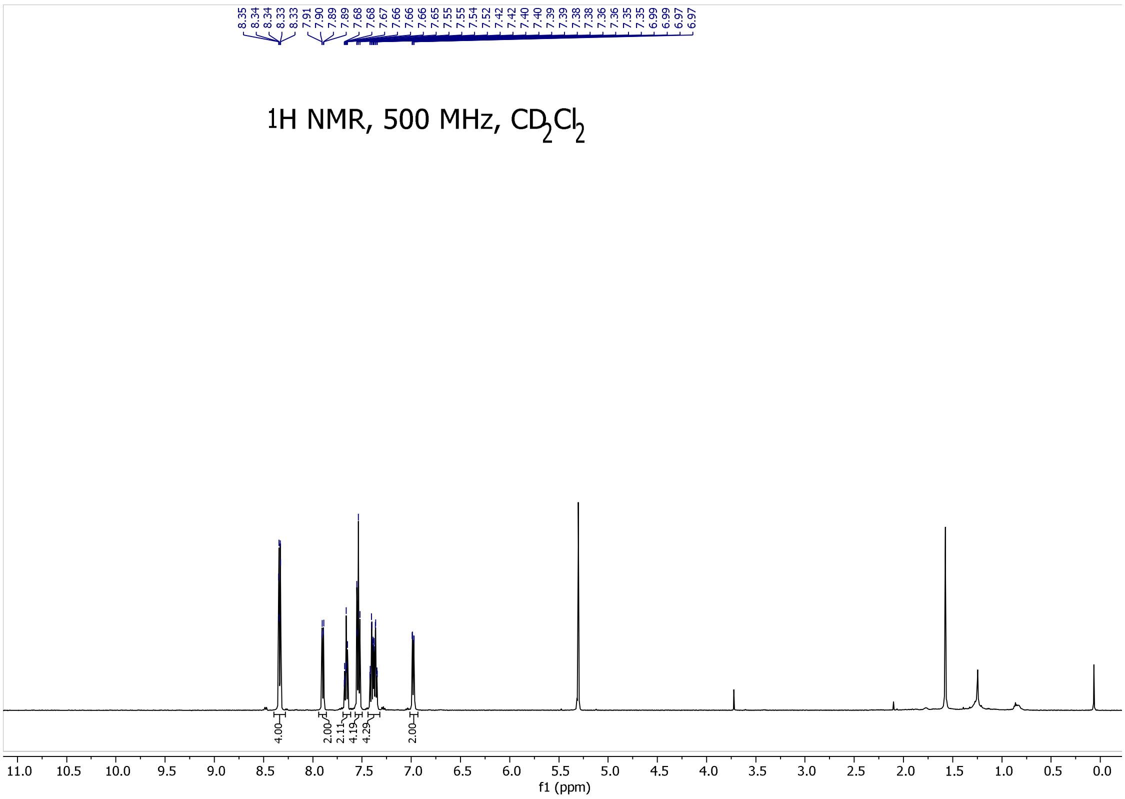

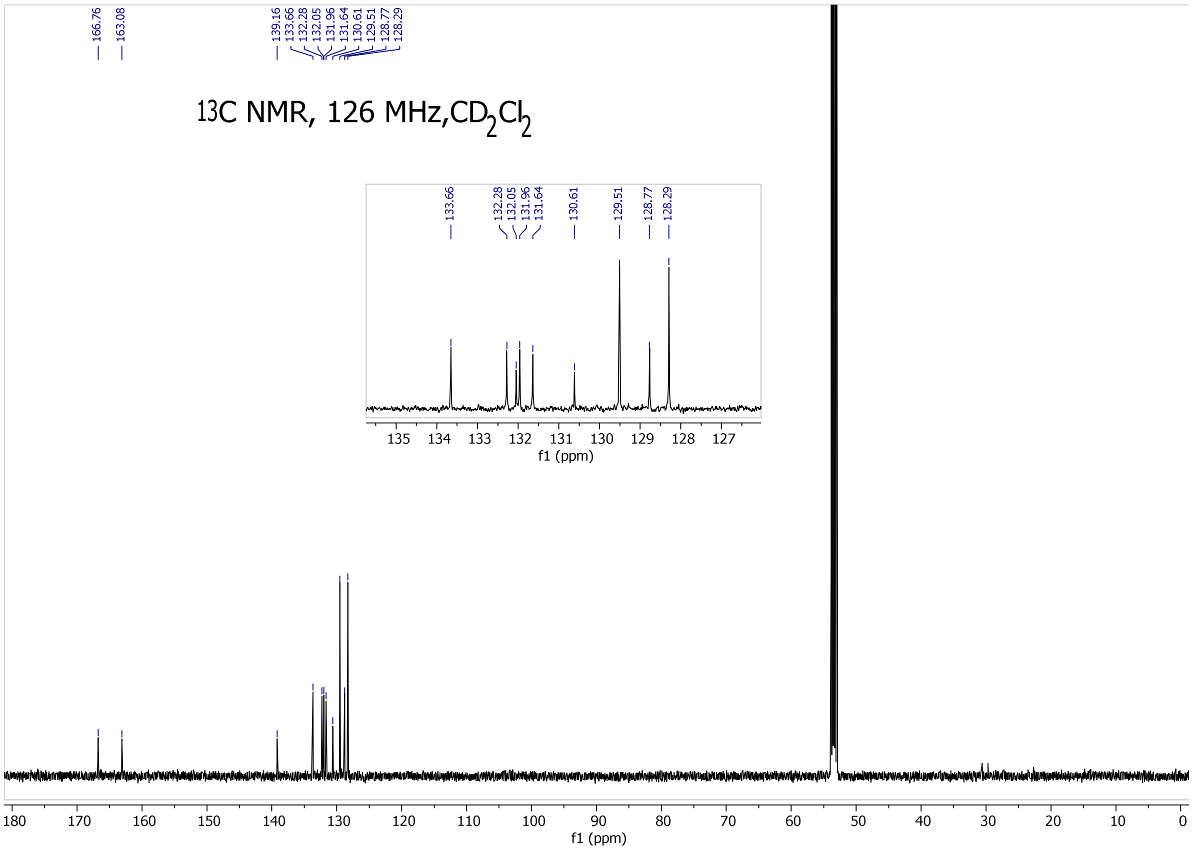

166.76
163.08
139.16
133.66
132.28
132.05
131.96
131.64
130.61
129.51
128.77
128.29
13C NMR, 126 MHz,CD2Cl2
135 134 133 132 131 130 129 128 127
f1 (ppm)
180 170 160 150 140 130 120 110 100 90 80 70 60 50 40 30 20 10 0
f1 (ppm)

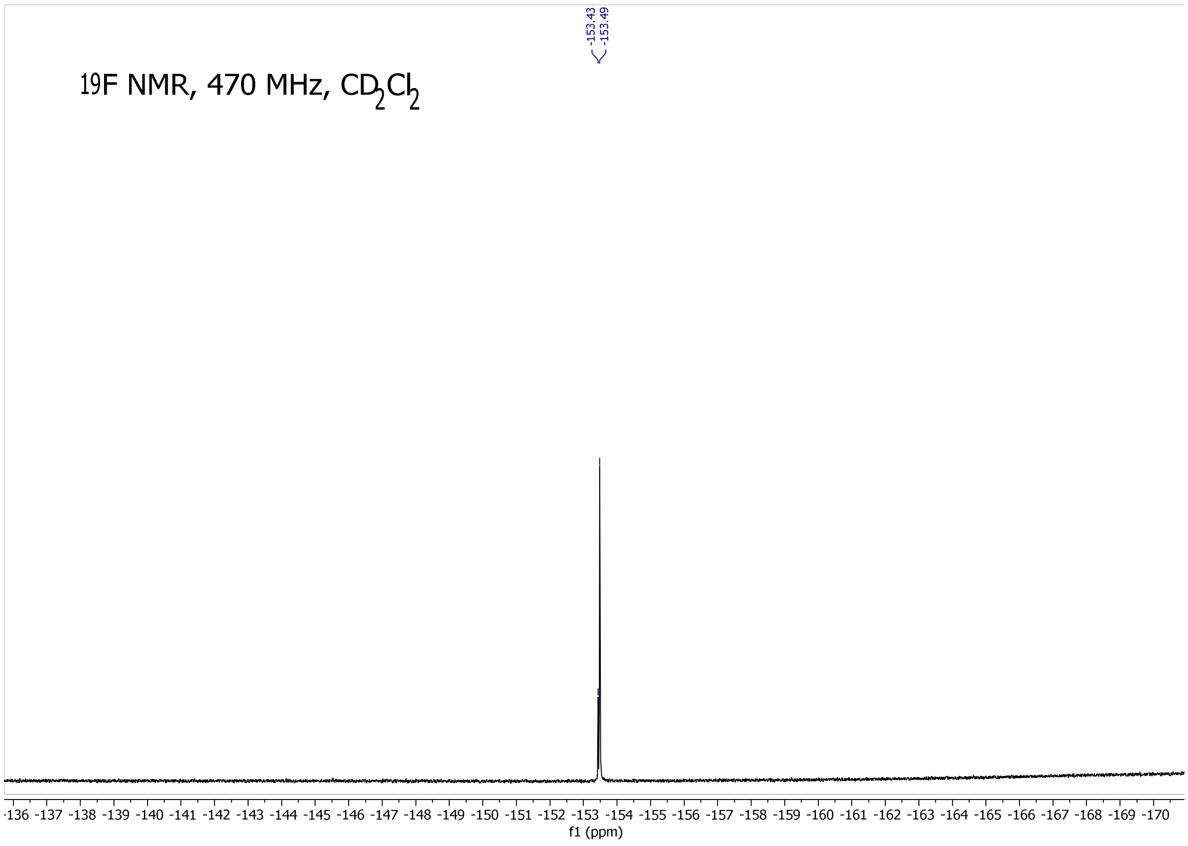

-153.43
-153.49
19F NMR, 470 MHz, CD2Cl2
-136 -137 -138 -139 -140 -141 -142 -143 -144 -145 -146 -147 -148 -149 -150 -151 -152 -153 -154 -155 -156 -157 -158 -159 -160 -161 -162 -163 -164 -165 -166 -167 -168 -169 -170
f1 (ppm)

[Ag(3)][SbF$_6$] complex (6)

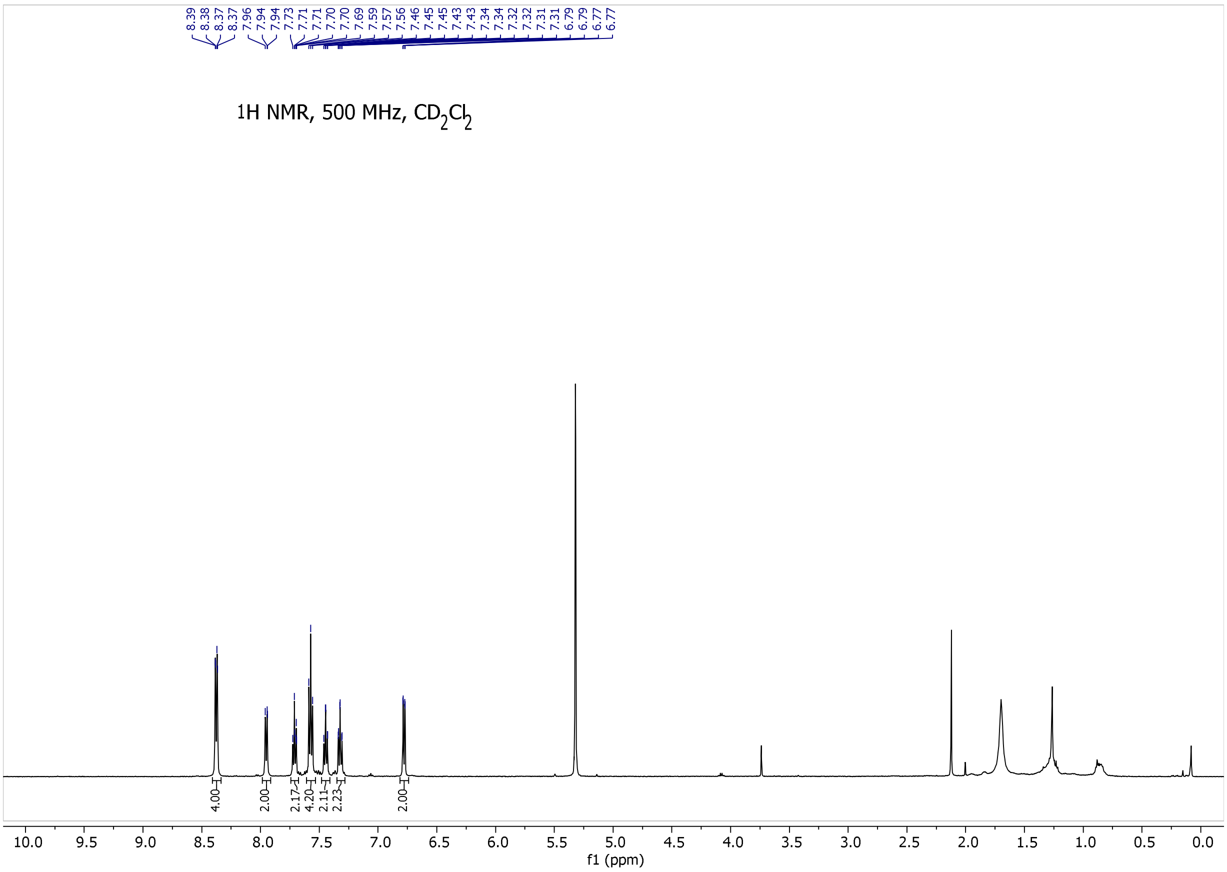

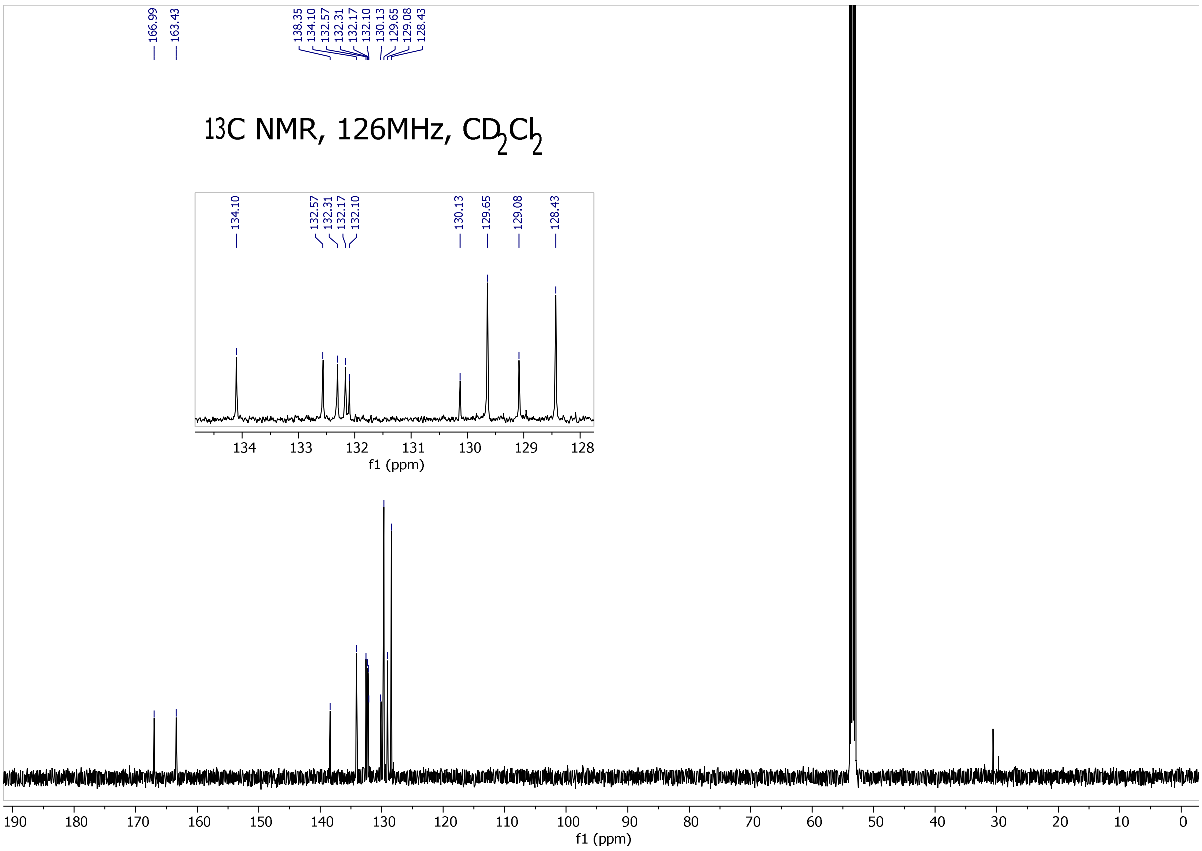

[Ag(3)][ClO$_4$] complex (7)

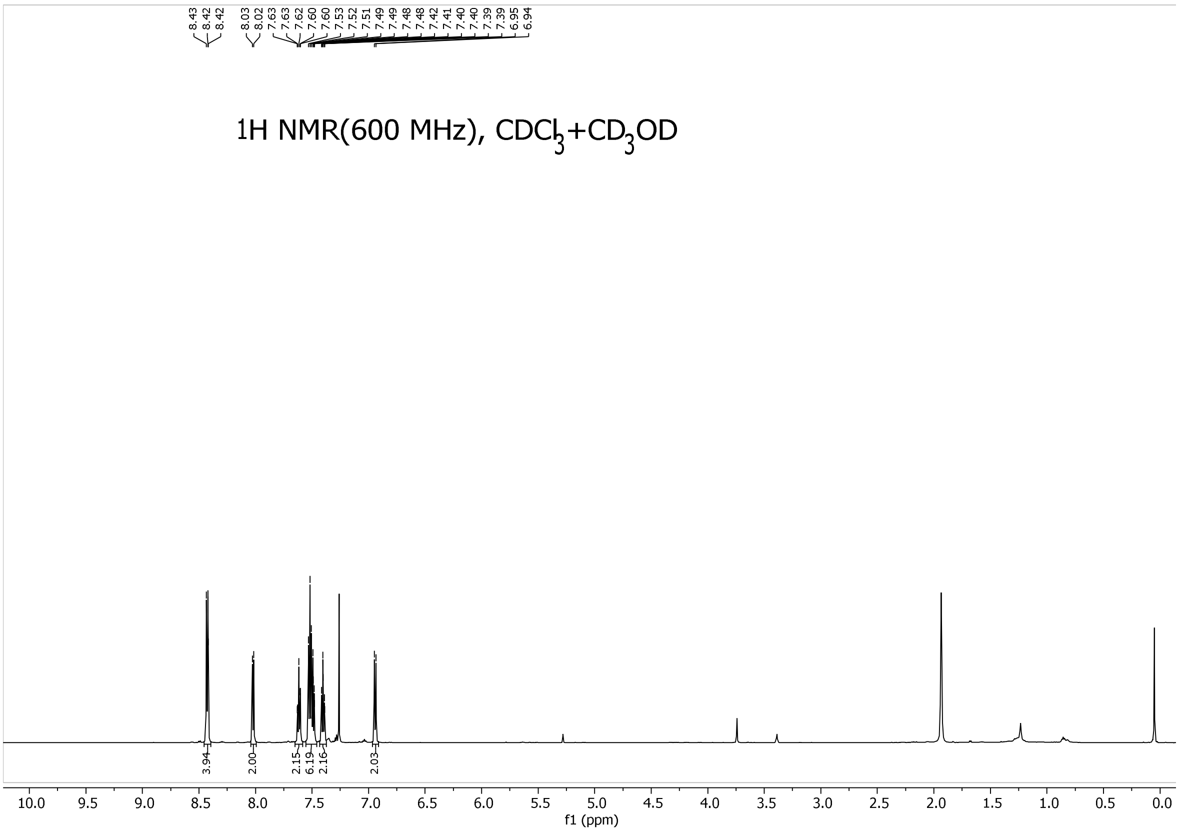

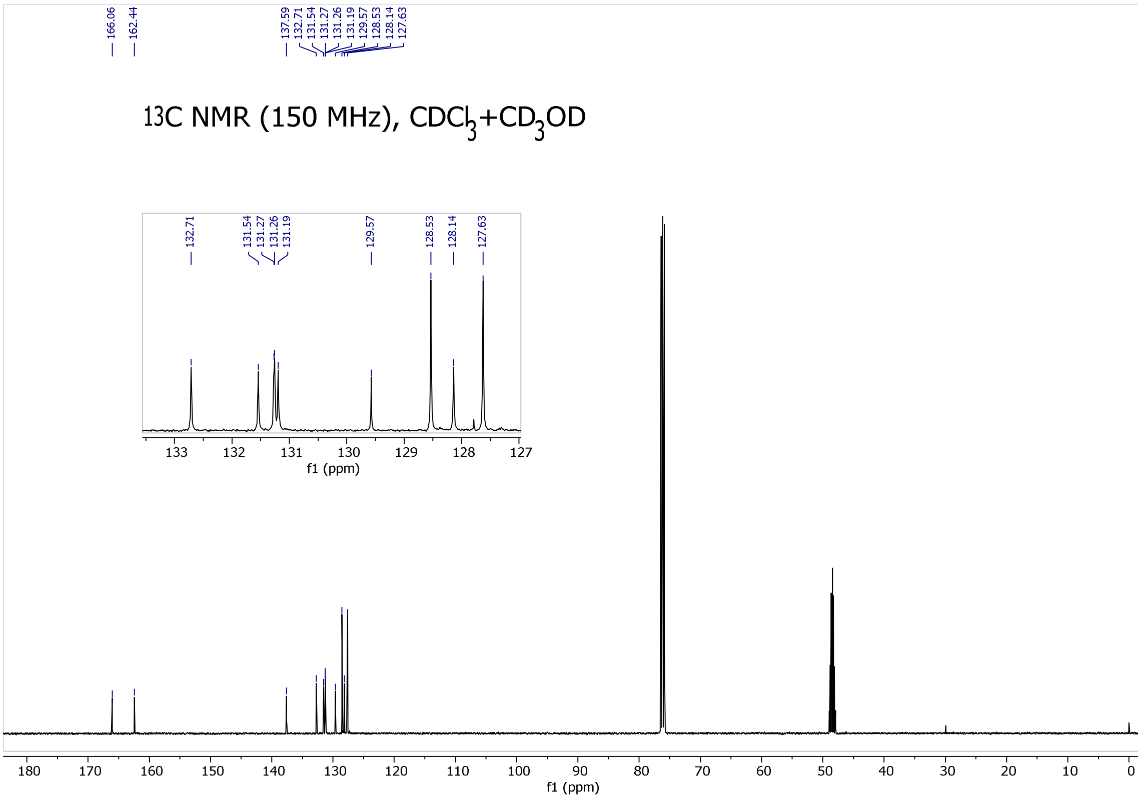

[Ag(3)][NTf2] complex (8)

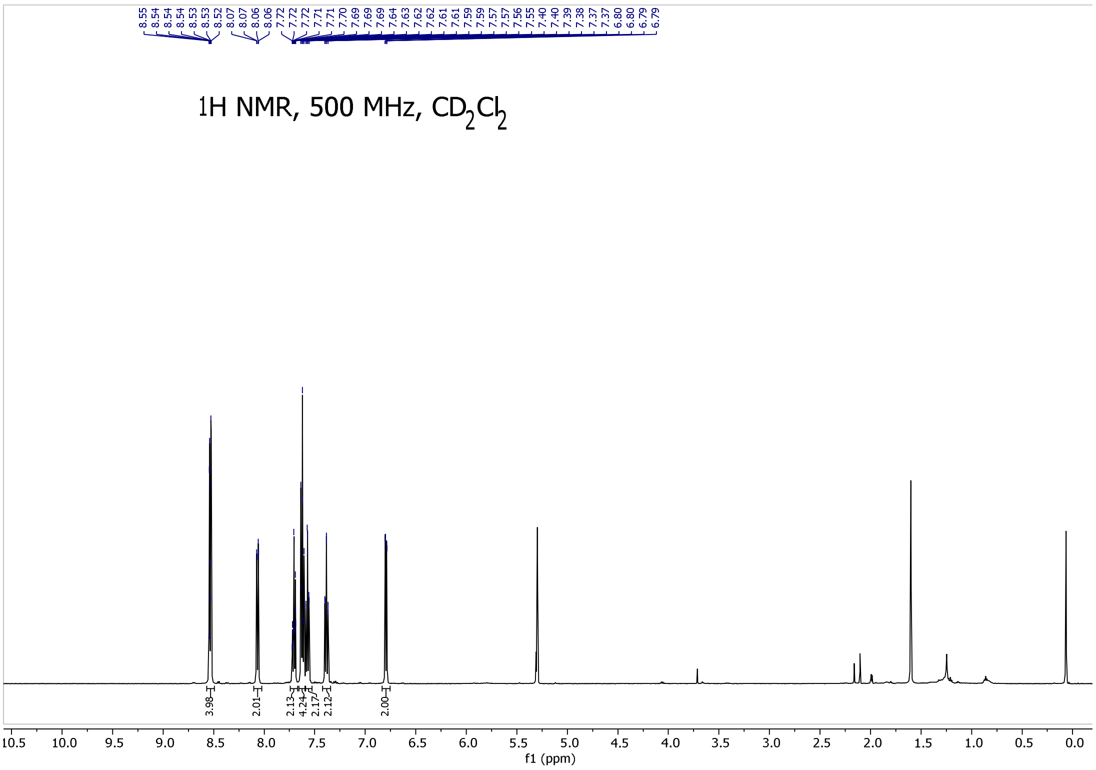

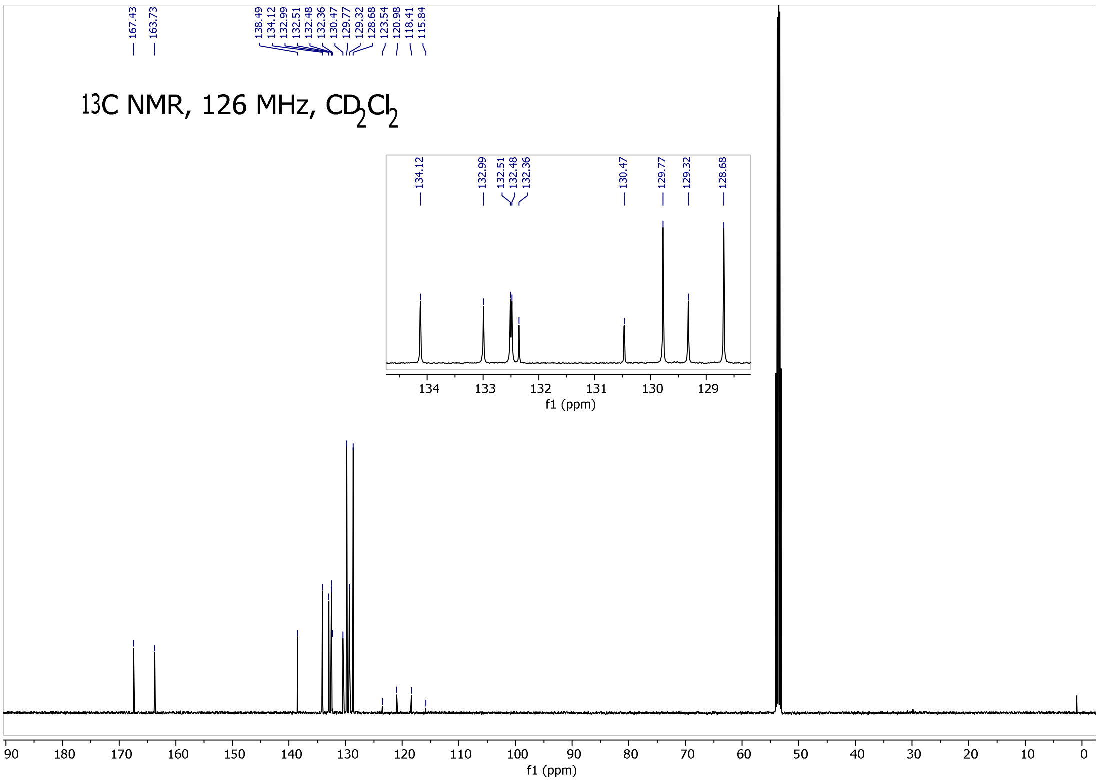

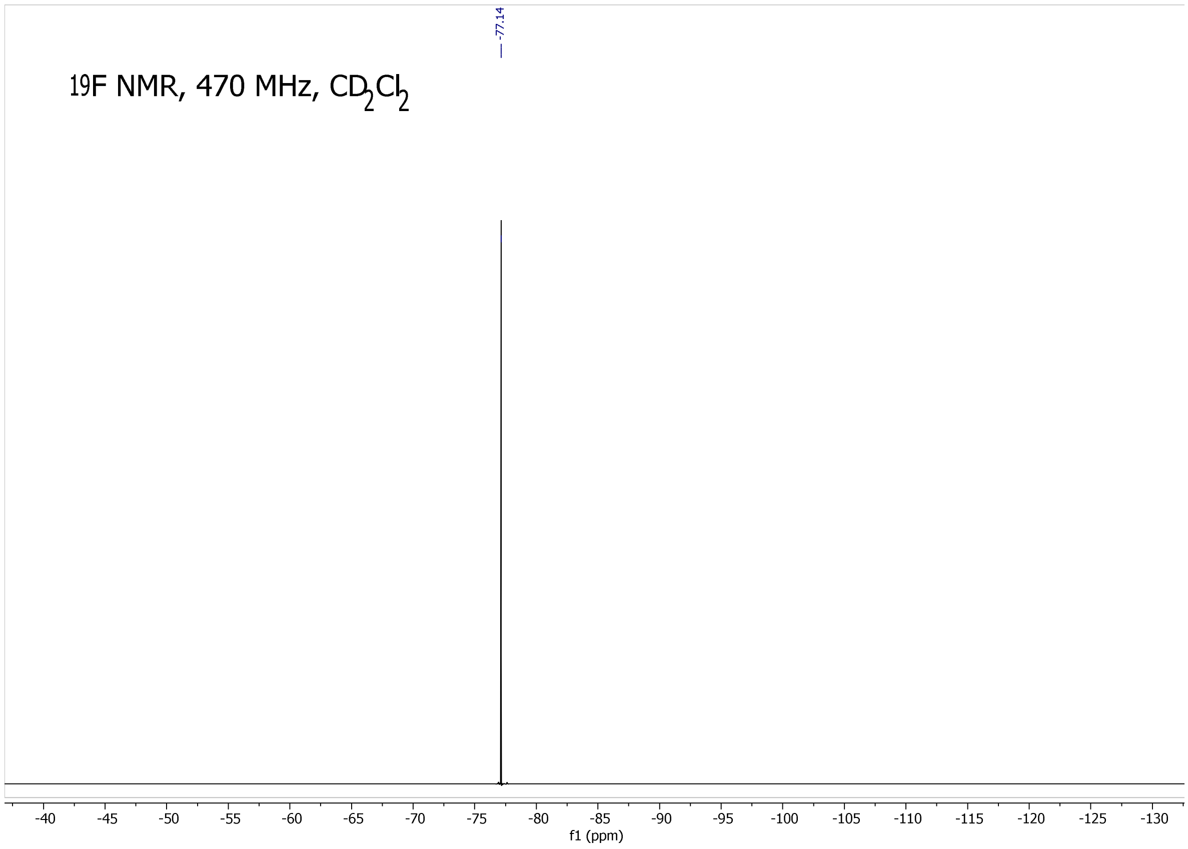

[Ag(3)][OTf] complex (9)

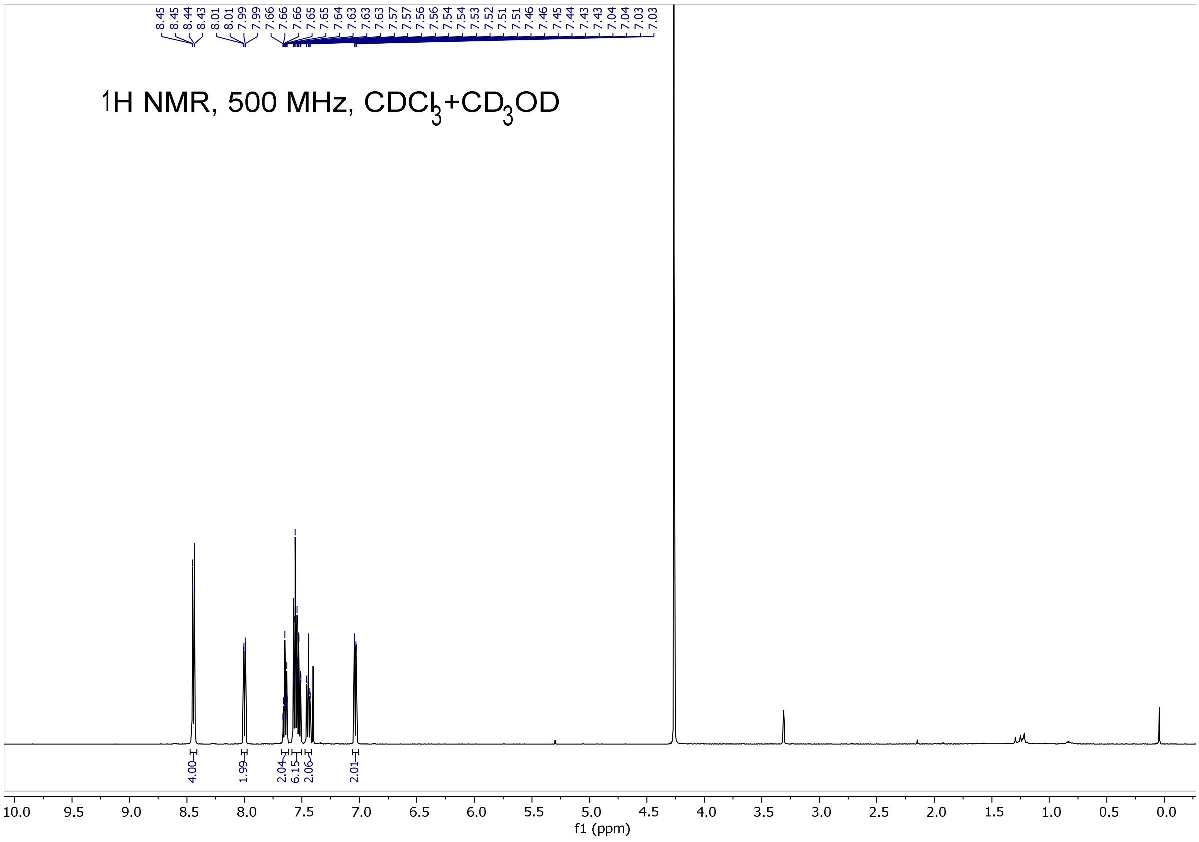

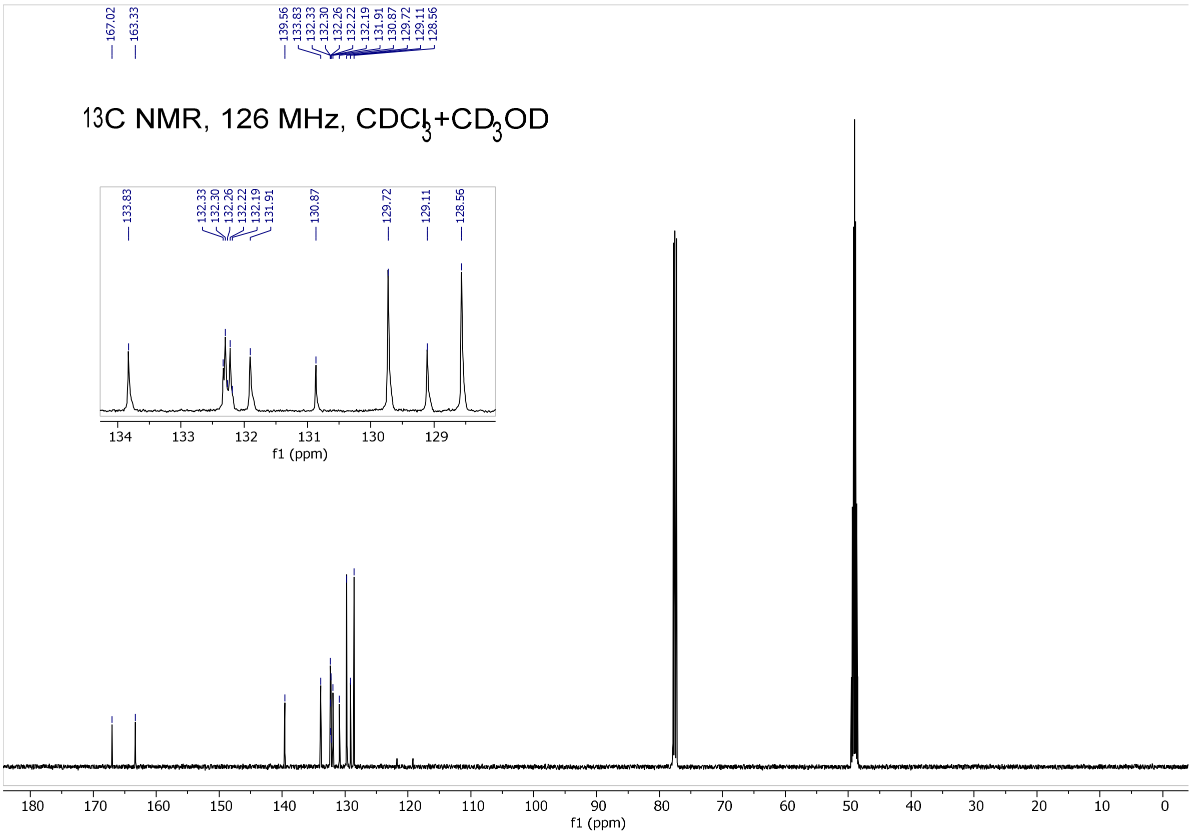
13C NMR, 126 MHz, CDCl3+CD3OD
167.02
163.33
139.56
133.83
132.33
132.30
132.26
132.22
132.19
131.91
130.87
129.72
129.11
128.56
f1 (ppm)

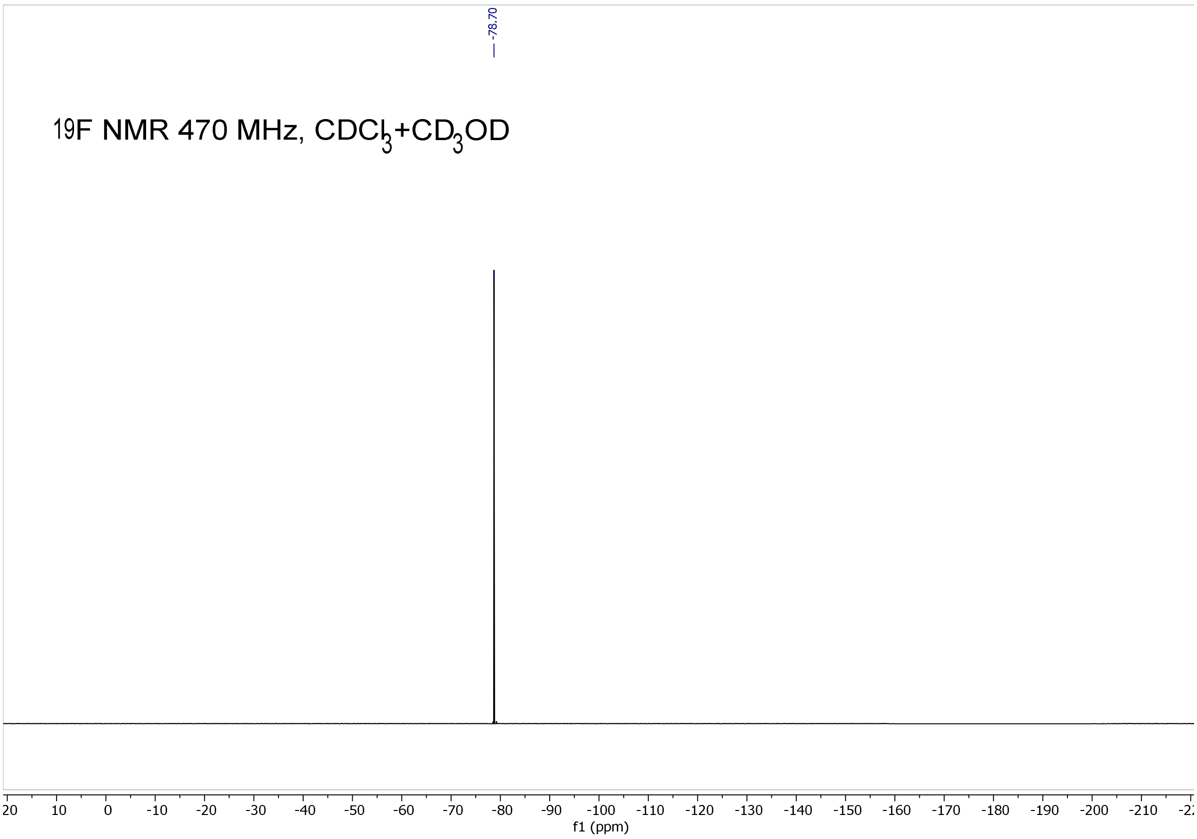
19F NMR 470 MHz, CDCl3+CD3OD
-78.70
f1 (ppm)

[Cu(3)][BF4] complex (10)

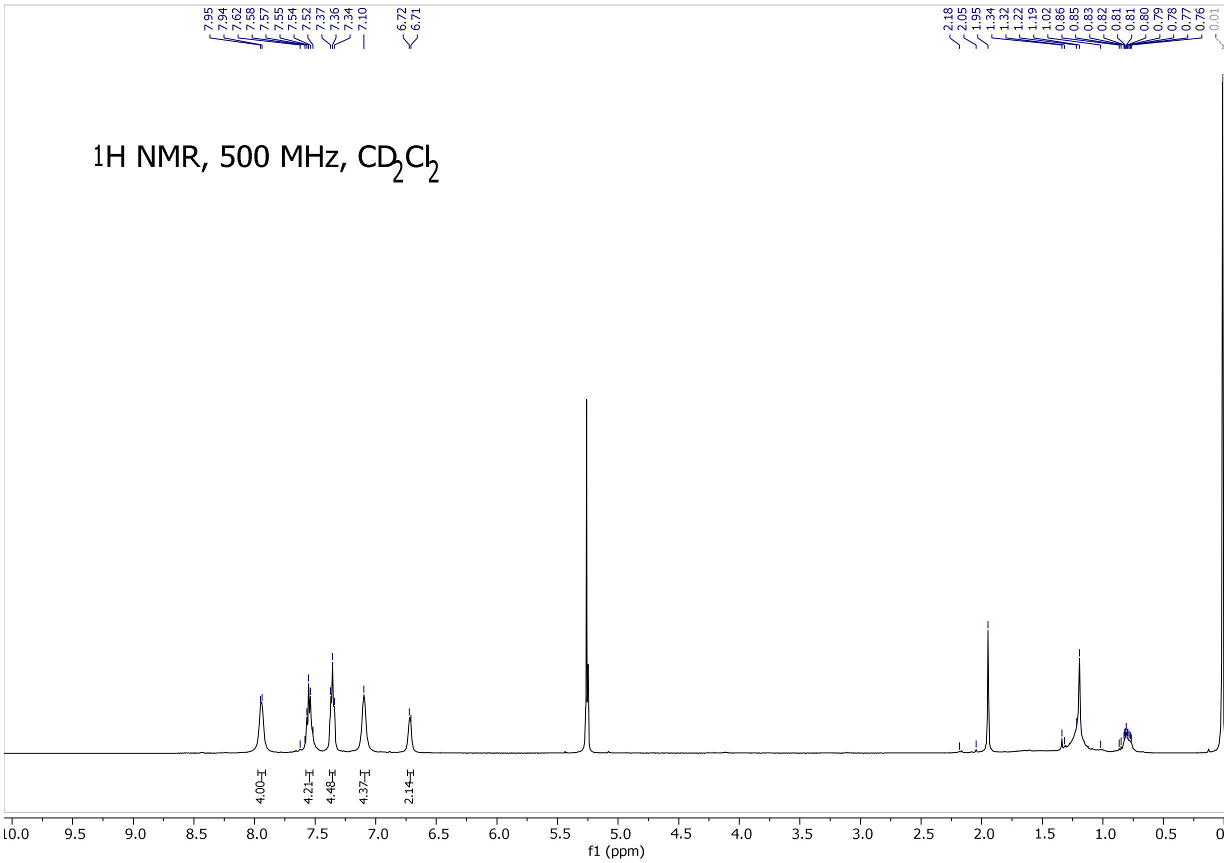

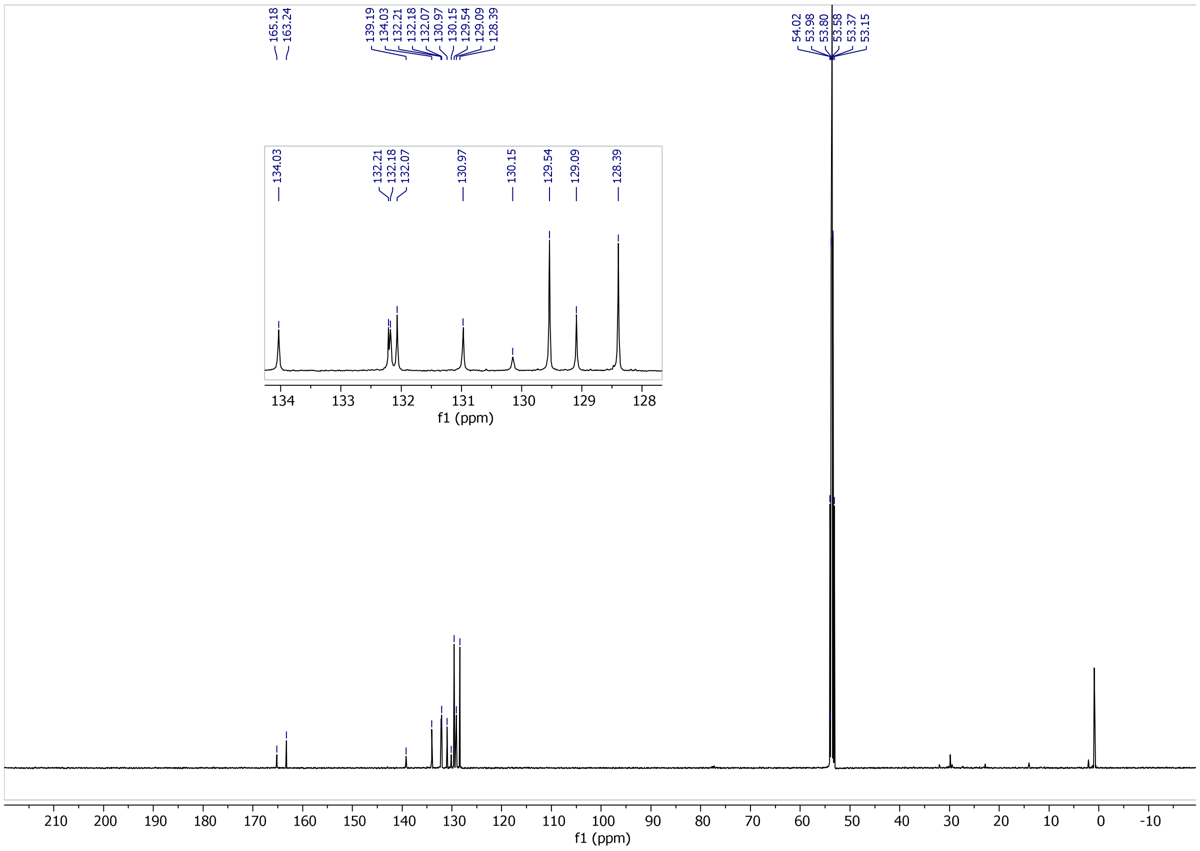

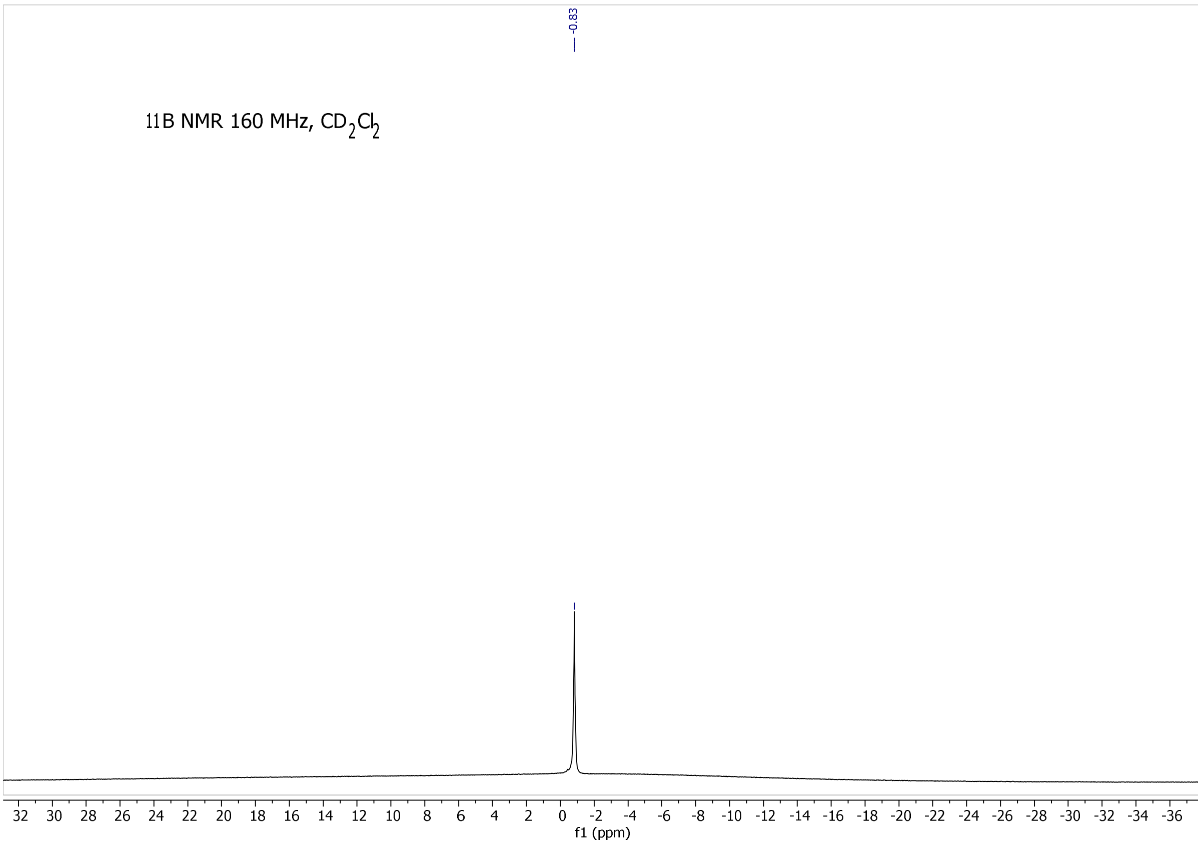
-0.83
11B NMR 160 MHz, CD2Cl2
32 30 28 26 24 22 20 18 16 14 12 10 8 6 4 2 0 -2 -4 -6 -8 -10 -12 -14 -16 -18 -20 -22 -24 -26 -28 -30 -32 -34 -36
f1 (ppm)

Mass spectrum of copper and palladium complexes

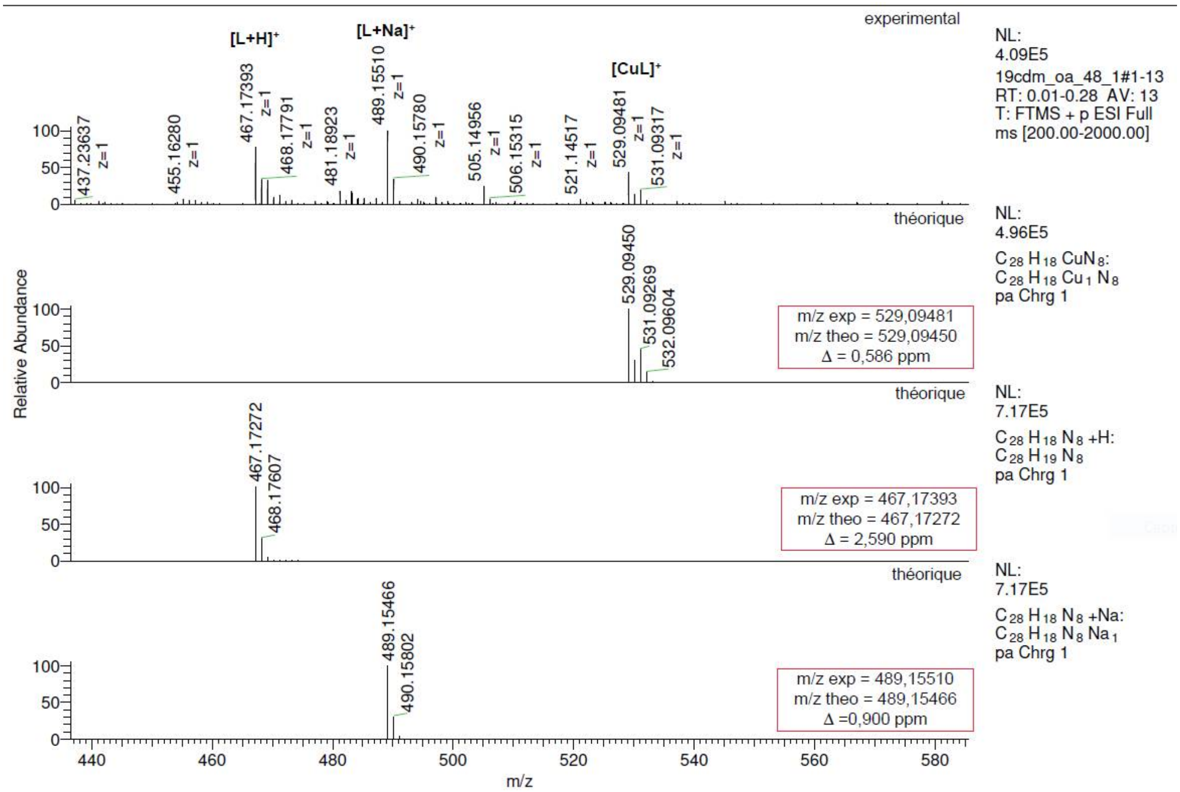

Figure S11. ESI-MS spectrum of $[Cu(\mathbf{3})]^{+}$ complex (**10**).

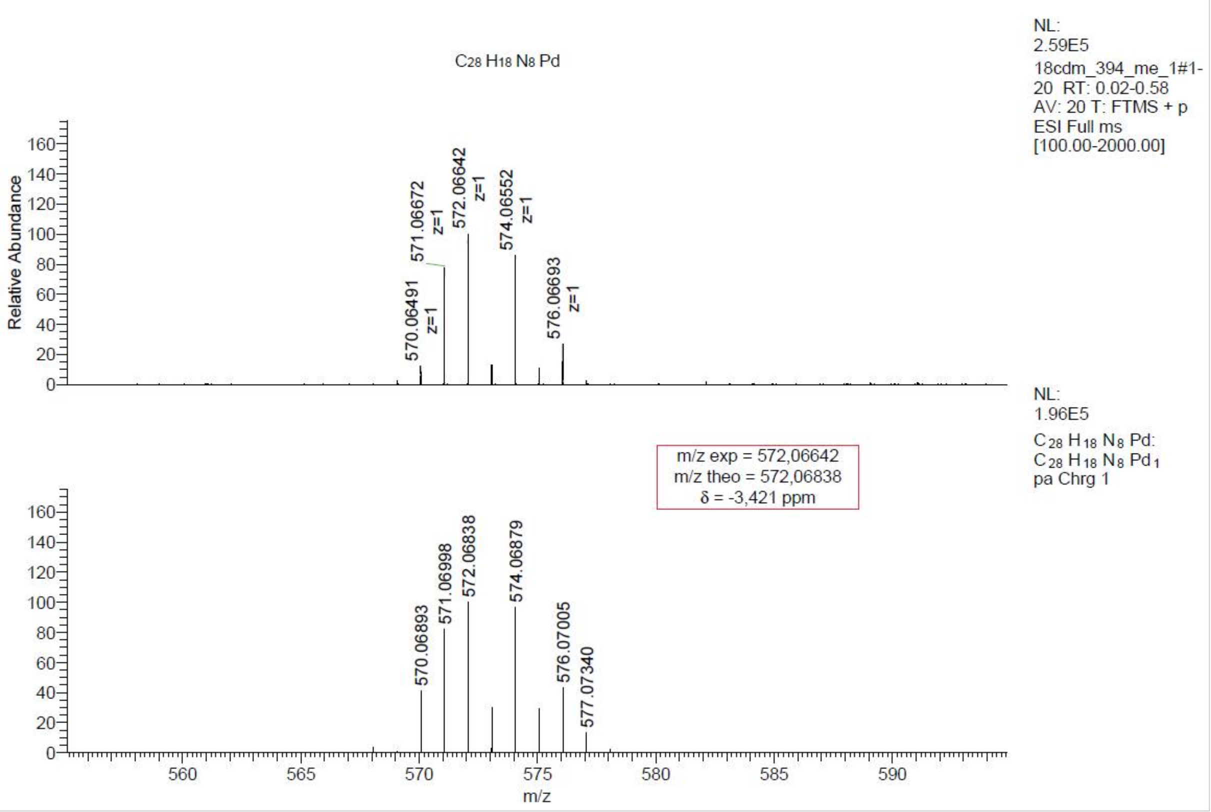

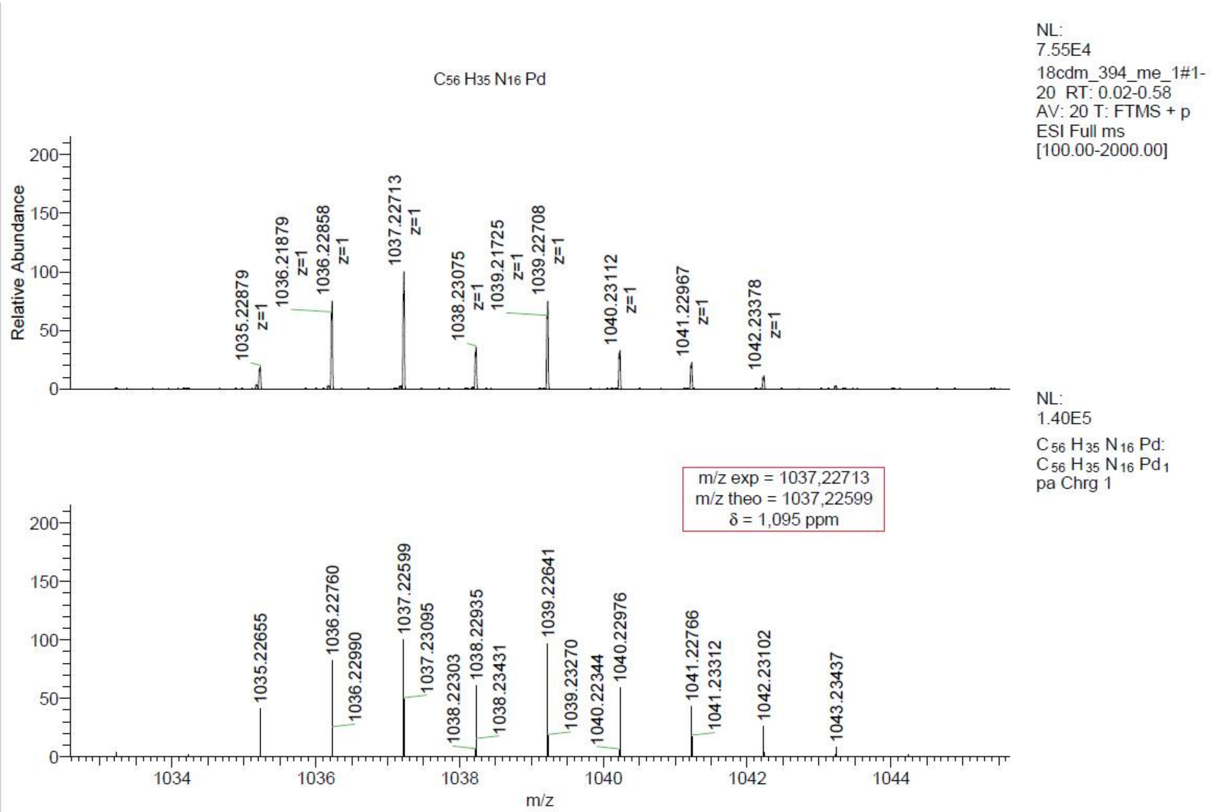

Figure S12. ESI-MS spectrum of [Pd(**3**)] complex (**11**) and related cluster $[Pd\ (\mathbf{3})_2]^+$

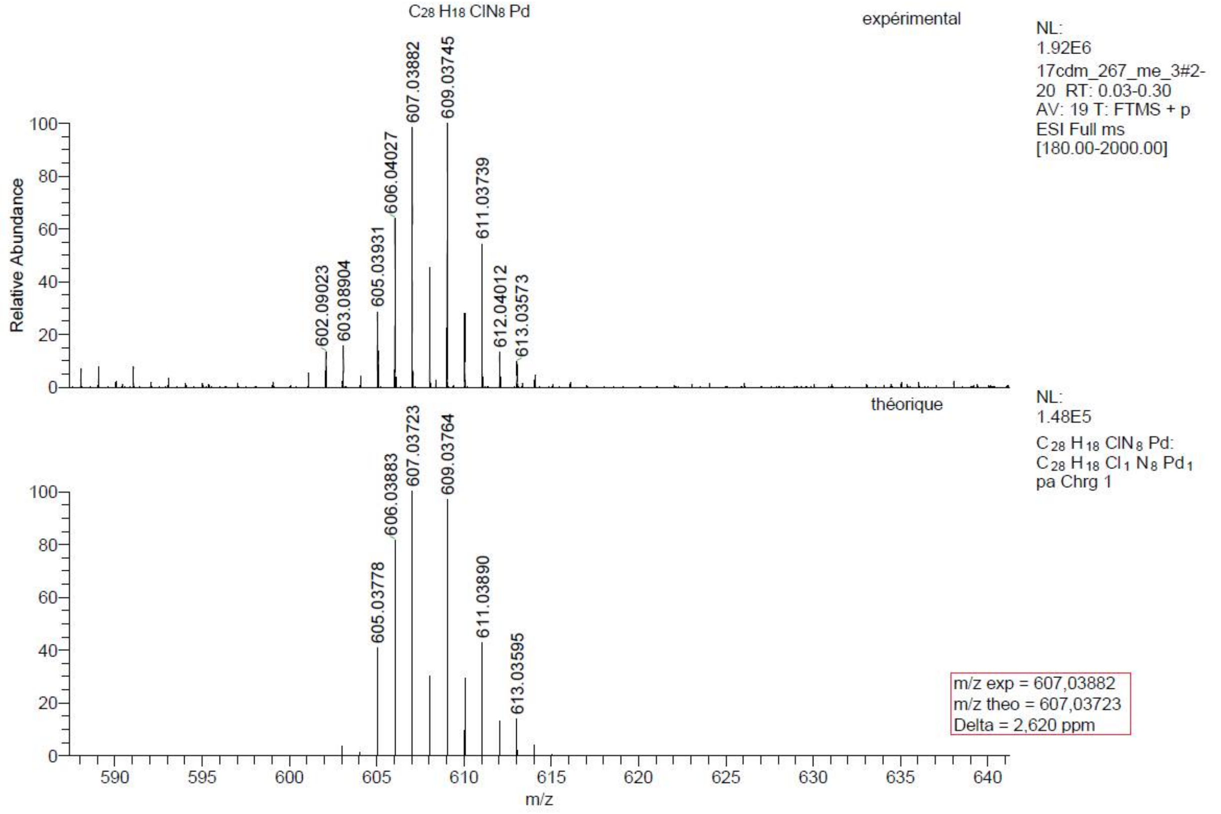

Figure S13. ESI-MS spectrum of [Pd(**3**)Cl] complex (**12**)
